# Design, recruitment, and baseline characteristics of the EMPA-KIDNEY trial

**DOI:** 10.1093/ndt/gfac040

**Published:** 2022-03-03

**Authors:** The EMPA-KIDNEY Collaborative Group, William G Herrington, Christoph Wanner, Jennifer B Green, Sibylle J Hauske, Parminder Judge, Kaitlin J Mayne, Sarah Y A Ng, Emily Sammons, Doreen Zhu, Natalie Staplin, David Preiss, Will Stevens, Karl Wallendszus, Rejive Dayanandan, Carol Knott, Michael Hill, Jonathan Emberson, Susanne Brenner, Vladimir Cejka, Alfred K Cheung, Zhihong Liu, Jing Li, Peiling Chen, Laiseong Hooi, Wen Liu, Takashi Kadowaki, Masaomi Nangaku, Adeera Levin, David Cherney, Roberto Pontremoli, Aldo Pietro Maggioni, Shinya Goto, Aiko Tomita, Rajat Deo, Katherine Tuttle, Jens Eilbracht, Stefan Hantel, Mark Hopley, Martin J Landray, Colin Baigent, Richard Haynes, Colin Baigent, Martin J Landray, Christoph Wanner, William G Herrington, Richard Haynes, Jennifer B Green, Sibylle J Hauske, Martina Brueckmann, Mark Hopley, Susanne Brenner, Alfred K Cheung, David Preiss, Zhihong Liu, Jing Li, Laiseong Hooi, Wen Liu, Takashi Kadowaki, Masaomi Nangaku, Adeera Levin, David Cherney, Roberto Pontremoli, Aldo Pietro Maggioni, Natalie Staplin, Jonathan Emberson, Stefan Hantel, Shinya Goto, Rajat Deo, Katherine Tuttle, Sarah Y A Ng, Francisco Javier Rossello Lozano, Emily Sammons, Doreen Zhu, Peter Sandercock, Rudolf Bilous, Charles Herzog, Paul Whelton, Janet Wittes, Derrick Bennett, Andy Burke, Richard Brown, Rejive Dayanandan, Lucy Fletcher, Hannah Gosling, Emily Harding, Richard Haynes, William G Herrington, Parminder Judge, Carol Knott, Ryonfa Lee, Kevin Murphy, Yanru Qiao, Rachel Raff, Hui Yu, YanRu Qiao, Vladimir Cejka, Marcela Fajardo-Moser, Andrea Lorimer, Donata Lucci, Anita Hepditch, Amanda Axler, Peiling Chen, Dai Hao, Cheng Beng Goh, Sarojini Sivanandam, Akiko Hashimoto, Wakako Negoro, Aiko Tomita, Morisaki Tomoko

**Keywords:** cardiovascular disease, CKD, clinical trial, empagliflozin, sodium-glucose co-transporter 2 inhibitor

## Abstract

**Background:**

The effects of the sodium-glucose co-transporter 2 inhibitor empagliflozin on renal and cardiovascular disease have not been tested in a dedicated population of people with chronic kidney disease (CKD).

**Methods:**

The EMPA-KIDNEY trial is an international randomized, double-blind, placebo-controlled trial assessing whether empagliflozin 10 mg daily decreases the risk of kidney disease progression or cardiovascular death in people with CKD. People with or without diabetes mellitus (DM) were eligible provided they had an estimated glomerular filtration rate (eGFR) ≥20 but <45 mL/min/1.73 m^2^ or an eGFR ≥45 but <90 mL/min/1.73 m^2^ with a urinary albumin:creatinine ratio (uACR) ≥200 mg/g. The trial design is streamlined, as extra work for collaborating sites is kept to a minimum and only essential information is collected.

**Results:**

Between 15 May 2019 and 16 April 2021, 6609 people from eight countries in Europe, North America and East Asia were randomized. The mean age at randomization was 63.8 years [standard deviation (SD) 13.9)], 2192 (33%) were female and 3570 (54%) had no prior history of DM. The mean eGFR was 37.5 mL/min/1.73 m^2^ (SD 14.8), including 5185 (78%) with an eGFR <45 mL/min/1.73 m^2^. The median uACR was 412 mg/g) (quartile 1–quartile 3 94–1190), with a uACR <300 mg/g in 3194 (48%). The causes of kidney disease included diabetic kidney disease [*n* = 2057 (31%)], glomerular disease [*n* = 1669 (25%)], hypertensive/renovascular disease [*n* = 1445 (22%)], other [*n* = 808 (12%)] and unknown causes [n = 630 (10%)].

**Conclusions:**

EMPA-KIDNEY will evaluate the efficacy and safety of empagliflozin in a widely generalizable population of people with CKD at risk of kidney disease progression. Results are anticipated in 2022.

## Graphical Abstract

**Figure:**
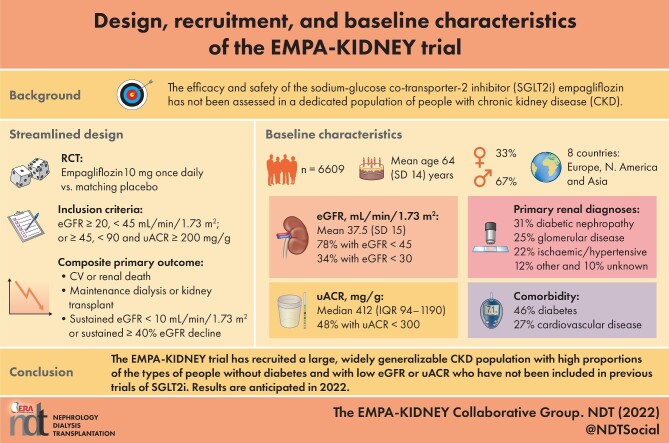

KEY LEARNING POINTS **What is already known about this subject?**

- Prevention of kidney disease progression and decreasing cardiovascular risk are unmet clinical needs among people with chronic kidney disease (CKD).
- In people with CKD, CREDENCE and DAPA-CKD trial results demonstrated that sodium-glucose co-transporter 2 (SGLT-2) inhibitors decrease the risk of kidney disease progression in people with type 2 diabetes mellitus (DM) and a urinary albumin:creatinine ratio (uACR) ≥200 mg/g.
- Prespecified subgroup analyses from DAPA-CKD also found such benefits extend to certain types of people with CKD and uACR ≥200 mg/g in the absence of DM.

**What this study adds?**

- EMPA-KIDNEY is a streamlined double-blind randomized trial designed to assess the effect of empagliflozin 10 mg versus matching placebo, on top of standard of care, on a composite primary outcome of kidney disease progression or cardiovascular death in a CKD population. [Kidney disease progression is defined as end-stage kidney disease, a sustained decline in estimated glomerular filtration rate (eGFR) to <10 mL/min/1.73 m^2^,  renal death or a sustained decline of ≥40% in eGFR from randomization.]
- The EMPA-KIDNEY trial has wide eligibility criteria and has recruited a broad range of people with CKD, including those underrepresented in reported SGLT-2 inhibitor trials. Of the 6609 randomized EMPA-KIDNEY participants, 54% had no prior history of DM at recruitment, 78% had an eGFR <45 mL/min/1.73 m^2^  and 48% had a uACR <300 mg/g.

**What impact this may have on practice or policy?**

- EMPA-KIDNEY will help extend findings from CREDENCE/DAPA-CKD and will add new information about the efficacy and safety of empagliflozin in people with CKD.

## INTRODUCTION

Chronic kidney disease (CKD) is often a progressive condition, with albuminuria representing an important risk factor for a more rapid decline in kidney function [1]. Slowing of progression and avoidance of end-stage kidney disease (ESKD, also referred to as kidney failure [2]), is highly desirable due to the associated excess morbidity and mortality, adverse effects on quality of life and the substantial costs of renal replacement therapy. Inhibitors of the renin–angiotensin system (RAS) decrease albuminuria and slow the rate of progression in albuminuric kidney diseases [3–5]. People with CKD are also at increased risk of cardiovascular disease [6, 7], a key feature of which is structural heart disease, heart failure and sudden death [8–10]. Increased risk of coronary heart disease also accompanies CKD [6]. Lowering blood pressure (BP) and low-density lipoprotein cholesterol in people with CKD decrease cardiovascular risk [11, 12], but despite such interventions, substantial residual risk remains. There is a need for new treatments that can be safely added to the current standard of care to slow the progression to ESKD and decrease cardiovascular risk in CKD.

Inhibitors of sodium-glucose cotransporter 2 (SGLT-2) cause urinary glucose and increased sodium excretion and can decrease weight and BP as well as glycosylated haemoglobin (HbA1c) [13]. The EMPA-REG OUTCOME trial of empagliflozin was the first large-scale placebo-controlled cardiovascular outcome trial to report the effects of an SGLT-2 inhibitor in type 2 diabetes mellitus (DM) [14]. Results showed empagliflozin could favourably decrease not only the risk of atherosclerotic cardiovascular disease, but also the risk of hospitalization for heart failure and the development or worsening of nephropathy [15]. Since then, several other large placebo-controlled cardiovascular outcome trials with other SGLT-2 inhibitors have reported analogous findings [14, 16, 17]. Trials of SGLT-2 inhibitors in patients with heart failure or CKD have also reported relative risk reductions for renal and heart failure outcomes that are similar in size in people with and without DM [18–22].

The CREDENCE and DAPA-CKD placebo-controlled trials in dedicated CKD populations were both stopped early for efficacy following recommendations from their data monitoring committees (DMCs) [18, 23, 24]. CREDENCE demonstrated that canagliflozin 100 mg decreased the risk of kidney disease progression or cardiovascular death in a population of 4401 adults with type 2 DM and albuminuric diabetic kidney disease [renal inclusion criteria: estimated glomerular filtration rate (eGFR) ≥30 but <90 mL/min/1.73 m^2^ and urinary albumin:creatinine ratio (uACR) >300 but ≤5000 mg/g on stable maximum tolerated RAS inhibition] [23]. DAPA-CKD demonstrated that dapagliflozin 10 mg decreased the risk of kidney disease progression or cardiovascular death in a population of 4304 adults with an eGFR ≥ 25 but ≤75 mL/min/1.73 m^2^ and uACR ≥200 but ≤5000 mg/g with or without type 2 DM (98% of whom were taking a RAS inhibitor) [18, 24]. Subgroup analyses from DAPA-CKD found that renal benefits also extend to certain types of non-diabetic causes of albuminuric CKD, including glomerulonephritis not treated with immunosuppression [25–27]. Further assessment of the effects of SGLT-2 inhibitors in people with CKD remains important as DAPA-CKD reported limited information about effects in people without DM [25], and excluded certain causes of kidney disease, and people with low levels of albuminuria (who constitute the majority of people with CKD) [18, 23, 24].

The design of EMPA-KIDNEY was finalized in 2018, before the results of CREDENCE and DAPA-CKD were known. The key objective was to assess the effect of empagliflozin on kidney disease progression in a wide range of people with CKD who were at risk of progression to ESKD, including people with or without DM and people with or without albuminuria. The rationale for testing empagliflozin in the full range of people at risk of CKD progression was based on findings from the EMPA-REG OUTCOME trial [15] and other clinical and experimental data, which showed SGLT-2 inhibitors induce glycosuria and lower BP and albuminuria [28–30]. An acute dip in eGFR on commencing an SGLT-2 inhibitor is observed, which results from the modulation of renal tubuloglomerular feedback through decreased proximal tubular sodium resorption and subsequent glomerular afferent arteriolar vasoconstriction [15, 31]. Such an effect could decrease glomerular barotrauma resulting from hyperfiltration induced by diabetes, obesity or decreased nephron number (i.e. in people with low eGFR) [32–34]. EMPA-REG OUTCOME data also raised the hypothesis—which has recently been confirmed in the EMPEROR trials [20, 21]—that empagliflozin has the potential to prevent or treat the particular types of heart disease experienced by people with CKD [8, 9, 14, 15, 35]. Further details of EMPA-KIDNEY's rationale are described in a separate publication [13]. Establishing definitive evidence of safety and efficacy in the full range of people with CKD with and without DM has particular public health importance as, worldwide, 50–70% of people with CKD do not have DM [36, 37]. In this article we describe the design, recruitment and baseline characteristics of the EMPA-KIDNEY trial and put this information in the context of other published trial data [18, 23–25].

## MATERIALS AND METHODS

### Aims

**FIGURE 1:**
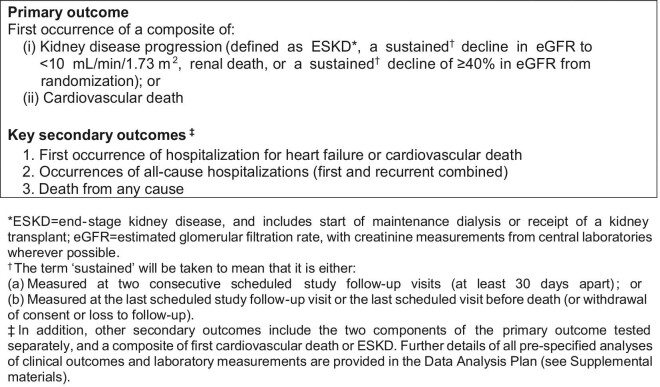
EMPA-KIDNEY key outcomes.

The EMPA-KIDNEY trial aimed to randomize ∼6000 people with pre-existing CKD to empagliflozin 10 mg daily versus matching placebo in order to assess empagliflozin's effect on time to the primary composite outcome of kidney disease progression or cardiovascular death (Figure 1). Kidney disease progression is defined as ESKD, renal death, a sustained decline in eGFR to <10 mL/min/1.73 m^2^ or a ≥40% eGFR decline from randomization. Key secondary aims are to assess the effect of empagliflozin on time to hospitalization for heart failure or cardiovascular death, occurrences of hospitalizations from any cause and time to death from any cause. Other assessments include analyses of safety and biochemical effects.

EMPA-KIDNEY's design is streamlined, with work for collaborating sites kept to a minimum and only essential information collected. The trial focusses on readily identifiable and important clinical outcomes and data collection relies mainly on participant-reported information recorded at interviews directly into a bespoke web-based system and centrally measured serum creatinine.

### Trial organization

EMPA-KIDNEY was designed and led scientifically by a Steering Committee, which is constituted from an Executive Committee, plus national representatives from each recruiting region, with additional clinical and statistical experts. The Steering Committee's responsibilities include reviewing trial progress and new scientific evidence that may be of relevance and suggesting/agreeing to changes to the protocol (provided in the Supplementary materials); agreeing to the pre-specified Data Analysis Plan (DAP, also provided in the Supplementary materials); drafting, reviewing and approving the trial's main publications (including this manuscript); reviewing and approving proposals for substudies, subsequent analyses and publications; and reviewing the trial's quality and risk management approaches. These approaches were based on the principles of Quality-by-Design [38], which aims to prospectively build quality into the study design and operations rather than relying on retrospective monitoring and focusses on those factors that are critical to quality (i.e. the protection of the participants and reliability of the trial results). Responsibility for regulatory submissions and interactions and for oversight of the trial remain with Boehringer Ingelheim—the regulatory sponsor and manufacturer of empagliflozin. Boehringer Ingelheim and Eli Lilly have provided funding for EMPA-KIDNEY in a grant to the University of Oxford (where the EMPA-KIDNEY Central Coordinating Office is based).

An independent DMC is responsible for reviewing interim unblinded analyses to assess participant safety and trial progress and for making recommendations to the Steering Committee and sponsor on whether to continue, modify or stop the trial. The DMC reviews analyses typically every 6–12 months, depending on the stage of the trial, with a chairman's review in the intervening period, when necessary. The DMC is also responsible for a formal interim efficacy analysis after 150 participants have experienced an ESKD event [interim analysis rules are defined in the protocol (see Supplementary materials available online)].

### Eligibility

**FIGURE 2:**
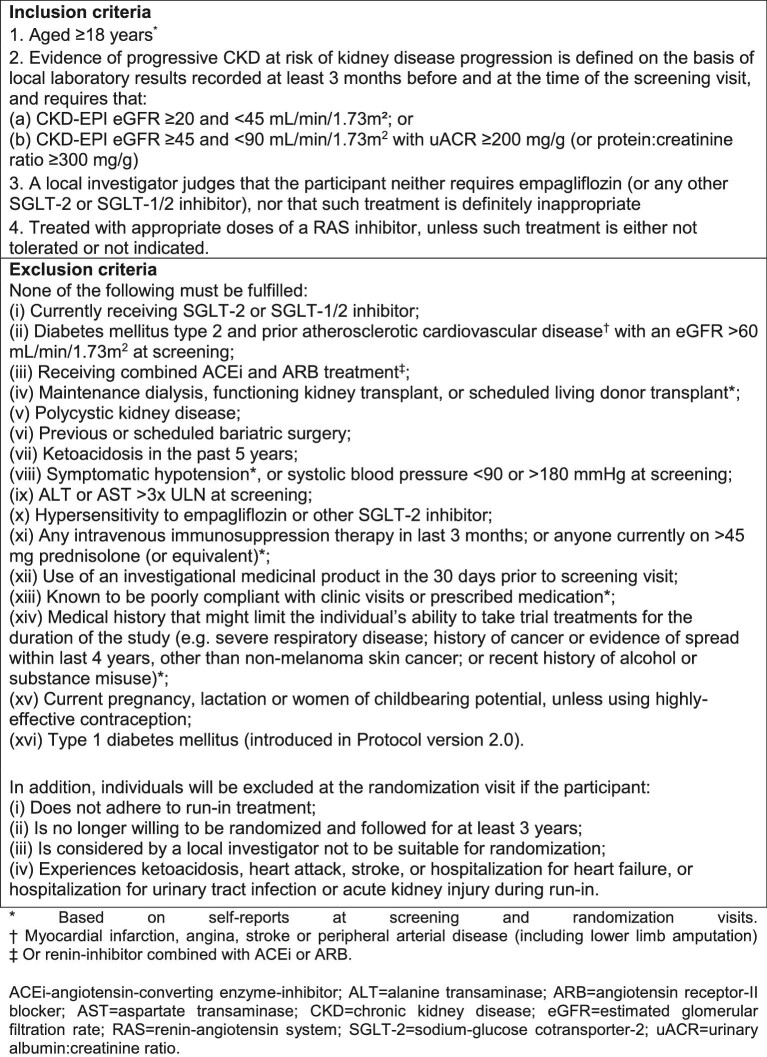
Inclusion and exclusion criteria for entry into the EMPA-KIDNEY trial.

Adults ≥18 years of age were eligible, provided a local investigator judged that they neither required an SGLT-2 inhibitor nor that such treatment was inappropriate. Participants were also required to have CKD and to be at risk of progressive disease. This was established using local laboratory results from samples taken both ≥3 months before and at the time of the screening visit and defined as either an eGFR ≥20 but <45 mL/min/1.73 m^2^  or an eGFR ≥45 but <90 mL/min/1.73 m^2^ with a uACR ≥200 mg/g. All trial eGFR values are estimated using the Chronic Kidney Disease Epidemiology Collaboration creatinine formula available at the start of recruitment (i.e. adjustment for race was included) [39]. People with or without DM were eligible, although it was pre-specified that the trial should recruit at least one-third of its sample from each patient group. Participants were also required to be prescribed a clinically appropriate dose of single-agent RAS inhibitor, unless such treatment was either not tolerated or not indicated (as judged by a local investigator). Figure 2 provides all eligibility criteria. The only excluded primary renal diagnosis was polycystic kidney disease. The initial approved protocol (V1.4) excluded participants on immunosuppression or >10 mg prednisolone. Protocol V2.0 was implemented on 21 May 2021 and removed this exclusion unless they were on prednisolone >45 mg or had received intravenous immunosuppression in the last 3 months. Following a request from the sponsor, Protocol V2.0 also further excluded people with type 1 DM (i.e. no safety concern had been reported by the DMC).

### Invitation, pre-screening, screening, run-in and randomization

Regulatory authorities and relevant research ethics committees/institutional review boards approved the protocol in each participating region. Extensive pre-screening efforts were undertaken to identify large numbers of potential participants at each site before proceeding with local recruitment. The exact methods varied by region, but the aim was to identify potentially eligible participants (based on age and laboratory results) from clinical records (including electronic healthcare records) and to contact potential participants to seek their provisional agreement to attend a screening visit.

At the screening visit, trained local research staff rechecked historical local laboratory results against inclusion criteria and obtained written informed consent. Participants were then interviewed to collect relevant medical history, non-study medication and other factors pertinent to eligibility, rather than performing a hospital records review. The participants underwent BP measurement and provided blood and urine samples for local analyses to determine eligibility. All these data were recorded directly on the trial's bespoke web-based system. Eligible participants were then provided with a 15-week supply of once-daily single-blind placebo and an appointment was made for a randomization visit in 8–12 weeks. A study treatment information leaflet explaining the need to temporarily stop study treatment if unable to eat (e.g. if unwell or preparing for a medical procedure) was provided to all participants. Those with DM were provided with a detailed information leaflet about how to recognize and minimize the risk of ketoacidosis. Those with type 1 DM were also required to possess a ketone meter and received guidance on when to use it and appropriate actions to take.

A key objective of this single-blind pre-randomization run-in was to help identify, and exclude before randomization, those individuals who would be unlikely to comply with long-term study treatment and follow-up. During this run-in, local investigators were provided with the screening visit data. They were asked to assess whether the participant required an SGLT-2 inhibitor or not. Local investigators were also asked to indicate if, in their opinion, an appropriate dose of single-agent RAS inhibitor was prescribed (i.e. the standard of care was assessed). Those who were considered to need to start a RAS inhibitor or were on an inappropriate dose were dropped out of the run-in and offered the opportunity to be rescreened once established on appropriate treatment. Local investigators were also reminded that throughout the trial, responsibility for ensuring appropriate and individualized care—including whether study treatment should be modified if dialysis is ever required—remained with the participants’ local doctors. This included appropriate management of the risk of kidney disease progression, cardiovascular diseases and other conditions that are common in CKD, according to prevailing guidelines. Local investigators also reviewed and, where necessary, updated the participant-reported primary renal diagnosis.

Participants attended a randomization visit 8–12 weeks after entering the run-in, at which it was ascertained whether any serious adverse events or significant problems had been encountered during the run-in and whether they remained willing to take study medication and attend follow-up visits for at least 3 years. Certain exclusion criteria were also rechecked (Figure 2). Information on other relevant factors was then collected, including prior diagnosis of urosepsis, heart failure, peripheral neuropathy, diabetic foot ulcer, lower limb infection or gangrene, smoking history and alcohol intake and an assessment of health-related quality of life using the European Quality of Life 5-Dimensions 5-Level (EQ5D-5L) questionnaire. BP, height, weight and hip and waist circumference were measured and a blood sample for the local measurement of creatinine, potassium, liver function and haemoglobin/haematocrit was requested. Central blood and urine samples were also collected, processed and frozen in preparation for subsequent transportation for a central assay of serum creatinine, N-terminal pro-B-type natriuretic peptide and uACR. Eligible participants were then randomized using a minimization algorithm to ensure balance for important prognostic variables (see protocol for details) [40]. Participants were provided with a 7-month supply of either active empagliflozin 10 mg once daily or matching placebo.

### Follow-up

Follow-up visits were scheduled at 2 and 6 months and then every 6 months until the end of the trial. At each follow-up visit, details of any renal replacement therapy and all serious adverse events are sought from participants, with questions specifically seeking information on the following serious adverse events: urinary tract infection, genital infection, hyperkalaemia, acute kidney injury and dehydration. Information on the following events, whether considered to be serious or not, is sought specifically: new onset of diabetes, gout, adverse events of special interest (i.e. liver injury, ketoacidosis and lower limb amputation), bone fractures, severe hypoglycaemia and symptomatic dehydration. Adherence to study treatment is assessed by asking participants about missed doses and visual inspection of remaining tablets (i.e. pill counts are not performed). The reason for discontinuation of study treatment is recorded. BP and weight measurements are requested at each in-person follow-up visit. At the 18-month and final follow-up visits, remeasurement of hip and waist circumference is requested and EQ5D-5L questionnaires are administered. Where appropriate, a further 7-month supply of study treatment is issued.

Local analysis of blood samples is used to monitor safety, including the measurement of creatinine, potassium and liver function. Central blood samples for serum creatinine and long-term storage (where permissions allow) are requested at every attended scheduled follow-up visit. Urine samples for central analysis of uACR are also requested at 2 and 18 months and at final follow-up visits. Local analysis for sodium, calcium, phosphate, haemoglobin/haematocrit and post-trial creatinine and uACR is also planned in a subset of ∼20% of participants.

Follow-up information is requested from all study participants, irrespective of whether they continue to take study treatment, unless they withdraw such consent. For participants who become unwilling or unable to attend study clinic visits, local research staff telephone the participant (or interview their local doctor or relative). All efforts are made to continue to follow up such participants, with those being followed remotely encouraged to continue to provide central blood samples.

### Adaptations due to coronavirus disease 2019 (COVID-19)

In early to mid-2020, screening into EMPA-KIDNEY was temporarily halted in regions/sites particularly affected by the COVID-19 pandemic and the run-in window was temporarily extended to 6–15 weeks where required. Those participants who were unable to attend their randomization visit could be offered a rescreening appointment. Follow-up of randomized participants unable to attend the study clinic remained possible via telephone or other remote methods (as detailed above) and study treatment could be delivered by courier. Central sample collection was requested once clinic visits were able to restart, and retrospective entries of any local measurements of creatinine were sought where central samples were missing.

### Statistical power and outcomes

EMPA-KIDNEY is event-driven, with follow-up planned until at least 1070 participants have experienced a first primary outcome (Figure 1). This number of outcomes provides 90% power at a two-sided *P*-value of 0.05 to detect an 18% relative risk reduction for the primary outcome. Extra eGFR measurements made outside of scheduled follow-up visits (e.g. triggered by a certain percentage decline in eGFR) are not requested, to minimize participant burden, thereby helping with long-term adherence to the follow-up schedule. Extra contact with participants between scheduled visits could also introduce differences in follow-up between treatment arms. To avoid introducing such bias, the term ‘sustained’ is defined as either measured at two consecutive scheduled study follow-up visits or measured at the last scheduled study follow-up visit or the last scheduled visit before death (or withdrawal of consent) (see the DAP for more details of how biases due to extra eGFR measurements are avoided). ESKD is defined as receipt of a kidney transplant or initiation of maintenance dialysis (i.e. duration ≥90 days). For ESKD, no local medical notes are collected for central review by a panel of blinded clinicians (i.e. no adjudication), but all initiations of dialysis and other changes to renal status (e.g. recovery from any dialysis requirement or transplantation) are subject to verification by local investigators. Outcomes based purely on laboratory values are also not subject to adjudication. eGFR-based analyses use measurements made at central laboratories, with local results substituted if central results are unavailable. The following outcomes are subject to clinical adjudication: all deaths and events initially reported as hospitalization for heart failure, myocardial infarction, stroke, liver injury, ketoacidosis, lower limb amputation, acute kidney injury and serious genital infections.

## RESULTS

### Recruitment

**FIGURE 3:**
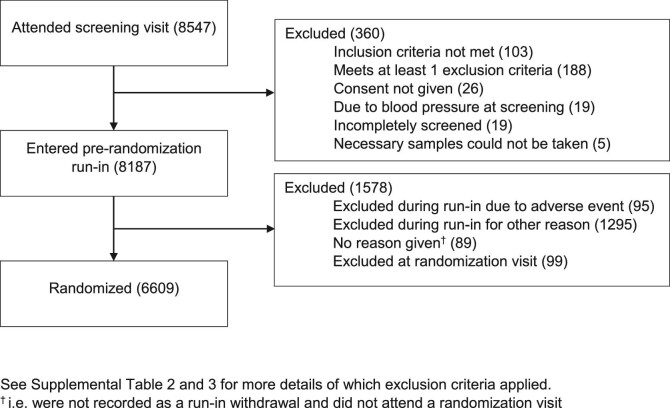
Trial profile—flow of participants through EMPA-KIDNEY trial recruitment.

A total of 8547 potential participants attended a screening visit at 241 sites across eight contributing countries (Supplementary data, Table S1). Of these screened participants, 8187 (96%) entered the pre-randomization run-in and, between 15 May 2019 and 16 April 2021, 6609 were randomized (Figure 3). The temporary stop to screening visits in regions particularly affected by COVID-19 led to a bimodal pattern of weekly randomizations (Supplementary data, Figure S1).

Of the 360 who did not enter the run-in, 103 (29%) did not meet an inclusion criterion, 188 (52%) met at least one exclusion criterion, 26 (7%) did not provide written consent, 19 (5%) had ineligible BP readings and 24 (7%) were excluded for other reasons (Supplementary data, Table S2). Of the 1479 who dropped out during the run-in period, 19 died (1%), 47 (3%) had a non-fatal serious adverse event, 29 (2%) had a non-serious adverse event and 1295 (88%) dropped out for another reason with laboratory results from the screening visit indicating ineligibility being the most common reason and 89 (6%) did not provide a reason for not attending their randomization visit (Supplementary data, Table S3a). A total of 6708 participants attended a randomization visit, of which 6609 were randomized (Supplementary data, Table S3b and Figure 3).

### Characteristics of randomized participants

Of the 6609 randomized participants, 2648 (40%) were from Europe, 1717 (26%) from North America, 1632 (25%) from China and Malaysia and 612 (9%) from Japan (Table 1 and Supplementary data, Table S4). A total of 3859 (58%) participants identified as White, 2393 (36%) as Asian, 262 (4%) as Black and 95 (1%) as mixed or other race. (Of the 1229 recruited in the USA, 17% identified as Black and 17% as Hispanic or Latino). The mean age was 63.8 years [standard deviation (SD) 13.9] and 2192 (33%) were women. There were 3039 (46%) people with DM, including 69 participants with type 1 DM. Those with DM were on average older than the 3570 (54%) participants without DM (68.6 versus 59.8 years) and more likely to report a history of cardiovascular disease [1104/3039 (36%) versus 661/3570 (19%)] (Table 1).

**Table 1.** Baseline characteristics of randomized participants, by diabetes status

		DM	
	Overall	Yes	No
Characteristics	(*N* = 6609)	(*n* = 3039)	(*n* = 3570)
Demographics			
Age at randomization (years)			
Mean (SD)	63.8 (13.9)	68.6 (9.9)	59.8 (15.4)
<60	2252 (34)	545 (18)	1707 (48)
≥60–<70	1720 (26)	969 (32)	751 (21)
≥70	2637 (40)	1525 (50)	1112 (31)
Sex			
Male	4417 (67)	2044 (67)	2373 (66)
Female	2192 (33)	995 (33)	1197 (34)
Region			
Europe (UK, Germany, Italy)	2648 (40)	1050 (35)	1598 (45)
North America (USA, Canada)	1717 (26)	1066 (35)	651 (18)
China, Malaysia	1632 (25)	632 (21)	1000 (28)
Japan	612 (9)	291 (10)	321 (9)
Race (all regions)			
White	3859 (58)	1809 (60)	2050 (57)
Black	262 (4)	173 (6)	89 (2)
Asian	2393 (36)	1008 (33)	1385 (39)
Mixed	21 (<1)	10 (<1)	11 (<1)
Other	74 (1)	39 (1)	35 (1)
Prior disease			
Prior DM			
Yes	3039 (46)	3039 (100)	
No	3570 (54)		3570 (100)
Prior DM type			
Type 1	69 (1)	69 (2)	
Type 2	2934 (44)	2934 (97)	
Other/unknown	36 (1)	36 (1)	
History of cardiovascular disease^a^			
Yes	1765 (27)	1104 (36)	661 (19)
No	4844 (73)	1935 (64)	2909 (81)
History of heart failure			
Yes	658 (10)	431 (14)	227 (6)
No or missing	5951 (90)	2608 (86)	3343 (94)
History of peripheral arterial disease			
Yes	470 (7)	318 (10)	152 (4)
No	6139 (93)	2721 (90)	3418 (96)
Clinical measurements			
Systolic BP (mmHg)			
Mean (SD)	136.5 (18.3)	139.2 (18.8)	134.3 (17.5)
<130	2398 (36)	960 (32)	1438 (40)
≥130–<145	2189 (33)	958 (32)	1231 (34)
≥145	2022 (31)	1121 (37)	901 (25)
Diastolic BP (mmHg)			
Mean (SD)	78.1 (11.8)	75.5 (11.4)	80.2 (11.7)
<75	2580 (39)	1471 (48)	1109 (31)
≥75–<85	2052 (31)	873 (29)	1179 (33)
≥85	1977 (30)	695 (23)	1282 (36)
Body mass index (kg/m^2^)			
Mean (SD)	29.7 (6.8)	31.8 (7.1)	28.0 (5.9)
<25	1620 (25)	464 (15)	1156 (32)
≥25–<30	2296 (35)	929 (31)	1367 (38)
≥30	2677 (41)	1636 (54)	1041 (29)
Missing	16 (<1)	10 (<1)	6 (<1)
Laboratory measurements			
Glycosylated haemoglobin (mmol/mol)			
Mean (SD)	45.0 (13.6)	54.9 (14.3)	36.6 (4.0)
<39	2683 (41)	209 (7)	2474 (69)
≥39–<48	1837 (28)	805 (26)	1032 (29)
≥48	1978 (30)	1978 (65)	
Missing	111 (2)	47 (2)	64 (2)
<75		2740 (90)	
≥75		252 (8)	
NT-proBNP (ng/L)			
Median (IQR)	190.3 (93.5–477.7)	238.3 (106.7–604.0)	163.3 (83.9–388.9)
<110	2391 (36)	899 (30)	1492 (42)
≥110–<330	2061 (31)	967 (32)	1094 (31)
≥330	1979 (30)	1119 (37)	860 (24)
Missing	178 (3)	54 (2)	124 (3)
Haematocrit (%)			
Mean (SD)	39.1 (5.1)	38.4 (5.1)	39.7 (5.1)
<37%	1818 (28)	938 (31)	880 (25)
≥37–<41%	1888 (29)	878 (29)	1010 (28)
≥41%	2252 (34)	843 (28)	1409 (39)
Missing	651 (10)	380 (13)	271 (8)
eGFR (mL/min/1.73 m^2^)^b^			
Mean (SD)	37.5 (14.8)	36.0 (13.9)	38.7 (15.4)
<30	2280 (34)	1148 (38)	1132 (32)
≥30–<45	2905 (44)	1359 (45)	1546 (43)
≥45	1424 (22)	532 (18)	892 (25)
uACR (mg/g)^b^			
Median (IQR)	412 (94–1190)	348 (68–1293)	461 (128–1117)
<30	1332 (20)	649 (21)	683 (19)
≥30–<300	1862 (28)	941 (31)	921 (26)
≥300	3415 (52)	1449 (48)	1966 (55)
KDIGO risk category			
Low, moderate or high	1698 (26)	730 (24)	968 (27)
Very high	4911 (74)	2309 (76)	2602 (73)
RAS inhibitor use			
uACR <200 mg/g			
No RAS inhibitor	510 (19)	217 (16)	293 (22)
RAS inhibitor	2214 (81)	1179 (84)	1035 (78)
uACR ≥200 mg/g			
No RAS inhibitor	486 (13)	237 (14)	249 (11)
RAS inhibitor	3399 (87)	1406 (86)	1993 (89)

Values are presented as *n* (%) unless stated otherwise.

a Defined as a history of myocardial infarction, heart failure, stroke, transient ischaemic attack or peripheral arterial disease.

b Uses central measurement taken at the randomization visit or most recent local laboratory result before randomization.

Prior DM is defined as a participant-reported history of diabetes of any type, use of glucose-lowering medication or baseline HbA1c ≥48 mmol/mol at the randomization visit.

NT-proBNP, N-terminal pro B-type natriuretic peptide.

The mean eGFR at randomization was 37.5 mL/min/1.73 m^2^ (SD 14.8), slightly lower in people with DM compared with those without DM [36.0 (13.9) versus 38.7 (15.4) mL/min/1.73 m^2^]. A total of 2280 (34%) had an eGFR <30 mL/min/1.73 m^2^,  2905 (44%) had an eGFR ≥30 but <45  and 1424 (22%) had an eGFR ≥45 (Table 1).

The median uACR was 412 mg/g [quartile 1–quartile 3 (Q1–Q3) 94–1190], with about half [3194/6609 (48%)] with a uACR <300 mg/g. The median uACR was 586 mg/g (Q1–Q3 256–1272) in those with an eGFR ≥45 mL/min/1.73 m^2^ and 354 (69–1154) in those with an eGFR <45. The uACR was slightly lower in people with DM than in those without DM [348 mg/g (Q1–Q3 68–1293) versus 461 (128–1117)].

In people with DM, ∼85% were using a RAS inhibitor at baseline, irrespective of the level of albuminuria. Among those without DM, 89% of those with uACR ≥200 mg/g were prescribed a RAS inhibitor, compared with 78% with a uACR <200 mg/g. Investigator-reviewed participant-reported reasons for not taking a RAS inhibitor at randomization (*n* = 996) were a lack of indication (e.g. negligible proteinuria) in 239 (24%), intolerance in 277 (28%), other reason in 296 (30%) and unknown in 184 (18%). Supplementary data, Tables S5 and S6 provide details of other medication use at randomization by diabetes status and region, respectively.

Overall, 2057 (31%) participants were considered by local investigators to have a primary renal diagnosis of diabetic kidney disease, 1669 (25%) had a glomerular disease, 1445 (22%) had hypertensive/renovascular disease and 808 (12%) had another known kidney disease, leaving 630 (10%) ascribed to an unknown cause. Of the 1669 with glomerular diseases, 817 (49%) had immunoglobulin A (IgA) nephropathy [of which 782 (96%) reported a kidney biopsy) and 852 (51%) were ascribed to other types of glomerular disease. Table 2 provides further details by diabetes status, including the renal characteristics of the CREDENCE and DAPA-CKD trials [18, 23–25].

**Table 2.** Renal characteristics and primary renal diagnoses, by trial and diabetes status

	Overall	Prior DM			No prior DM	
Characteristics	EMPA-KIDNEY	EMPA-KIDNEY	DAPA-CKD	CREDENCE	EMPA-KIDNEY	DAPA-CKD
Number randomized	6609	3039	2906	4401	3570	1398
eGFR (mL/min/1.73 m^2^)						
Mean (SD)	37.5 (14.8)	36.0 (13.9)	43.8 (12.6)	56.2 (18.2)	38.7 (15.4)	41.7 (11.7)
uACR (mg/g)						
Median (IQR)	412 (94–1190)	348 (68–1293)	1017	927 (463–1833)	461 (128–1117)	861
Primary renal diagnosis						
Diabetic nephropathy/diabetic kidney disease	2057 (31)	2057 (68)	2510 (86)	4401 (100)	0 (0)	0 (0)
Hypertensive/renovascular disease	1445 (22)	401 (13)	203 (7)		1044 (29)	494 (35)
Any glomerular disease	1669 (25)	172 (6)	97 (3)		1497 (42)	598 (43)
IgA nephropathy	817 (12)	59 (2)	38 (1)		758 (21)	232 (17)
Focal segmental glomerulosclerosis	195 (3)	34 (1)	22 (1)		161 (5)	93 (7)
Membranous nephropathy	96 (1)	13 (<1)	10 (<1)		83 (2)	33 (2)
Minimal change disease	14 (<1)	4 (<1)	2 (<1)		10 (<1)	9 (1)
Other glomerular disease	547 (8)	62 (2)	25 (1)		485 (14)	231 (17)
Other	808 (12)	203 (7)	49 (2)		605 (17)	139 (10)
Tubulointerstitial disease (including obstructive)	468 (7)	101 (3)	30 (1)		367 (10)	117 (8)
Other known	340 (5)	102 (3)	19 (1)		238 (7)	22 (2)
Unknown	630 (10)	206 (7)	47 (2)		424 (12)	167 (12)
Prior kidney biopsy	1862 (28)	354 (12)	373 (13)		1508 (42)	500 (36)

Values are presented as *n* (%) unless stated otherwise.

Sources: CREDENCE: N Engl J Med 2019;380:2295–306; DAPA-CKD: NDT 2020;35:1700–11 and Lancet Diabetes Endocrinol 2021;9:22–31.

## DISCUSSION

The EMPA-KIDNEY trial is assessing the efficacy and safety of adding empagliflozin 10 mg to the standard of care in 6609 people with CKD using a streamlined design. Its pre-screening process was relatively novel for renal trials and had multiple benefits. Advantages included a semiquantitative assessment of feasibility at each prospective site, which provided early confirmation that recruitment of 6000 participants from only eight countries was possible, and decreased the likelihood of screening failures [41, 42]. The trial's simple eligibility criteria are intended to maximize its generalizability and led to randomization of a wide range of people with CKD at risk of progression. At recruitment, slightly more than half of participants did not have DM, about three-quarters had an eGFR <45 mL/min/1.73 m^2^  and about half had levels of albuminuria <300 mg/g.

The wide range of people with CKD randomized into EMPA-KIDNEY included groups excluded from or under-represented in the other SGLT-2 inhibitor trials with a primary focus on kidney disease progression [18, 23–25]. For example, only 31% (2057/6609) of EMPA-KIDNEY participants have a local investigator-ascribed primary renal diagnosis of diabetic kidney disease, compared with 58% (2510/4304) in DAPA-CKD and all of the 4401 CREDENCE participants (Table 2). The DAPA-CKD results on non-diabetic kidney disease are based on 128 primary outcomes in 1398 participants [18]. The larger number of people without DM in EMPA-KIDNEY will provide valuable additional information on the effects of empagliflozin on cardiorenal outcomes in people without DM. We predict that ∼40–50% of primary outcomes in EMPA-KIDNEY will be among people without DM at randomization. In particular, EMPA-KIDNEY has recruited 1669 people with glomerular disease, of which 817 (49%) had IgA nephropathy and 195 (12%) had focal segmental glomerulosclerosis (90% of whom reported a kidney biopsy).

EMPA-KIDNEY includes a high proportion of participants with low levels of eGFR—a common finding among patients attending nephrology clinics (which formed the majority of its recruiting sites). Among those EMPA-KIDNEY participants with DM, the mean eGFR and median uACR at recruitment were 36.0 mL/min/1.73 m^2^ and 348 mg/g, respectively, compared with 43.8 mL/min/1.73 m^2^ and 1016.5 mg/g in DAPA-CKD and 56.2 mL/min/1.73 m^2^ and 927 mg/g in CREDENCE (Table 2). EMPA-KIDNEY will therefore help assess whether the renal benefits of SGLT-2 inhibition persist at low levels of kidney function, as suggested by CREDENCE and DAPA-CKD [18, 23, 25].

Establishing definitively whether or not albuminuria is a prerequisite for renal benefits of SGLT-2 inhibitors remains an important question, as the vast majority of people with decreased eGFR do not have albuminuria [43], and CREDENCE and DAPA-CKD provide evidence of efficacy among people with albuminuria [18, 23, 25]. If mechanistic theories that SGLT-2 inhibitors slow CKD progression by lowering intraglomerular pressure are correct [13], renal benefits may be smaller in the absence of albuminuria (a marker of intraglomerular hypertension). Nevertheless, results from meta-analyses of the earlier reported SGLT-2 inhibitor trials in type 2 DM encouragingly suggest renal benefits may extend to people without albuminuria [44], as do eGFR slope-based analyses in the trials among people with heart failure [20, 21, 45, 46]. We predict that ∼20% of EMPA-KIDNEY primary outcomes will be among people with low levels of albuminuria at recruitment.

EMPA-KIDNEY's primary outcome incorporates a ≥40% decline in eGFR from the randomization value in the trial definition of kidney disease progression, compared with the larger ≥50% decline in eGFR pre-specified by DAPA-CKD [18] and doubling of creatinine (effectively a ≥57% decline in eGFR) utilized in CREDENCE [23]. EMPA-KIDNEY employed such a definition as it aimed to recruit a large proportion of people with low levels of albuminuria and without DM who are predicted to progress more slowly than people with albuminuric diabetic CKD [47]. Smaller percentage declines in eGFR ensure such people contribute to the primary outcome. However, when testing interventions, which can cause an acute initial decline in eGFR (such as SGLT-2 inhibitors), smaller percentage declines in eGFR may be a less specific surrogate of progression to ESKD than large percentage declines [48–51]. The EMPA-KIDNEY DAP has therefore pre-specified exploratory analyses of the effects of empagliflozin versus placebo on a composite of kidney disease progression or cardiovascular death using alternative eGFR thresholds, thereby allowing some harmonization across the three trials.

## CONCLUSIONS

EMPA-KIDNEY will evaluate the renal and cardiovascular efficacy and the safety of empagliflozin in a widely generalizable population of people with CKD at risk of kidney disease progression. Results are anticipated in 2022.

## Supplementary Material

gfac040_Supplemental_File

## APPENDIX

**The EMPA-KIDNEY Collaborative Group**

*Writing Committee*

William G. Herrington, Christoph Wanner, Jennifer B. Green, Sibylle J. Hauske, Parminder Judge, Kaitlin J. Mayne, Sarah Y.A. Ng, Emily Sammons, Doreen Zhu, Natalie Staplin, David Preiss, Will Stevens, Karl Wallendszus, Rejive Dayanandan, Carol Knott, Michael Hill, Jonathan Emberson, Susanne Brenner, Vladimir Cejka, Alfred K. Cheung, Zhihong Liu, Jing Li, Peiling Chen, Laiseong Hooi, Wen Liu, Takashi Kadowaki, Masaomi Nangaku, Adeera Levin, David Cherney, Roberto Pontremoli, Aldo Pietro Maggioni, Shinya Goto, Aiko Tomita, Rajat Deo, Katherine Tuttle, Jens Eilbracht, Stefan Hantel, Mark Hopley, Martin J. Landray^†^, Colin Baigent^†^, Richard Haynes^†^ (^†^denotes joint senior authors).

*Writing Committee affiliations*

Affiliations of the Writing Committee are as follows: Clinical Trial Service Unit and Epidemiological Studies Unit, Nuffield Department of Population Health, University of Oxford, Oxford, UK (*also part of the Medical Research Council Population Health Research Unit at the University of Oxford): W.G.H.*, P.J., K.J.M., S.Y.A.N., E.S., D.Z., N.S.*, D.P.*, W.S., K.W., R.D., C.K., Mi.H.*, J.E.*, M.J.L.*, C.B.* and R.H.*; University Clinic of Würzburg, Würzburg, Germany: C.W., S.B. and V.C.; Duke Clinical Research Institute, Durham, NC, USA: J.B.G.; University of Utah, Salt Lake City, UT, USA: A.K.C.; Boehringer Ingelheim International: S.J.H., J.E., S.H. and Ma.H.; Vth Department of Medicine, University Medical Center Mannheim, University of Heidelberg, Mannheim, Germany: S.J.H.; National Clinical Research Center of Kidney Diseases, Jinling Hospital, Nanjing University School of Medicine, Nanjing, China: Z.H.L. and P.L.C.; Fuwai Hospital, Chinese Academy of Medical Sciences, National Center for Cardiovascular Diseases, Beijing, China: J.L.; Hospital Sultanah Aminah, Johor Bahru, Malaysia: L.S.H. and W.L.; University of Tokyo School of Medicine, Tokyo, Japan: T.K. and M.N.; Tokai University School of Medicine, Isehara, Japan: S.G. and A.T.; University of British Columbia, Vancouver, BC, Canada: A.L.; University of Toronto, Toronto, ON, Canada: D.C.; Università degli Studi and IRCCS Ospedale Policlinico San Martino di Genova, Genova, Italy: R.P.; ANMCO Research Center, Florence, Italy: A.M.; University of Pennsylvania Perelman School of Medicine, Philadelphia, PA, USA: R.D.; Providence Health Care and University of Washington, Washington, WA, USA: K.T.

**Members of the EMPA-KIDNEY Collaborative Group**

*Executive Committee*

Colin Baigent (Co-Chair), Martin J. Landray (Co-Chair), Christoph Wanner (Deputy Chair), William G. Herrington (Chief Investigator), Richard Haynes (Co-Principal Investigator), Jennifer B. Green, Sibylle J. Hauske*, Martina Brueckmann*, Mark Hopley* (previous members: Maximillian von-Eynatten* and Jyothis George*).

*Steering Committee*

Executive Committee members plus national representatives: Susanne Brenner (Germany); Alfred K. Cheung (USA); David Preiss (UK); Zhihong Liu, Jing Li (China); Laiseong Hooi, Wen Liu (Malaysia); Takashi Kadowaki, Masaomi Nangaku (Japan); Adeera Levin, David Cherney (Canada); Roberto Pontremoli, Aldo Pietro Maggioni (Italy); plus statistician members: Natalie Staplin, Jonathan Emberson, Stefan Hantel*; plus other expert members: Shinya Goto, Rajat Deo, Katherine Tuttle. Non-voting members: Parminder Judge, Sarah Y.A. Ng, Francisco Javier Rossello Lozano, Emily Sammons, Doreen Zhu (*denotes a Boehringer Ingelheim employee).

*Independent Data Monitoring Committee*

Peter Sandercock (Chair), Rudolf Bilous, Charles Herzog, Paul Whelton, Janet Wittes, Derrick Bennett (non-voting statistician).

*Central Coordinating Office's Senior Management Team based at the Clinical Trial Service Unit and Epidemiologial Studies Unit, Nuffield Department of Population Health, University of Oxford*

Andy Burke, Richard Brown, Rejive Dayanandan, Lucy Fletcher, Hannah Gosling, Emily Harding, Richard Haynes, William G. Herrington, Parminder Judge, Carol Knott, Ryonfa Lee, Kevin Murphy, Yanru Qiao, Rachel Raff, Hui Yu.

*Regional Coordinating Centre Administrative Leadership and Clinical Support*

YanRu Qiao (UK); Vladimir Cejka, Marcela Fajardo-Moser (Germany); Andrea Lorimer, Donata Lucci (Italy); Anita Hepditch (US); Amanda Axler (Canada); Peiling Chen, Dai Hao (China), Cheng Beng Goh, Sarojini Sivanandam (Malaysia); Akiko Hashimoto, Wakako Negoro, Aiko Tomita, Morisaki Tomoko (Japan).

*EMPA-KIDNEY Collaborators*

See Supplementary material for a full listing of collaborators.

## Notes

Members and their affiliations are provided at the end of the manuscript.

Trial registrations: Clinicaltrials.gov: NCT03594110; EuDRACT: 2017-002971-24.
