# Supplementary material for: Design, recruitment, and baseline characteristics of the EMPA-KIDNEY trial
Source: Nephrol Dial Transplant. Author manuscript; Available in PMC 2022 Jun 25. (PMC9217655; doi:10.1093/ndt/gfac040)
Supplement: Supplemental materials [file EMS143886-supplement-Supplemental_materials.pdf]

Design, recruitment, and baseline characteristics of the EMPA-KIDNEY trial

The EMPA-KIDNEY Collaborative Group

Supplemental Materials

Contents

1. Supplemental Tables and Figures ………………………………………………………… 3
   i. Supplemental Table 1: Recruitment by region and country ………………….…...... 3
   ii. Supplemental Table 2: Reasons screened patients did not enter run-in …..……. 4
   iii. Supplemental Table 3a: Reasons for withdrawing from run-in period before the randomization visit ……………………………………………………………….…. 6
   iv. Supplemental Table 3b: Reasons for withdrawing from run-in at the randomization visit ……………………………………………………………….…. 8
   v. Supplemental Table 4: Baseline characteristics of randomized participants by region ……………………………………………………………………………… 9
   vi. Supplemental Table 5: Comedication use at randomization by diabetes status …………………………………………………………………………………..… 13
   vii. Supplemental Table 6: Comedication use at randomization by region ……..… 14
   viii. Supplemental Figure 1: Randomizations by region by week ………….…...…… 15

2. Protocol ……………………………………………………………………………………16

3. Data Analysis Plan ……………………………………………………………………… 58

4. Collaborators ………………………………………………………………………… 76

5. Individual Financial Disclosures for the Writing Committee ……………………….…..… 85

Supplemental Table 1: Recruitment by region and country

	Centers*	Attended screening	Entered run-in	Attended randomization	Randomized
**Europe**	**110**	**3500**	**3328**	**2693**	**2648**
United Kingdom	53	1611	1487	1159	1133
Germany	36	1588	1546	1287	1269
Italy	21	301	295	247	246
**North America**	**68**	**2420**	**2279**	**1745**	**1717**
United States	48	1762	1636	1252	1229
Canada	20	658	643	493	488
**Asia**	**63**	**2627**	**2580**	**2270**	**2244**
China	17	1198	1189	1001	986
Malaysia	21	747	722	653	646
Japan	25	682	669	616	612
**Total**	**241**	**8547**	**8187**	**6708**	**6609**

* Count of centers that have screened at least one participant.

Supplemental Table 2: Reasons screened patients did not enter run-in

	Region									
	Overall		Europe		North America		China and Malaysia		Japan	
**Attended screening**	8547		3500		2420		1945		682	
Inclusion criteria not met	103	(1.2%)	45	(1.3%)	46	(1.9%)	6	(0.3%)	6	(0.9%)
**Fulfilled one or more exclusion criteria**										
**Medical history**										
All of the following: type 2 diabetes mellitus, prior atherosclerotic cardiovascular disease and an eGFR >60 mL/min/1.73m^2 at Screening	17	(0.2%)	3	(0.1%)	8	(0.3%)	2	(0.1%)	4	(0.6%)
Currently receiving dialysis, has kidney transplant or scheduled living transplant in the next 6 months	3	(0.0%)	2	(0.1%)	1	(0.0%)	0	(0.0%)	0	(0.0%)
Polycystic kidney disease	3	(0.0%)	2	(0.1%)	1	(0.0%)	0	(0.0%)	0	(0.0%)
Previous or planned bariatric surgery	34	(0.4%)	9	(0.3%)	25	(1.0%)	0	(0.0%)	0	(0.0%)
Ketoacidosis in last 5 years	11	(0.1%)	3	(0.1%)	7	(0.3%)	1	(0.1%)	0	(0.0%)
Postural hypotension	35	(0.4%)	20	(0.6%)	13	(0.5%)	2	(0.1%)	0	(0.0%)
Immunosuppression in last 3 months or curently taking >45mg of prednisolone	52	(0.6%)	25	(0.7%)	21	(0.9%)	5	(0.3%)	1	(0.1%)
Use of ACE inhibitor and ARB in combination	10	(0.1%)	6	(0.2%)	4	(0.2%)	0	(0.0%)	0	(0.0%)
Known poor compliance	8	(0.1%)	6	(0.2%)	1	(0.0%)	1	(0.1%)	0	(0.0%)
Other chronic medical condition that might limited individual's ability to take trial treatments for the duration of the trial	19	(0.2%)	11	(0.3%)	5	(0.2%)	3	(0.2%)	0	(0.0%)
Pregnant or breastfeeding, or unwilling to use highly effective contraception	3	(0.0%)	1	(0.0%)	0	(0.0%)	2	(0.1%)	0	(0.0%)
Type 1 diabetes mellitus	3	(0.0%)	1	(0.0%)	2	(0.1%)	0	(0.0%)	0	(0.0%)
ALT/AST >3x Upper Limit of Normal	0	(0.0%)	0	(0.0%)	0	(0.0%)	0	(0.0%)	0	(0.0%)
Any medical history reason	180	(2.1%)	81	(2.3%)	79	(3.3%)	15	(0.8%)	5	(0.7%)
**Medication history**										
Previous adverse reaction to SGLT-2 inhibitor	4	(0.0%)	1	(0.0%)	3	(0.1%)	0	(0.0%)	0	(0.0%)
Currently taking SGLT-2 inhibitor	6	(0.1%)	2	(0.1%)	3	(0.1%)	0	(0.0%)	1	(0.1%)
In a clinical trial with an unlicensed medication or device	2	(0.0%)	1	(0.0%)	0	(0.0%)	1	(0.1%)	0	(0.0%)
Any medication history reason	12	(0.1%)	4	(0.1%)	6	(0.2%)	1	(0.1%)	1	(0.1%)

(Continued)

Supplemental Table 2: Reasons screened patients did not enter run-in (continued)

	Overall		Region: Europe		North America		China and Malaysia		Japan	
**Consent**										
Consent not given	26	(0.3%)	16	(0.5%)	3	(0.1%)	7	(0.4%)	0	(0.0%)
**Blood pressure at Screening**										
Systolic BP <90 mmHg or >180 mmHg (or missing)	19	(0.2%)	7	(0.2%)	6	(0.2%)	5	(0.3%)	1	(0.1%)
**Other reason**										
Necessary blood or urine samples could not be taken	5	(0.1%)	5	(0.1%)	0	(0.0%)	0	(0.0%)	0	(0.0%)
Incomplete screening form	19	(0.2%)	16	(0.5%)	3	(0.1%)	0	(0.0%)	0	(0.0%)
Any other reason	24	(0.3%)	21	(0.6%)	3	(0.1%)	0	(0.0%)	0	(0.0%)
**Attended screening but did not enter run-in (including incomplete visit/unable to collect samples)**	360	(4.2%)	172	(4.9%)	141	(5.8%)	34	(1.7%)	13	(1.9%)
**Entered run-in**	8187	(95.8%)	3328	(95.1%)	2279	(94.2%)	1911	(98.3%)	669	(98.1%)

Participants may have more than one reason for dropping out.

Supplemental Table 3a: Reasons for withdrawing from run-in period before the randomization visit

	Overall		Europe		North America		China and Malaysia		Japan	
					Region					
**Entered run-in**	8187		3328		2279		1911		669	
**Death**										
Cardiovascular death	11	(0.1%)	3	(0.1%)	5	(0.2%)	3	(0.2%)	0	(0.0%)
Non-cardiovascular death	8	(0.1%)	1	(0.0%)	4	(0.2%)	3	(0.2%)	0	(0.0%)
Any death	19	(0.2%)	4	(0.1%)	9	(0.4%)	6	(0.3%)	0	(0.0%)
**Non-fatal serious adverse event**										
Cardiac disorders	13	(0.2%)	7	(0.2%)	4	(0.2%)	2	(0.1%)	0	(0.0%)
Neoplasms benign, malignant and unspecified (incl cysts and polyps)	8	(0.1%)	2	(0.1%)	4	(0.2%)	1	(0.1%)	1	(0.1%)
Nervous system disorders	5	(0.1%)	3	(0.1%)	1	(0.0%)	1	(0.1%)	0	(0.0%)
Renal and urinary disorders	4	(0.0%)	2	(0.1%)	2	(0.1%)	0	(0.0%)	0	(0.0%)
Surgical and medical procedures	1	(0.0%)	1	(0.0%)	0	(0.0%)	0	(0.0%)	0	(0.0%)
Other serious adverse event	16	(0.2%)	13	(0.4%)	1	(0.0%)	2	(0.1%)	0	(0.0%)
Any non-fatal SAE	47	(0.6%)	28	(0.8%)	12	(0.5%)	6	(0.3%)	1	(0.1%)
**Non-serious adverse event**										
Gastrointestinal disorders	9	(0.1%)	9	(0.3%)	0	(0.0%)	0	(0.0%)	0	(0.0%)
General disorders and administration site conditions	4	(0.0%)	2	(0.1%)	2	(0.1%)	0	(0.0%)	0	(0.0%)
Infections and infestations	1	(0.0%)	1	(0.0%)	0	(0.0%)	0	(0.0%)	0	(0.0%)
Investigations	1	(0.0%)	0	(0.0%)	1	(0.0%)	0	(0.0%)	0	(0.0%)
Musculoskeletal and connective tissue disorders	1	(0.0%)	1	(0.0%)	0	(0.0%)	0	(0.0%)	0	(0.0%)
Nervous system disorders	2	(0.0%)	0	(0.0%)	1	(0.0%)	1	(0.1%)	0	(0.0%)
Psychiatric disorders	1	(0.0%)	1	(0.0%)	0	(0.0%)	0	(0.0%)	0	(0.0%)
Renal and urinary disorders	3	(0.0%)	1	(0.0%)	1	(0.0%)	1	(0.1%)	0	(0.0%)
Skin and subcutaneous tissue disorders	4	(0.0%)	1	(0.0%)	3	(0.1%)	0	(0.0%)	0	(0.0%)
Other non-serious adverse event	3	(0.0%)	2	(0.1%)	1	(0.0%)	0	(0.0%)	0	(0.0%)
Any non-serious adverse event	29	(0.4%)	18	(0.5%)	9	(0.4%)	2	(0.1%)	0	(0.0%)
**Other reason**										
Concerns about tablets	111	(1.4%)	51	(1.5%)	19	(0.8%)	37	(1.9%)	4	(0.6%)
Results from screening visit samples do not confirm eligibility	612	(7.5%)	222	(6.7%)	245	(10.8%)	104	(5.4%)	41	(6.1%)

(Continued)

Supplemental Table 3a: Reasons for withdrawing from run-in period before the randomization visit (continued)

	Region									
	Overall		Europe		North America		China and Malaysia		Japan	
Contraindicated drug started	16	(0.2%)	11	(0.3%)	4	(0.2%)	1	(0.1%)	0	(0.0%)
Difficulty taking tablets	16	(0.2%)	4	(0.1%)	4	(0.2%)	8	(0.4%)	0	(0.0%)
Doctor advice	89	(1.1%)	24	(0.7%)	34	(1.5%)	31	(1.6%)	0	(0.0%)
Family circumstances	13	(0.2%)	7	(0.2%)	5	(0.2%)	1	(0.1%)	0	(0.0%)
Trial administration problem	167	(2.0%)	90	(2.7%)	70	(3.1%)	4	(0.2%)	3	(0.4%)
Unable to attend clinic	30	(0.4%)	11	(0.3%)	11	(0.5%)	8	(0.4%)	0	(0.0%)
Undergoing investigations	13	(0.2%)	8	(0.2%)	1	(0.0%)	4	(0.2%)	0	(0.0%)
Other reason not listed above	228	(2.8%)	96	(2.9%)	86	(3.8%)	45	(2.4%)	1	(0.1%)
Any other reason	1295	(15.8%)	524	(15.7%)	479	(21.0%)	243	(12.7%)	49	(7.3%)
**No reason given**	89	(1.1%)	61	(1.8%)	25	(1.1%)	0	(0.0%)	3	(0.4%)
**Dropped out of run-in**	1479	(18.1%)	635	(19.1%)	534	(23.4%)	257	(13.4%)	53	(7.9%)

Participants may have more than one reason for dropping out.

Supplemental Table 3b: Reasons for withdrawing from run-in at the randomization visit

	Overall		Region							
			Europe		North America		China and Malaysia		Japan	
**Attended randomization visit**	6708		2693		1745		1654		616	
**Medical history**										
Recent myocardial infarction	1	(0.0%)	1	(0.0%)	0	(0.0%)	0	(0.0%)	0	(0.0%)
Recent stroke	3	(0.0%)	1	(0.0%)	0	(0.0%)	2	(0.1%)	0	(0.0%)
Recent admission for heart failure	2	(0.0%)	2	(0.1%)	0	(0.0%)	0	(0.0%)	0	(0.0%)
Recent urinary tract infection	1	(0.0%)	1	(0.0%)	0	(0.0%)	0	(0.0%)	0	(0.0%)
Recent admission for acute kidney injury	4	(0.1%)	2	(0.1%)	1	(0.1%)	1	(0.1%)	0	(0.0%)
Adverse event attributed to run-in treatment	1	(0.0%)	0	(0.0%)	0	(0.0%)	0	(0.0%)	1	(0.2%)
Any medical history reason	11	(0.2%)	6	(0.2%)	1	(0.1%)	3	(0.2%)	1	(0.2%)
**Medication history**										
Currently taking contraindicated immunosuppression	4	(0.1%)	3	(0.1%)	1	(0.1%)	0	(0.0%)	0	(0.0%)
Any medication history reason	4	(0.1%)	3	(0.1%)	1	(0.1%)	0	(0.0%)	0	(0.0%)
**Other reason**										
Concern about long-term compliance	49	(0.7%)	15	(0.6%)	15	(0.9%)	16	(1.0%)	3	(0.5%)
Symptomatic postural hypotension	18	(0.3%)	12	(0.4%)	5	(0.3%)	1	(0.1%)	0	(0.0%)
No ketone meter available (people with type 1 diabetes)	1	(0.0%)	1	(0.0%)	0	(0.0%)	0	(0.0%)	0	(0.0%)
Other reason	16	(0.2%)	8	(0.3%)	6	(0.3%)	2	(0.1%)	0	(0.0%)
Any other reason	84	(1.3%)	36	(1.3%)	26	(1.5%)	19	(1.1%)	3	(0.5%)
**Attended randomization visit but was not randomized**	99	(1.5%)	45	(1.7%)	28	(1.6%)	22	(1.3%)	4	(0.6%)
**Randomized**	6609	(98.5%)	2648	(98.3%)	1717	(98.4%)	1632	(98.7%)	612	(99.4%)

Participants may have more than one reason for dropping out.

Supplemental Table 4: Baseline characteristics of randomized participants by region

	Overall (N=6609)		Region: Europe (N=2648)		North America (N=1717)		China and Malaysia (N=1632)		Japan (N=612)	
**DEMOGRAPHICS**										
**Age at randomization (years)**										
Mean (SD)	63.8	(13.9)	65.5	(14.0)	68.9	(11.0)	55.3	(13.2)	65.3	(12.0)
<60	2252	(34%)	790	(30%)	295	(17%)	982	(60%)	185	(30%)
≥60 <70	1720	(26%)	646	(24%)	495	(29%)	412	(25%)	167	(27%)
≥70	2637	(40%)	1212	(46%)	927	(54%)	238	(15%)	260	(42%)
**Sex**										
Male	4417	(67%)	1874	(71%)	1025	(60%)	1067	(65%)	451	(74%)
Female	2192	(33%)	774	(29%)	692	(40%)	565	(35%)	161	(26%)
**Race**										
White	3859	(58%)	2497	(94%)	1362	(79%)				
Black	262	(4%)	39	(1%)	223	(13%)				
Asian	2393	(36%)	76	(3%)	75	(4%)	1630	(100%)	612	(100%)
Mixed	21	(0%)	12	(0%)	9	(1%)				
Other	74	(1%)	24	(1%)	48	(3%)	2	(0%)		
**PRIOR DISEASE**										
**Prior diabetes mellitus**										
Yes	3039	(46%)	1050	(40%)	1066	(62%)	632	(39%)	291	(48%)
No	3570	(54%)	1598	(60%)	651	(38%)	1000	(61%)	321	(52%)
**Prior diabetes mellitus type**										
Type 1	69	(1%)	34	(1%)	34	(2%)	1	(0%)		
Type 2	2934	(44%)	1008	(38%)	1028	(60%)	622	(38%)	276	(45%)
Other/unknown	36	(1%)	8	(0%)	4	(0%)	9	(1%)	15	(2%)
**History of cardiovascular disease***										
Yes	1765	(27%)	894	(34%)	543	(32%)	238	(15%)	90	(15%)
No	4844	(73%)	1754	(66%)	1174	(68%)	1394	(85%)	522	(85%)

(Continued)

Supplemental Table 4: Baseline characteristics of randomized participants by region (continued)

	Overall (N=6609)		Europe (N=2648)		North America (N=1717)		China and Malaysia (N=1632)		Japan (N=612)	
			Region							
**History of heart failure**										
Yes	658	(10%)	392	(15%)	198	(12%)	39	(2%)	29	(5%)
No or missing	5951	(90%)	2256	(85%)	1519	(88%)	1593	(98%)	583	(95%)
**History of peripheral arterial disease**										
Yes	470	(7%)	264	(10%)	166	(10%)	27	(2%)	13	(2%)
No	6139	(93%)	2384	(90%)	1551	(90%)	1605	(98%)	599	(98%)
**CLINICAL MEASUREMENTS**										
**Systolic blood pressure (mmHg)**										
Mean (SD)	136.5	(18.3)	136.7	(18.5)	133.5	(17.6)	139.9	(18.5)	134.9	(16.8)
<130	2398	(36%)	921	(35%)	747	(44%)	492	(30%)	238	(39%)
≥130 <145	2189	(33%)	932	(35%)	541	(32%)	513	(31%)	203	(33%)
≥145	2022	(31%)	795	(30%)	429	(25%)	627	(38%)	171	(28%)
**Diastolic blood pressure (mmHg)**										
Mean (SD)	78.1	(11.8)	78.2	(11.5)	73.8	(11.0)	82.4	(11.6)	78.0	(12.1)
<75	2580	(39%)	1004	(38%)	910	(53%)	403	(25%)	263	(43%)
≥75 <85	2052	(31%)	844	(32%)	529	(31%)	515	(32%)	164	(27%)
≥85	1977	(30%)	800	(30%)	278	(16%)	714	(44%)	185	(30%)
**Body mass index (kg/m^2)**										
Mean (SD)	29.7	(6.8)	30.5	(6.3)	33.1	(7.5)	26.8	(5.2)	25.2	(4.1)
<25	1620	(25%)	452	(17%)	186	(11%)	646	(40%)	336	(55%)
≥25 <30	2296	(35%)	980	(37%)	472	(27%)	642	(39%)	202	(33%)
≥30	2677	(41%)	1208	(46%)	1051	(61%)	344	(21%)	74	(12%)
Missing	16	(0%)	8	(0%)	8	(0%)				

(Continued)

Supplemental Table 4: Baseline characteristics of randomized participants by region (continued)

	Overall (N=6609)		Region: Europe (N=2648)		North America (N=1717)		China and Malaysia (N=1632)		Japan (N=612)	
**LABORATORY MEASUREMENTS**										
**NT-proBNP (ng/L)**										
Median (IQR)	190.3	(93.5-477.7)	228.3	(109.9-613.3)	233.6	(100.9-537.2)	137.0	(84.3-298.6)	125.1	(65.2-268.6)
<110	2391	(36%)	746	(28%)	510	(30%)	828	(51%)	307	(50%)
≥110 <330	2061	(31%)	872	(33%)	534	(31%)	484	(30%)	171	(28%)
≥330	1979	(30%)	971	(37%)	614	(36%)	289	(18%)	105	(17%)
Missing	178	(3%)	59	(2%)	59	(3%)	31	(2%)	29	(5%)
**Haematocrit (%)**										
Mean (SD)	39.1	(5.1)	39.2	(4.9)	38.7	(5.1)	38.8	(5.4)	40.1	(5.1)
<37%	1818	(28%)	689	(26%)	434	(25%)	551	(34%)	144	(24%)
≥37% <41%	1888	(29%)	782	(30%)	453	(26%)	463	(28%)	190	(31%)
≥41%	2252	(34%)	923	(35%)	462	(27%)	595	(36%)	272	(44%)
Missing	651	(10%)	254	(10%)	368	(21%)	23	(1%)	6	(1%)
**eGFR (mL/min/1.73m²)**†										
Mean (SD)	37.5	(14.8)	35.0	(12.2)	35.1	(11.2)	41.1	(18.4)	45.2	(18.2)
<30	2280	(34%)	1029	(39%)	602	(35%)	515	(32%)	134	(22%)
≥30 <45	2905	(44%)	1205	(46%)	867	(50%)	616	(38%)	217	(35%)
≥45	1424	(22%)	414	(16%)	248	(14%)	501	(31%)	261	(43%)
**UACR (mg/g)**†										
Median (IQR)	412	(94-1190)	342	(63-1092)	210	(45-782)	656	(246-1490)	690	(295-1596)
<30	1332	(20%)	656	(25%)	522	(30%)	127	(8%)	27	(4%)
≥30 <300	1862	(28%)	769	(29%)	570	(33%)	390	(24%)	133	(22%)
≥300	3415	(52%)	1223	(46%)	625	(36%)	1115	(68%)	452	(74%)

(Continued)

Supplemental Table 4: Baseline characteristics of randomized participants by region (continued)

	Overall (N=6609)		Europe (N=2648)		North America (N=1717)		China and Malaysia (N=1632)		Japan (N=612)	
**KDIGO risk category**										
Low, moderate or high	1698	(26%)	629	(24%)	504	(29%)	383	(23%)	182	(30%)
Very high	4911	(74%)	2019	(76%)	1213	(71%)	1249	(77%)	430	(70%)
**RAS INHIBITOR USE**										
**UACR <200mg/g**										
No RAS inhibitor	510	(19%)	215	(17%)	230	(24%)	44	(12%)	21	(20%)
RAS inhibitor	2214	(81%)	1047	(83%)	743	(76%)	338	(88%)	86	(80%)
**UACR ≥200mg/g**										
No RAS inhibitor	486	(13%)	142	(10%)	113	(15%)	169	(14%)	62	(12%)
RAS inhibitor	3399	(87%)	1244	(90%)	631	(85%)	1081	(86%)	443	(88%)

Region columns: Europe, North America, China and Malaysia, Japan.

Figures are n (%) or mean (SD).
[*] Defined as self-reported history of myocardial infarction, heart failure, stroke, transient ischemic attack or peripheral arterial disease.
[†] Uses central measurement taken at the randomization visit, or most recent local laboratory result before randomization.
Prior diabetes mellitus defined as participant-reported history of diabetes of any type, use of glucose-lowering medication or baseline HbA1c ≥48 mmol/mol at randomization.
Abbreviations: NT-proBNP= N-terminal pro B-type natriuretic peptide; eGFR=estimated glomerular filtration rate; uACR=urinary albumin:creatinine ratio; KDIGO=Kidney Disease: Improving Global Outcomes; RAS=renin-angiotensin system.

Supplemental Table 5: Comedication use at randomization by diabetes status

	Overall (N=6609)		Diabetes mellitus Yes (N=3039)		No (N=3570)	
RAS inhibitor	5613	(85%)	2585	(85%)	3028	(85%)
Any diuretic	2776	(42%)	1641	(54%)	1135	(32%)
Loop diuretic	1727	(26%)	1099	(36%)	628	(18%)
Thiazide diuretic	1085	(16%)	611	(20%)	474	(13%)
Mineralocorticoid receptor antagonist	473	(7%)	256	(8%)	217	(6%)
Potassium sparing & other	36	(1%)	21	(1%)	15	(0%)
Beta blocker	2759	(42%)	1570	(52%)	1189	(33%)
Any lipid-lowering medication	4376	(66%)	2496	(82%)	1880	(53%)
Anticoagulant	315	(5%)	175	(6%)	140	(4%)
Antiplatelet therapy	2238	(34%)	1446	(48%)	792	(22%)
Biguanide (e.g. metformin)	668	(10%)	668	(22%)		
Sulphonylurea	583	(9%)	583	(19%)		
Insulin	1663	(25%)	1663	(55%)		
DPP-4 inhibitor	792	(12%)	792	(26%)		
GLP-1 agonist	315	(5%)	315	(10%)		
Other antidiabetic drug	313	(5%)	313	(10%)		

Figures are n (%).
Abbreviations: RAS=renin-angiotensin system; DPP-4=dipeptidyl peptidase-4; GLP-1=glucagon-like peptide-1.

Supplemental Table 6: Comedication use at randomization by region

	Overall (N=6609)		Region: Europe (N=2648)		North America (N=1717)		China and Malaysia (N=1632)		Japan (N=612)	
RAS inhibitor	5613	(85%)	2291	(87%)	1374	(80%)	1419	(87%)	529	(86%)
Any diuretic	2776	(42%)	1378	(52%)	943	(55%)	342	(21%)	113	(18%)
Loop diuretic	1727	(26%)	964	(36%)	554	(32%)	155	(9%)	54	(9%)
Thiazide diuretic	1085	(16%)	476	(18%)	414	(24%)	146	(9%)	49	(8%)
Mineralocorticoid receptor antagonist	473	(7%)	258	(10%)	121	(7%)	70	(4%)	24	(4%)
Potassium sparing & other	36	(1%)	11	(0%)	20	(1%)	4	(0%)	1	(0%)
Beta blocker	2759	(42%)	1255	(47%)	876	(51%)	528	(32%)	100	(16%)
Any lipid-lowering medication	4376	(66%)	1817	(69%)	1352	(79%)	834	(51%)	373	(61%)
Anticoagulant	315	(5%)	193	(7%)	89	(5%)	12	(1%)	21	(3%)
Antiplatelet therapy	2238	(34%)	880	(33%)	793	(46%)	407	(25%)	158	(26%)
Biguanide (e.g. metformin)	668	(10%)	248	(9%)	207	(12%)	142	(9%)	71	(12%)
Sulphonylurea	583	(9%)	119	(4%)	232	(14%)	163	(10%)	69	(11%)
Insulin	1663	(25%)	554	(21%)	635	(37%)	362	(22%)	112	(18%)
DPP-4 inhibitor	792	(12%)	324	(12%)	254	(15%)	80	(5%)	134	(22%)
GLP-1 agonist	315	(5%)	101	(4%)	169	(10%)	13	(1%)	32	(5%)
Other antidiabetic drug	313	(5%)	47	(2%)	74	(4%)	114	(7%)	78	(13%)

Figures are n (%).
Abbreviations: RAS=renin-angiotensin system; DPP-4=dipeptidyl peptidase-4; GLP-1=glucagon-like peptide-1.

Supplemental Figure 1: Randomizations by region by week

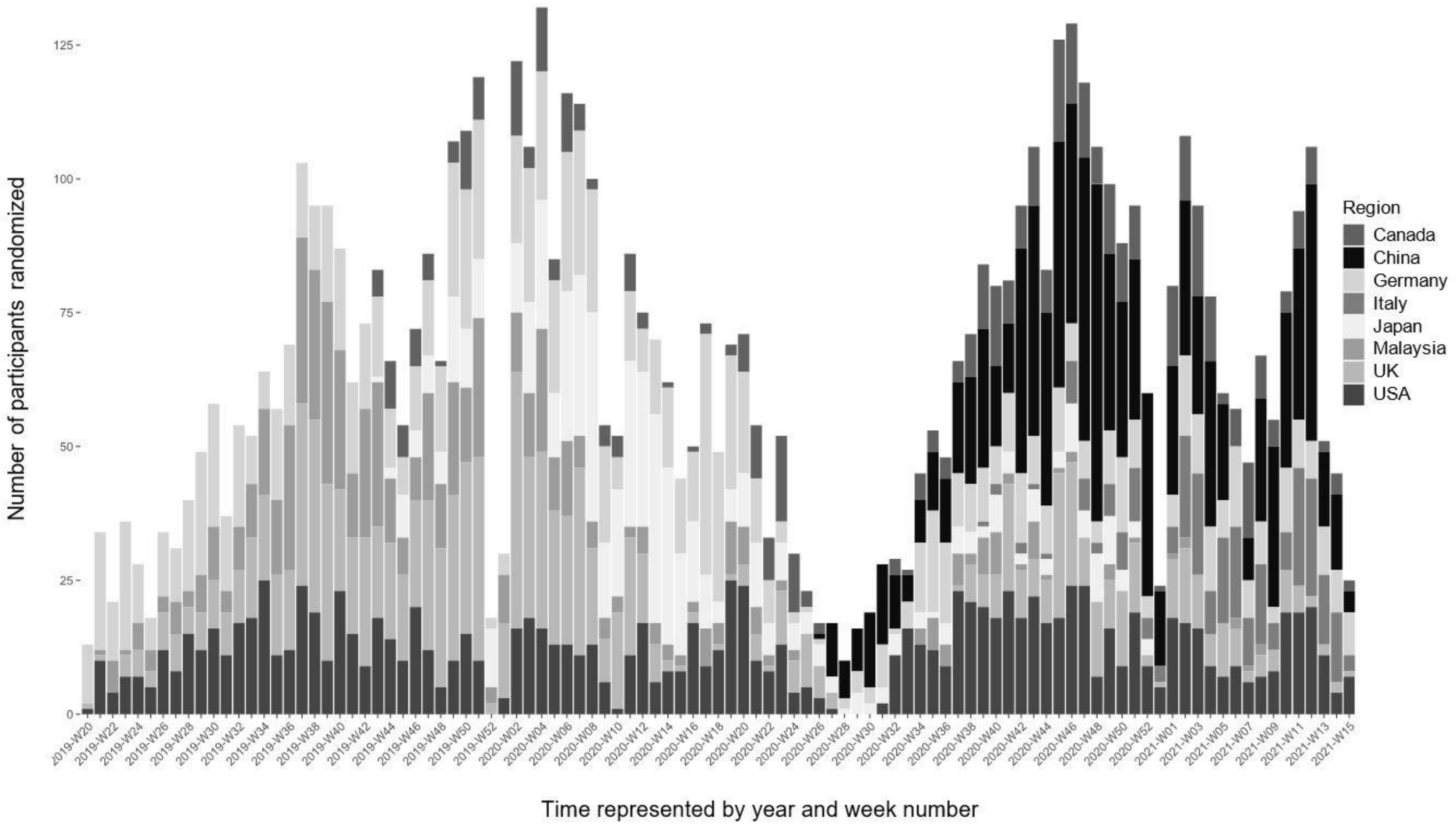

EMPA-KIDNEY Trial Protocol

A multicentre international randomized parallel group double-blind placebo-controlled clinical trial of EMPAgliflozin once daily to assess cardio-renal outcomes in patients with chronic KIDNEY disease

Does inhibition of sodium-glucose co-transporter-2 with empagliflozin prevent kidney disease progression and cardiovascular death in patients with chronic kidney disease?

Selective inhibition of sodium-glucose co-transporter-2 (SGLT-2) with empagliflozin causes urinary glucose excretion and reduces hyperglycaemia, weight, plasma circulating volume and blood pressure. This has been shown to translate safely into reduced clinical risk from cardiovascular disease (particularly heart failure and cardiovascular death) in people with type 2 diabetes (T2D) and established cardiovascular disease. SGLT-2 inhibition with empagliflozin also reduces albuminuria and slows the annual decline in estimated glomerular filtration rate in people with T2D who still have preserved kidney function. The kidney effects may result from increased sodium delivery to the kidney’s macula densa, which in turn causes glomerular afferent arteriolar vasoconstriction and reduced intraglomerular pressure. Raised intraglomerular pressure is believed to be central to the “final common pathway” of disease progression in chronic kidney disease (CKD). Since SGLT-2 inhibition with empagliflozin also causes glycosuria and acute haemodynamic changes in kidney function in people without diabetes, empagliflozin may also be nephroprotective in conditions without ambient hyperglycaemia, which collectively account for 50 to 70% of patients with CKD worldwide. Patients with established CKD are at substantial risk of progressing to end-stage kidney disease despite the use of medical therapies, including renin-angiotensin system inhibition, so identifying new treatments to delay progression is a priority. Moreover, patients with CKD are at high risk of cardiovascular death and heart failure, which may also be reduced by empagliflozin.

A streamlined international trial

This randomized trial will compare empagliflozin 10 mg once daily versus matching placebo, given on top of standard of care, in around 6000 participants with established CKD, with or without diagnosed diabetes mellitus, who are being treated (where tolerated) with an appropriate dose of a renin-angiotensin system inhibitor. The study is event-driven, and will continue until the required number of primary outcomes has occurred. Follow-up will allow reliable assessment of the effects of empagliflozin on kidney disease progression or cardiovascular mortality, and other clinical outcomes. The study design is streamlined: extra work for collaborating doctors and hospitals will be kept to a minimum, and only essential information will be collected. The trial is focused on readily identifiable and important clinical outcomes. Participant reported information recorded by participant interview directly into bespoke computer systems and centrally measured creatinine are the main means of data collection.

Central Coordinating Office (CCO), Clinical Trial Service Unit and Epidemiological Studies Unit (CTSU),
Richard Doll Building, Old Road Campus, Roosevelt Drive, Oxford OX3 7LF, UK
Tel: +44(0)1865 743868, E-mail: cco.empakidney@ndph.ox.ac.uk

Table of contents

1.1 DOES INHIBITION OF SODIUM-GLUCOSE CO-TRANSPORTER-2 (SGLT-2) WITH EMPAGLIFLOZIN PREVENT KIDNEY DISEASE PROGRESSION OR CARDIOVASCULAR DEATH IN PATIENTS WITH CHRONIC KIDNEY DISEASE (CKD)? 6
1.1.1 Substantial cardiovascular risk exists for CKD patients despite statin-based therapy and antihypertensive therapy 6
1.1.2 Empagliflozin reduces the risk of cardiovascular death in people with type 2 diabetes and established cardiovascular disease 6
1.1.3 Substantial risk of kidney disease progression in people with CKD despite inhibition of the renin-angiotensin system 6
1.1.4 Empagliflozin may be nephroprotective in patients without diabetes 7
1.1.5 The safety of empagliflozin has been established in people with type 2 diabetes 8
2.1 STUDY AIMS 9
2.2 TREATMENT COMPARISONS 9
2.2.1 Run-in period prior to randomization 9
2.2.2 Randomization to empagliflozin versus placebo 9
2.3 DATA ANALYSIS PLAN 10
2.3.1 Main and subsidiary assessments 10
2.3.1.1 Primary assessment 10
2.3.1.2 Secondary assessments 10
2.3.1.3 Tertiary efficacy assessments 11
2.3.2 Safety, biochemical and exploratory assessments 12
2.3.2.1 Safety assessments 12
2.3.2.2 Biochemical assessments 13
2.3.2.3 Exploratory assessments 13
2.3.2.4 Health economic assessments 13
2.3.3 Statistical analysis 13
2.4 SAMPLE SIZE AND PREDICTED NUMBER OF EVENTS 14
2.4.1 Initial assumptions (prior to study start) 14
2.4.1.1 Anticipated effects of empagliflozin on the primary outcome 14
2.4.1.2 Planned study duration and statistical power 14
2.5 DATA AND SAFETY MONITORING 14
2.5.1 Recording and reporting of adverse events 14
2.5.1.1 Recording of Adverse Events (AEs) 14
2.5.1.2 Recording and review of relevant AEs by LCC staff 15
2.5.1.3 Collection of Additional Information for Suspected Serious Adverse Reactions (SSARs) by CCO 17
2.5.1.4 Expedited reporting of SUSARs and exemptions from expedited reporting 17
2.5.2 Interim analyses: role of the independent Data Monitoring Committee 18
2.5.2.1 Regular unblinded analyses by the DMC 18
2.5.2.2 Early stopping for benefit 18
2.6 CENTRAL AND REGIONAL COORDINATION OF LOCAL CLINICAL CENTRES 19
2.6.1 Training and quality assurance 19
2.6.2 Supply of study treatment 20
2.6.3 Data management 20
2.6.4 Biological sample assay, transport and storage 21
2.6.4.1 Local analysis of eligibility and safety bloods 21
2.6.4.2 Central assessment of samples collected at the randomization visit and during follow-up 21
2.6.4.3 Consent approval for unspecified analyses on blood and urine samples 21
2.6.5 Administrative details 22
2.6.5.1 Source documents and archiving 22
2.6.5.2 Funding 22
2.6.5.3 Indemnity 22
2.6.5.4 End of the within-trial period 22
2.6.5.5 Publications and reports 22
2.6.5.6 Substudies 23
3.1 ELIGIBILITY FOR THE STUDY 25
3.1.1 Inclusion criteria 25
3.1.2 Exclusion criteria 25
3.2 IDENTIFICATION AND INVITATION 26
3.2.1 Identification and invitation of potentially eligible participants 26
3.3 SCREENING VISIT AND PRE-RANDOMIZATION RUN-IN 26
3.3.1 Assessment of relevant medical history and eligibility 26
3.3.2 Written consent 26
3.3.3 Confirmation of eligibility and collection of blood and urine samples 27
3.3.4 Review of eligibility and renin-angiotensin system inhibition by a Local Investigator 27
3.4 RANDOMIZATION VISIT (0 MONTHS) 28

3.4.1 Final check of eligibility and compliance before randomization 28
3.4.1.1 Collection of blood and urine samples 28
3.4.2 Random allocation of study treatment 28
3.5 FOLLOW-UP VISITS (2 AND 6 MONTHS AND THEN 6-MONTHLY) 29
3.5.1 Recording adverse events and adherence to study treatment 29
3.5.2 Collection of blood and urine samples 29
3.5.3 Issuing study treatment and arranging further appointments 30
3.5.4 Follow-up for randomized participants not attending study clinics 30
3.5.5 Monitoring of women of child bearing potential 30
3.5.6 Monitoring of people with diabetes mellitus 30
3.5.7 Local Lead Investigator supervision 31
3.6 CENTRAL MONITORING OF PARTICIPANT SAFETY, EARLY RECALL VISITS AND MODIFYING STUDY TREATMENT 31
3.6.1 Early Recall Visits 31
3.6.2 Monitoring liver function, potassium, creatinine and AESIs 31
3.6.3 Modifying study treatment 31
3.6.4 Unblinding of study treatment 32
3.6.5 Withdrawal of consent 32
3.7 CONFIRMATION AND VERIFICATION OF STUDY OUTCOMES ("ADJUDICATION") 32
3.8 CITED REFERENCES 33
4.1 APPENDIX 1: ORGANISATIONAL STRUCTURE AND RESPONSIBILITIES FOR STUDY DESIGN AND CONDUCT 37
4.2 APPENDIX 2: VISIT SCHEDULE AND PROCEDURES 40
4.2.1 Clinic procedures 40
4.3 APPENDIX 3: STUDY ADDRESSES 41
5.1 VERSION HISTORY 42

TRIAL SYNOPSIS

Trial title	A multicentre international randomized parallel group double-blind placebo-controlled clinical trial of EMPAgliflozin once daily to assess cardio-renal outcomes in patients with chronic KIDNEY disease
Short and lay title	EMPA-KIDNEY (The study of heart and kidney protection with empagliflozin)
Clinical phase	III
Trial design	Randomized double-blind placebo-controlled trial
Responsibilities	The study was initiated by the University of Oxford and developed in a collaboration with Boehringer Ingelheim, which has provided funding for the trial. Boehringer Ingelheim, the sponsor of this trial, has delegated responsibility for the conduct, analysis and reporting of the trial to the University of Oxford.
Boehringer Ingelheim ID	1245-0137
ClinicalTrials.gov	NCT03594110
EudraCT number	2017-002971-24
Trial participants	Eligibility criteria:<br>1. Aged ≥18 years* at Screening; and<br>2. Chronic kidney disease (CKD) at risk of kidney disease progression;†<br>3. A local investigator judges that the participant neither requires empagliflozin (or any other SGLT-2 or SGLT-1/2 inhibitor), nor that such treatment is definitely inappropriate; and<br>4. No exclusion criteria apply<br>Participants will be treated with appropriate doses of renin-angiotensin system (RAS)-inhibition, unless such treatment is either not tolerated or not indicated. No patient currently being treated with empagliflozin (or other SGLT-2 or SGLT-1/2 inhibitor) should be taken off this therapy to meet the eligibility criteria. Throughout the study, the care of participants will remain the responsibility of their local doctors who will be asked to ensure individualized standard of care, including management of cardiovascular risk factors and other existing comorbidities (e.g. hypertension, diabetes etc.). This should be conducted in the context of prevailing local, national or international guidance.
Planned sample size	Approximately 6000 participants, including at least one-third with diabetes, one-third without diabetes, and up to one-third with a CKD-EPI estimated glomerular filtration rate (eGFR) ≥45 and <90 mL/min/1.73m^2 at Screening
Placebo Run-in	8-12 weeks
Treatment duration	Event driven: the trial will continue until at least 1070 participants have experienced a first primary outcome after randomization
Primary outcome	Time to first occurrence of:<br>• Kidney disease progression (end-stage kidney disease‡, a sustained eGFR <10 mL/min/1.73m^2, renal death, or a sustained ≥40% decline in eGFR from randomization) or<br>• Cardiovascular death
Secondary outcomes	Key secondary outcomes:<br>• Time to first hospitalization for heart failure or cardiovascular death<br>• Time to occurrences of all-cause hospitalization (first and recurrent combined)<br>• Time to death from any cause<br>Other secondary outcomes:<br>• Time to kidney disease progression<br>• Time to cardiovascular death<br>• Time to cardiovascular death or end-stage kidney disease
Medicinal Product	Oral empagliflozin 10 mg
Formulation, dose, route of administration	Run-in: placebo film-coated tablet once daily (single-blind) for oral administration;<br>From randomization: empagliflozin 10 mg film-coated tablet once daily versus matching placebo film-coated tablet once daily (double-blind) for oral administration

* Or "full age" as required by local regulations (e.g. 20 years in Japan)

† Either (i) estimated glomerular filtration rate (eGFR) ≥20, <45 mL/min/1.73m^2; or (ii) eGFR ≥45, <90 mL/min/1.73m^2 with urine albumin:creatinine ratio ≥200 mg/g

‡ End-stage kidney disease is defined as the initiation of maintenance dialysis or receipt of a kidney transplant

PROTOCOL ABBREVIATIONS AND GLOSSARY

Abbreviation	Definition	Comment
ALT/AST	Alanine/Aspartate Transaminase	Liver transaminases
AE/ AESI/ SAE/ SSAR/ SUSAR	Adverse Event/ Adverse Event of Specialist Interest/ Serious Adverse Event/ Suspected Serious Adverse Reaction/ Suspected Unexpected Serious Adverse Reaction	
CCO	Central Coordinating Office	The Central Co-ordinating Office based at CTSU in Oxford, responsible for the overall coordination of the study
CCO study clinician	Central Coordinating Office study clinician	One of a group of CCO doctors responsible for monitoring the safety of participants through review of AEs and local laboratory results
CI	Confidence Interval	
CKD	Chronic Kidney Disease	
CTSU	Clinical Trial Service Unit and Epidemiological Studies Unit	Home of the CCO at the University of Oxford
DMC	Data Monitoring Committee	
eGFR	Estimated Glomerular Filtration Rate	
ESKD	End-Stage Kidney Disease	
HbA1c	Glycated Haemoglobin	
HR	Hazard Ratio	
ICH-GCP	International Conference for the Harmonisation of Good Clinical Practice	Guidance for conducting clinical studies
IRB/REB	Institutional Review Board / Research Ethics Board	
LCC	Local Clinical Centre	
LCC clinic staff	Local Clinical Centre clinic staff	The LCC Research Coordinators and Local Investigators
LCC Research Coordinator	Local Clinical Centre Research Coordinator	The person(s) conducting the participant interviews, usually a qualified nurse, but in some cases may be medically qualified or have other relevant qualifications and experience. All individuals fulfilling this role will receive appropriate training organized by the RCC
Local doctor		Any doctor who has clinical responsibility for the care of the participants, including their primary care doctor or a hospital doctor
LLI	Local Lead Investigator	The doctor responsible for the trial at a LCC who is supported by other Local Investigators and LCC Research Co-ordinators (to whom certain trial-related activities are delegated).
MedDRA	Medical Dictionary for Drug Regulatory Activities	
NT-proBNP	N-terminal Prohormone of Brain Natriuretic Peptide	
PI	Principal Investigator	The grant holders for the study at the University of Oxford, who are collectively responsible for the conduct of the trial.
RAS-inhibitors (ACEi/ARB)	Renin-Angiotensin System inhibitors (Angiotensin-Converting Enzyme inhibitor/ Angiotensin Receptor Blocker)	
RCC	Regional Coordinating Centre	
SGLT	Sodium-Glucose Co-transporter	
SOP	Standard Operating Procedure	
T2D	Type 2 Diabetes	
ULN	Upper Limit of Normal	
WOCBP	Women of Child Bearing Potential	

1.1 DOES INHIBITION OF SODIUM-GLUCOSE CO-TRANSPORTER-2 (SGLT-2) WITH EMPAGLIFLOZIN PREVENT KIDNEY DISEASE PROGRESSION OR CARDIOVASCULAR DEATH IN PATIENTS WITH CHRONIC KIDNEY DISEASE (CKD)?

1.1.1 Substantial cardiovascular risk exists for CKD patients despite statin-based therapy and antihypertensive therapy

In high-income countries, the prevalence of CKD is about 10% and is likely to increase as average population age rises and diabetes mellitus becomes more prevalent.[1, 2] Cardiovascular risk increases progressively as kidney function declines.[3, 4] There is evidence that lowering low-density lipoprotein cholesterol and blood pressure in people with CKD reduces cardiovascular risk,[5, 6] but substantial residual risk remains and no other treatments have been shown to reduce cardiovascular risk in this group of patients.

A key feature of cardiovascular disease in CKD is presence of structural heart pathologies (e.g. left ventricular hypertrophy and/or dilatation) and heart failure (which may be accompanied by coronary heart disease). At least half of patients with advanced CKD (i.e. stages 4-5) have abnormal cardiac structure on echocardiography,[7,8] increasing to over 80% by the time dialysis is initiated.[8]

1.1.2 Empagliflozin reduces the risk of cardiovascular death in people with type 2 diabetes and established cardiovascular disease

Selective inhibition of SGLT-2 causes increased urinary glucose and transiently increased sodium excretion. This is associated with reductions in weight and blood pressure as well as haemoglobin glycation (HbA1c). Among 7020 patients with type 2 diabetes mellitus (T2D) and established cardiovascular disease in the EMPA-REG OUTCOME trial, empagliflozin reduced the primary cardiovascular composite outcome (death from cardiovascular causes, nonfatal myocardial infarction, or nonfatal stroke) by 14% compared to placebo (hazard ratio [HR] 0.86, 95% confidence interval [CI] 0.74-0.99). This was driven by a significant reduction in cardiovascular death (HR 0.62, 95% CI 0.49-0.77, nominal $p<0.0001$). A pre-specified secondary outcome of hospitalization for heart failure was reduced by 35% (HR 0.65, 95% CI 0.50-0.85).[9]

The EMPA-REG OUTCOME trial was conducted in participants with relatively preserved kidney function (>90% had a baseline estimated glomerular filtration rate [eGFR] >45 mL/min/1.73m^2), and it is unclear whether empagliflozin can prevent cardiac disease in patients with more severe kidney impairment.

1.1.3 Substantial risk of kidney disease progression in people with CKD despite inhibition of the renin-angiotensin system

CKD is often a progressive condition, with proteinuria representing a significant risk factor for a more rapid decline in kidney function.[10] Although patients with early CKD are more likely to die before they reach end-stage kidney disease (ESKD), the avoidance of ESKD is still highly desirable due to its adverse effects on quality of life and the substantial costs of dialysis and transplantation to healthcare providers. Inhibition of the renin-angiotensin system (RAS) with angiotensin-converting enzyme inhibitors (ACEi) or angiotensin receptor blockers (ARB) reduces albuminuria and slows the rate of progression in proteinuric nephropathies, particularly in diabetic kidney disease.[11-13] However, a substantial residual risk of ESKD remains. Although combination therapy (i.e. ACEi plus ARB) was initially thought to be a promising approach, such combined regimens do not delay kidney disease progression and may cause hyperkalaemia or acute kidney injury.[14] There is therefore a

need for new treatments that can be added safely to current standard treatments in order to slow progression to ESKD.

SGLT-2 inhibition with empagliflozin shows the potential to reduce the risk of kidney disease progression in people with T2D. An exploratory analysis of the EMPA-REG OUTCOME trial indicated that empagliflozin reduced the incidence of the composite outcome of doubling of creatinine, the need to start kidney replacement therapy or renal death by 46% (HR 0.54, 95% CI 0.40-0.75).[15] This nephroprotective effect occurred on the background of an initial decrease in eGFR over the first 4 weeks of treatment among those allocated to empagliflozin (Figure 1 from EMPA-REG OUTCOME below). The magnitude of the kidney effect associated with empagliflozin was consistent at doses of 10 mg and 25 mg (Figure 1), and across pre-specified subgroups of kidney function and parameters indicative of kidney damage, including patients with prevalent CKD (mainly early stage CKD).[15] These benefits were similar regardless of baseline ACEi or ARB use and there was no evidence of an increased risk of hyperkalaemia or acute kidney injury.[15] However, it is not possible to draw definite conclusions about the effects of SGLT-2 inhibition with empagliflozin in people with more established CKD (indeed, empagliflozin is currently not licensed for use in people with an eGFR <45 mL/min/1.73m^2).

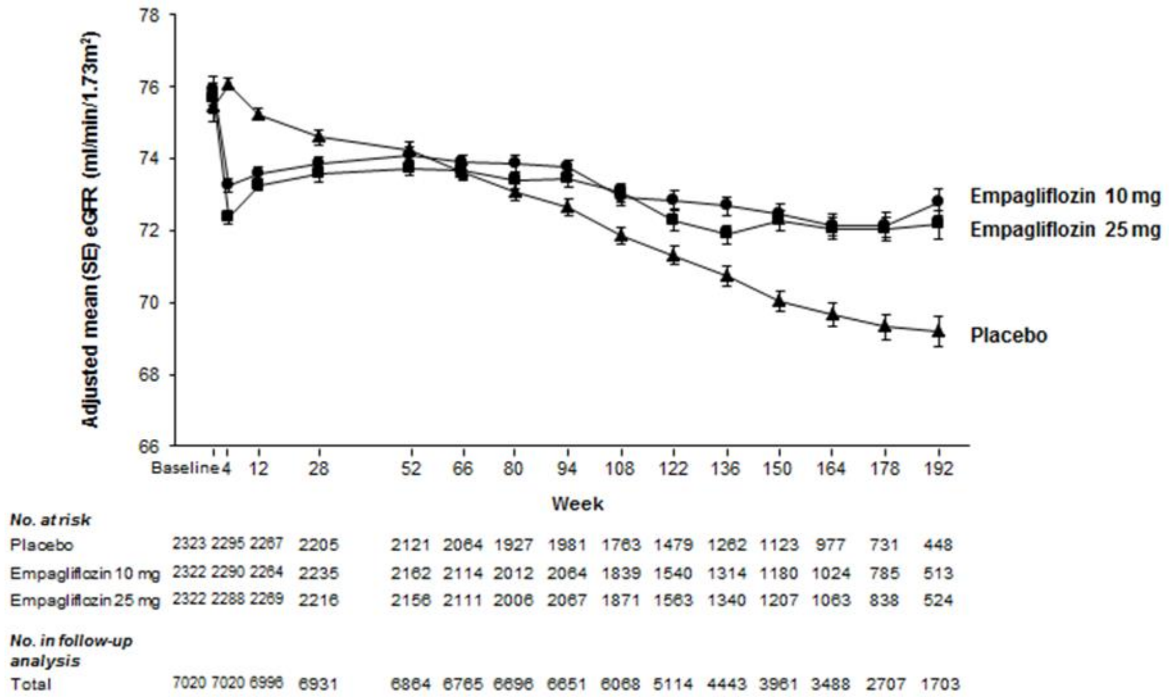

Figure 1: *Change in eGFR in the EMPA-REG OUTCOME trial by treatment allocation*[15]

1.1.4 Empagliflozin may be nephroprotective in patients without diabetes

Experimental and clinical studies suggest that tubular dysregulation may drive kidney disease progression in a wide range of patients with advanced CKD. This dysregulation is characterized by excessive reabsorption of sodium in the early proximal tubule, mediating afferent arteriolar vasodilatation and, consequently, causing intraglomerular hypertension and associated glomerular barotrauma.[16-18] In support of the hypothesis that intraglomerular hypertension is a final common pathway for progression for many forms of CKD, it has been observed that, for a given level of urinary albumin excretion, the risks of ESKD are relatively independent of the primary cause of kidney disease.[19] The mechanisms behind the kidney effects of empagliflozin are likely multifactorial but direct kidney haemodynamic effects are considered to play an important role. Empagliflozin reduces proximal tubular sodium

reabsorption, thereby increasing distal sodium delivery to the macula densa, which has been shown to activate a tubulo-glomerular feedback leading to afferent arteriolar vasoconstriction, thereby reducing intraglomerular pressure and urinary albumin excretion.

Empagliflozin has also been shown to have a pharmacological effect in people without diabetes. In healthy volunteers, empagliflozin 10 mg daily resulted in approximately 50 g/day glycosuria, and an initial acute decrease in GFR (an indicator of reduced intraglomerular pressure) has been shown to occur in overweight but otherwise healthy volunteers (unpublished data, BI clinical trial report 1245.66). Taken together, these observations suggest that empagliflozin has haemodynamic effects in the kidney in the absence of elevated blood glucose.

It is therefore reasonable to hypothesize that empagliflozin may have beneficial effects on kidney disease progression and cardiovascular risk among those with CKD, irrespective of the presence of diabetes. Worldwide, the proportion of patients with CKD who have diabetes ranges from about 30 to 50% so, if empagliflozin has beneficial effects on kidney and cardiovascular outcomes in CKD, then its use in patients with CKD but without diabetes would increase the potential population who might benefit from this drug by 2-3 times.[20, 21]

1.1.5 The safety of empagliflozin has been established in people with type 2 diabetes

The empagliflozin clinical development programme has randomized >15,000 trial participants to date. About 550 healthy volunteers have been exposed to empagliflozin (up to 800 mg in a single dose and up to 50 mg in multiple dosing), with good tolerability. Approximately 8500 patients with T2D have been treated with empagliflozin in clinical studies, of which more than half have been treated for a year or more.[22-30] In all these studies, empagliflozin was well tolerated. In the EMPA-REG OUTCOME trial, which had a median follow-up of 3.1 years, the frequency of serious adverse events (SAEs) and adverse events that led to discontinuation of study treatment among patients allocated empagliflozin was no higher than that among those allocated placebo.[9, 15] There was no significant increase in the frequency of hypoglycaemia with empagliflozin, except when used in combination with a sulphonylurea or basal dose insulin.[31] Electrolytes were not significantly different among those allocated to empagliflozin or placebo.[31] Compared to placebo, there was an increased frequency of mycotic genital infections. By contrast with the increased risk of bone fracture and lower-limb amputation observed with another SGLT-2 inhibitor, canagliflozin,[32] there was no such adverse safety signal observed when over 12,000 patients with T2D from placebo-controlled empagliflozin clinical trials (including EMPA-REG OUTCOME) were analysed together.[31] Further safety analyses from EMPA-REG OUTCOME showed that the adverse event profile of empagliflozin in patients who had impaired kidney function at baseline (i.e. eGFR <60 mL/min/1.73m^2), a potentially vulnerable population, was consistent with that reported in the overall trial population.[15] In summary, the EMPA-KIDNEY trial aims to assess whether empagliflozin reduces the risk of kidney disease progression or cardiovascular death in people with CKD, irrespective of whether they have diabetes, and whether the benefits of treatment outweigh any adverse effects.

2.1 Study Aims

The study will randomize approximately 6000 participants with pre-existing CKD (at least one-third with diabetes and one-third without diabetes) between empagliflozin 10 mg daily and matching placebo on top of standard of care. The trial will continue until a minimum number of 1070 primary outcomes has accrued (i.e. the trial is event-driven). The primary aim is to assess the effect of empagliflozin on time to kidney disease progression or cardiovascular death (see Section 2.3.1.1).

The key secondary aims are to assess the effect of empagliflozin on time to hospitalization for heart failure or cardiovascular death, occurrences of hospitalizations from any cause, and time to death from any cause (see Section 2.3.1.2). Other assessments, including analyses of safety, are also planned, and are described in Sections 2.3.1.2 to 2.3.2.4.

2.2 Treatment Comparisons

2.2.1 Run-in period prior to randomization

Prior to randomization, potentially eligible participants will enter an 8-12 week Run-in period, during which they will receive single-blind placebo tablets. The purpose of the Run-in period is to help ensure that only those likely to continue taking study treatment for an extended period are randomized (see Figure 2).

Information collected at the Screening Visit will be provided to Local Investigators, who will be asked to confirm that in their judgment the participant:

(i) Neither requires empagliflozin (or any other SGLT-2 or SGLT-1/2 inhibitor), nor that such treatment is definitely inappropriate; and

(ii) Has been prescribed an appropriate dose of a RAS-inhibitor, unless such treatment is either not tolerated or not indicated (see Section 3.3.4).

At Screening and throughout the study, the care of participants will remain the responsibility of local doctors who are asked to ensure individualized standard of care, including management of cardiovascular risk factors and other existing comorbidities (e.g. hypertension, diabetes etc.). It is advised that prevailing local, national or international guidance is considered (see Section 3.3.4).

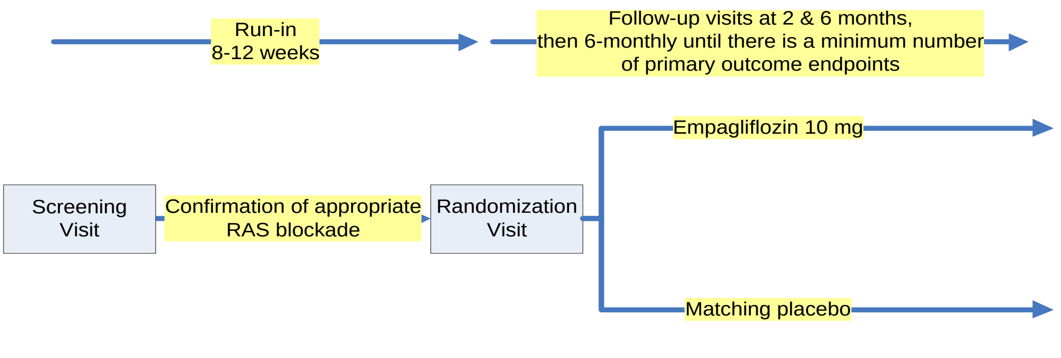

Figure 2. *Outline of 1:1 randomization and follow-up schedule*

2.2.2 Randomization to empagliflozin versus placebo

Eligible and consenting individuals will be allocated empagliflozin or placebo using a minimized randomization algorithm that helps ensure balance between the treatment groups with respect to the following prognostic variables: age, sex, prior diabetes, eGFR and urinary

albumin:creatinine ratio (both based on local laboratory results at screening), and region.[33] The algorithm includes a stochastic element (treatment is assigned to the arm determined by the minimization algorithm with a probability of 0.9 and by a random number generator with a probability of 0.1). Given the stochastic element of the randomization, re-randomization methods for the analysis are not considered necessary and only traditional methods of analysis are planned. Randomized participants will be issued with a 7-month supply of study treatment consisting of empagliflozin 10 mg or matching placebo. One tablet is to be taken daily with or without food. To ensure a dose interval of about 24 hours, the medication should ideally be taken at approximately the same time every day.

2.3 DATA ANALYSIS PLAN

2.3.1 Main and subsidiary assessments

2.3.1.1 Primary assessment

The primary assessment will involve an intention-to-treat comparison among all randomized participants, using a Cox model adjusting for each of the minimization variables (see above), of the effects of allocation to empagliflozin versus placebo on the time to the first occurrence of:

(i) Kidney disease progression (defined as ESKD, a sustained decline in eGFR to <10 mL/min/1.73m^2, renal death, or a sustained decline of ≥40% in eGFR from randomization); or

(ii) Cardiovascular death.

ESKD is defined as the initiation of maintenance dialysis or receipt of a kidney transplant.

To ensure bias is not introduced by differences between treatment arms in the extent to which extra eGFR measurements are made outside of scheduled follow-up visits, the term 'sustained' in respect of a decline in eGFR (to <10 mL/min/1.73m^2, or of ≥40% from baseline) is that it is either (a) measured at two consecutive scheduled study follow-up visits; or (b) measured at the last scheduled study follow-up visit or the last scheduled visit before death (or withdrawal of consent).

2.3.1.2 Secondary assessments

If the primary outcome is statistically significant (either at the interim or final analysis), the key secondary outcomes will then be tested. The secondary assessments will involve intention-to-treat comparisons among all randomized participants of the effects of allocation to empagliflozin versus placebo during the scheduled treatment period on:

(i) Key secondary outcomes:
   - a) Time to first hospitalization for heart failure or cardiovascular death;
   - b) Time to occurrences of all-cause hospitalizations (first and recurrent combined);
   - c) Time to death from any cause.

(ii) Other secondary outcomes:
   a) Time to first occurrence of kidney disease progression;
   b) Time to cardiovascular death;
   c) Time to cardiovascular death or ESKD.

In testing the key secondary outcomes, their p-values will be corrected for multiple testing using the Hochberg “step-up” procedure that controls the familywise error rate. Other secondary outcomes will be assessed without adjustment for multiplicity at a nominal level of $\alpha = 0.05$ (two-sided).

2.3.1.3 Tertiary efficacy assessments

Tertiary assessments will involve intention-to-treat analyses among all randomized participants of the effects of allocation to empagliflozin versus placebo during the scheduled treatment period on:

2.3.1.3.1 Renal tertiary outcomes

(i) Time to components of kidney disease progression defined as follows:
   (a) ESKD, a sustained decline in eGFR to <10 mL/min/1.73m^2, or renal death;
   (b) Sustained decline of ≥40% in eGFR from randomization;

(ii) Annual rate of change in eGFR, calculated separately:
   (a) For the whole follow-up period;
   (b) From 2 months until the last scheduled visit;

2.3.1.3.2 Mortality-based tertiary outcomes

(iii) Time to ESKD or death from any cause combined;

(iv) Time to kidney disease progression or death from any cause combined;

(v) Time to death from particular categories of causes, including cardiovascular (coronary death, other cardiac [including heart failure and sudden cardiac death not known to be coronary], stroke, other cardiovascular and presumed cardiovascular) and non-cardiovascular (e.g. renal, infection, cancer, other medical, and non-medical) causes;

2.3.1.3.3 Cardiovascular and metabolic tertiary outcomes

(vi) Time to major cardiovascular events (defined as the composite of cardiovascular death, myocardial infarction, stroke or hospitalization for heart failure);

(vii) Time to new-onset diabetes mellitus (defined as clinical diagnosis, commencement of glucose-lowering treatment, or HbA1c ≥48 mmol/mol measured by central laboratory on at least one occasion) among participants without diabetes at baseline*, overall and separately among those with normoglycaemia or “pre-diabetes” (defined as HbA1c <39 mmol/mol [normoglycaemia] and ≥39 to <48 mmol/mol [pre-diabetes], respectively);

(* diabetes at baseline is defined as participant-reported history of diabetes, use of glucose-lowering medication or baseline HbA1c ≥48 mmol/mol at Randomization visit).

(viii) Time to self-reported episode of gout;

2.3.1.3.4 Subgroup analyses

(ix) Subgroup analyses are planned for the primary composite outcome;

Pre-specified categories for subgroup analyses are defined as follows:

- History of prior disease (presence vs. absence): diabetes mellitus; cardio-vascular disease; heart failure; peripheral arterial disease;
- Age; sex; region; blood pressure; body mass index;
- Laboratory values: HbA1c; eGFR; urinary albumin:creatinine ratio; NT-proBNP; haematocrit (see Section 2.3.3 for approach to grouping);
- Medication: RAS-inhibition; beta-blocker; diuretics.

The subgroup analyses of the primary composite outcome which are of key interest are those involving subdivision by: (a) baseline diabetes status, (b) baseline eGFR, and (c) urinary albumin:creatinine ratio.

2.3.2 Safety, biochemical and exploratory assessments

2.3.2.1 Safety assessments

Safety assessments will involve intention-to-treat among all randomized participants and, where appropriate, on-treatment analyses of the effects of allocation to empagliflozin versus placebo during the scheduled treatment period on:

(i) SAEs due to:
  (a) Urinary tract infection, overall and separately by sex;
  (b) Genital infection, overall and separately by sex;
  (c) Hyperkalaemia;
  (d) Acute kidney injury;
  (e) Dehydration;

(ii) AEs of Special Interest (AESIs):
  (a) Liver injury, both overall and separately by cause (defined as ALT or AST ≥5x Upper Limit of Normal [ULN] or the combination of ALT or AST ≥3x ULN with bilirubin ≥2x ULN; measured in the same blood sample at study follow-up or early recall visits; see Section 3.5.2);
  (b) Ketoacidosis, both overall and, separately, by baseline diabetes status;
  (c) Lower limb amputations (overall and by level);

(iii) Other AEs relevant to the study question:
  (a) Bone fractures, both overall and separately by site and aetiology (i.e. distinguishing those resulting from high and low impact trauma);
  (b) Severe hypoglycaemia (defined as low blood sugar causing severe cognitive impairment which requires assistance from another person for recovery);
  (c) Symptomatic dehydration (defined as whether or not a participant has experienced symptoms they attribute to dehydration, such as feeling faint or fainting);

(iv) Hospitalization by specific causes†;

(v) SAEs both overall and, separately, by category†;

(vi) Discontinuation of study treatment overall and by various causes (including SAEs†, non-serious adverse events†, and other reasons);

† based on Medical Dictionary for Drug Regulatory Activities (MedDRA) System Organ Class classification

(vii) Changes in weight and systolic and diastolic blood pressure from baseline.

2.3.2.2 Biochemical assessments

Additional biochemical assessments at the central laboratory on urine and blood (collected at the Randomization visit, 2 months, 18 months and the Final-follow-up visit) will involve intention-to-treat analyses among all randomized participants of the effects of allocation to empagliflozin versus placebo during the scheduled treatment period on:

- Urine albumin:creatinine ratio
- HbA1c.

Biochemical assessments using local laboratory results will involve intention-to-treat analyses among all randomized participants, and where appropriate, on-treatment analyses of the effects of allocation to empagliflozin versus placebo during the scheduled treatment period on:

- Potassium
- ALT/AST (including elevations of ALT/AST in various categories)
- Sodium, corrected calcium and phosphate (in a subset of about 20%)
- Haematocrit and haemoglobin (in a subset of about 20%).

2.3.2.3 Exploratory assessments

Exploratory assessments may also be made of other possible beneficial or adverse effects of empagliflozin, including secondary or tertiary outcomes by pre-specified subgroups, on mean eGFR at each scheduled visit and at the 4-week post-Final Follow-up blood draw (see Section 3.5.2), urine albumin:creatinine ratio 4 weeks after Final Follow-up, and how treatment effects vary by time since Randomization. In interpreting the results of any exploratory analyses that will be performed, allowance will be made for multiple hypothesis testing, their exploratory (and, perhaps, data-dependent) nature, and for evidence from other studies. Analyses of fatal events will be interpreted in the light of the observed effects on relevant non-fatal events.[34]

2.3.2.4 Health economic assessments

The study results may, if appropriate, be used to conduct health economic assessments regarding the use of empagliflozin. An analysis plan will be pre-specified if any such analyses are considered worthwhile.

2.3.3 Statistical analysis

A full Data Analysis Plan will be finalised prior to any unblinding of study results. Briefly, all participants randomized to empagliflozin will be compared with all participants randomized to placebo, regardless of whether a participant received all, some or none of their allocated treatment (i.e. intention-to-treat analyses). A participant may contribute to more than one assessment if they have events of more than one type (e.g. hospitalization for heart failure followed by ESKD). For the time-to-event analyses survival analytic methods will be used to evaluate the time to the first event during the entire study period. For each categorical outcome, Cox proportional hazards regression adjusted for the prognostic variables used in the minimization algorithm (age, sex, prior diabetes, eGFR, urinary albumin:creatinine ratio, and region) will be used to estimate the hazard ratio comparing all those allocated active empagliflozin with all those allocated placebo. Estimates of the hazard ratio will be shown with 95% confidence intervals, and Kaplan-Meier estimates for the time to each of the primary and secondary outcomes will also be plotted. For the secondary outcome of all-cause hospitalization, the analysis will examine all events (i.e. not just the first event in each participant).

Tests for heterogeneity of the proportional effect observed in subgroups, through the inclusion of relevant interaction terms in Cox models, will be used to determine whether the proportional effects in specific subcategories are clearly different from the overall effect. Where categories can be arranged in a meaningful order (e.g. age at randomization) then assessment of any trend will be made. For subgroups based on continuous variables (e.g. blood pressure), participants will be subdivided into approximately equal thirds based on the tertiles of the relevant distribution or, where appropriate, by conventional thresholds. Details of the sub-group classifications will be described in the Data Analysis Plan.

For analyses of continuous variables, such as blood pressure and analyses of biochemical effects (see Section 2.3.2.2), differences in means between the randomized groups will be assessed (after appropriate transformation, where necessary).

The more detailed Data Analysis Plan will provide methods for recurrent event analyses, planned sensitivity analyses (including the plotting of cumulative incidence functions), handling of model covariates and missing data, censoring rules and alternative methods of analysis for situations where there are issues with the fit of the main analysis model.

2.4 SAMPLE SIZE AND PREDICTED NUMBER OF EVENTS

2.4.1 Initial assumptions (prior to study start)

2.4.1.1 Anticipated effects of empagliflozin on the primary outcome

Whilst the EMPA-REG OUTCOME trial was conducted exclusively among people with T2D, it is anticipated that EMPA-KIDNEY will include a substantial proportion of participants without diabetes (at least one third), who are expected to experience smaller changes in glycosuria than those with diabetes (see Section 1.1.4). Since smaller changes in glycosuria in participants without diabetes may translate into smaller relative effects than in those with diabetes, it has been assumed that the relative reductions for both components of the primary outcome (i.e. cardiovascular death and kidney disease progression) in EMPA-KIDNEY will be about half as large as was observed in EMPA-REG OUTCOME.

2.4.1.2 Planned study duration and statistical power

The trial will randomize approximately 6000 participants from about 200-250 sites and continue until a minimum of 1070 primary outcome events has occurred. Such an event-driven trial would provide an overall power of 90% at p=0.05 (two-sided) to detect an 18% relative reduction in the primary outcome (time to kidney disease progression or cardiovascular death). During the trial, the Steering Committee will monitor event rates for the primary outcome and its components blind to treatment allocation, and if necessary, may consider proposing changes to the protocol. A formal interim analysis may be performed after 150 ESKD events have occurred (see Section 2.5.2.2 for details).

2.5 DATA AND SAFETY MONITORING

2.5.1 Recording and reporting of adverse events

2.5.1.1 Recording of Adverse Events (AEs)

The trial focuses on important clinical outcomes and is reliant upon both participant reported information and centrally measured kidney function as the primary means of data collection. It is not expected or required that medical records will be reviewed by Local Clinical Centre (LCC) clinic staff or monitoring staff to identify AEs, SAEs or other trial outcomes since the participants will be used as the primary source of information. Procedures for central adjudication of potential study outcomes are described in Section 3.7.

The safety profile of empagliflozin has been well-studied in previous trials.[9, 15, 22-31] Therefore, in line with regulatory guidance,[35] collection of safety data will be streamlined.

2.5.1.1.1 Non-serious adverse events

Non-serious AEs will only be recorded if they:

(a) Lead to discontinuation of study treatment; or

(b) Are one of the following:

- Bone fracture (with additional information recorded about fracture site and aetiology [i.e. distinguishing those resulting from high and low impact])
- Severe hypoglycaemia (as defined in Section 2.3.2.1)
- Episodes of gout
- Symptomatic dehydration (as defined in Section 2.3.2.1)
- An Adverse Event of Special Interest (AESI; see Section 2.5.1.1.2)
- Events that could lead to amputation (which include diagnosis or treatment for peripheral arterial disease, peripheral neuropathy, diabetic foot ulcer, and lower limb infection or gangrene).

2.5.1.1.2 Adverse Events of Special Interest (AESI)

The following AEs will be recorded regardless of whether they fulfil the criteria for a SAE:

- Liver injury
- Ketoacidosis
- Lower limb amputations (overall and by level).

All new AESIs will be reviewed each working day by Central Co-ordinating Centre (CCO) clinicians and relevant additional details sought promptly (see Section 3.6.2). Detailed reports on AESIs will be provided by the CCO to Boehringer Ingelheim at regular intervals.

2.5.1.1.3 Serious Adverse Events (SAEs)

SAEs are defined as those adverse events that:

- Result in death
- Are life-threatening
- Require inpatient hospitalization or prolongation of existing hospitalization;
- Result in persistent or significant disability or incapacity
- Result in congenital anomaly or birth defect
- Are important medical events in the opinion of a responsible Local Investigator (i.e. not life-threatening or resulting in hospitalization, but may jeopardise the participant or require intervention to prevent one or other of the outcomes listed above).

For the purposes of this trial, certain pre-specified Preferred Terms (e.g. “agranulocytosis”, “interstitial lung disease”), and *all* cancers will always be considered Serious[a].

Pregnancy will not be considered an AE in this trial, but must be reported promptly (within 24 hours) to the Regional Co-ordinating Centre (RCC) or CCO and then followed up using Pregnancy Monitoring Forms.

2.5.1.2 Recording and review of relevant AEs by LCC staff

All relevant AEs (as defined in Section 2.5.1.1) reported by participants at each study visit interview will be recorded and assessed by trained LCC clinic staff (usually the LCC Research Coordinator) directly on the study computer-based data entry system (see Section

[a] In accordance with the European Medicines Agency initiative on Important Medical Events.
Note: New cancer diagnosis and recurrence of pre-existing cancer should all be recorded.

2.6.3), regardless of whether the participant continues to take study treatment or not. If the study team become aware of SAEs or AESIs between study visits, they are requested to report them within 24 hours. If LCC clinic staff cannot access the computer-based data entry system, they must contact their RCC to report the AE within that timeframe. After completion of the trial, investigators do not need to actively monitor participants, but could report SSARs or related AESIs through telephone contact to the RCC or CCO.

The electronic SAE form will capture the following information for all SAEs:

- Unique study identification number of the participant
- Unique SAE form identification number
- The time and date that the SAE form is completed
- The source of the report (e.g. participant, relative, study nurse, Local Investigator, or other doctor)
- A description of the event: Event descriptions will be recorded by the trained clinic staff using MedDRA Preferred Terms. If an appropriate term cannot be identified, advice can be sought from the Local Investigator or a CCO study clinician, or the description can be recorded as free-text and subsequently coded by CCO study clinician, blind to study treatment allocation
- The reason for believing the AE to be serious (i.e. resulted in death, life-threatening, hospitalisation, disabling, congenital anomaly in offspring, other important medical event)
- The date the event started
- The place where the event was diagnosed or managed (e.g. hospital inpatient, hospital outpatient, participant's home)
- The name of the place where the event was diagnosed or managed (if appropriate)
- Number of nights spent in hospital (if applicable)
- The outcome (ongoing, recovered, death, unknown)
- Whether the event is thought likely to be due to study treatment. In making this assessment, there should be consideration, based on the available information, of the pharmacology of the drug and drug class, probability of an alternative cause, the timing of the reaction with respect to study drug, the response to withdrawal of the study drug, and (where appropriate) the response to subsequent re-challenge or dose change.

Such detailed information will also be collected for all AESIs.

The electronic non-serious AE form will capture the following information:

- Unique study identification number of the participant
- Unique AE form identification number
- The time and date that the AE form is completed
- A description of the event (as describe above)
- The date the event started
- The outcome (ongoing, recovered, unknown)
- Whether the event is thought likely to be due to study treatment (as above).

Local Investigators are required to review all AEs recorded by those LCC Research Co-ordinators who have been delegated the task of recording AEs.

2.5.1.3 Collection of Additional Information for Suspected Serious Adverse Reactions (SSARs) by CCO

Any SAE that is considered, with reasonable possibility, to be due to study treatment by either Local Investigators, appropriately delegated LCC clinic staff or CCO study clinicians (or Boehringer Ingelheim staff), is potentially a SSAR. The CCO study clinician will obtain standard information, including participant study number, identity of reporting person, description of event, and reason for attribution to study drug. All such reports will then be forwarded urgently to a CCO Clinical Coordinator (or their delegated CCO study clinician deputy), who will review the evidence for seriousness and relatedness (in discussion with the LLI if necessary), and seek any additional information required (including relevant information relating to medical history and treatment both prior to and following randomization, and prior to/at the time of onset of the SSAR).

2.5.1.4 Expedited reporting of SUSARs and exemptions from expedited reporting

SSARs that are unexpected according to the Investigator's Brochure are subject to expedited reporting.[36, 37] However, in line with recommendations by regulatory authorities, anticipated events that either are efficacy endpoints, consequences of the underlying disease or are events common in the study population will be exempted from expedited reporting in order to protect trial integrity and because based on a single case it is not possible to conclude that there is a reasonable possibility that the investigational drug caused the event.[36, 38] Such events that are exempted from expedited reporting to health authorities in this trial are listed below.

1. Efficacy endpoints:
    - Kidney disease progression (i.e. ESKD)
    - Myocardial infarction
    - Stroke and transient ischaemic attack
    - Heart failure
    - CV death
    - New-onset diabetes mellitus.

2. Common CKD-related events[b]:
    - Acute-on-chronic kidney failure
    - Dialysis and dialysis access related events and complications
    - Bone fractures and parathyroid-related events.

Any SSARs that are considered not exempt will be reported promptly by the CCO to Boehringer Ingelheim, and Boehringer Ingelheim will make an assessment of whether the event is "expected" or not (based on the latest version of the empagliflozin Investigator Brochure). Any SSAR that is unexpected will be considered a potential Suspected Unexpected Serious Adverse Reaction (SUSAR) and will be unblinded by a member of the CCO clinical staff with such privilege.

All SUSARs will be reported to relevant regulatory authorities, to the Chair of the Data Monitoring Committee (DMC) and, as required, to ethics committees and Institutional Review Boards and investigators in an expedited manner in accordance with regulatory requirements.

[b] The relevant MedDRA Preferred Terms which are exempt are specified in the Adverse Event Reporting SOP.

2.5.2 Interim analyses: role of the independent Data Monitoring Committee

2.5.2.1 Regular unblinded analyses by the DMC

The DMC will assess participant safety and the progress of the trial through review of unblinded data at specified intervals, and recommend to the Steering Committee and Boehringer Ingelheim whether to continue, modify, or stop the trial. Measures are in place to ensure blinding of Boehringer Ingelheim, University of Oxford, the Steering Committee and all other trial staff and participants. The tasks and responsibilities of the DMC will be specified in the DMC charter. The DMC will maintain written records of all its meetings.

The DMC will request analyses at a frequency relevant to the stage of the study (typically at 6-12 monthly intervals, with a Chair's review every 3-6 months) or in response to emerging data from other trials. These unblinded analyses of all SAEs and other study outcomes (both overall and in key subgroups, including by region) and all expected SSARs will be supplied in strict confidence by a statistician not otherwise involved in the trial.

The DMC would be expected to advise the Steering Committee if clear evidence emerged of an adverse effect on all-cause mortality (at least 2 standard deviations) or if, in the view of the DMC, there was other compelling evidence of hazard that seemed likely to outweigh any potential benefit.

Unless advised by the DMC in response to clear evidence of hazard, the Steering Committee, collaborators, participants, representatives of the Boehringer Ingelheim, and all study staff will remain blind to these results until the end of the study. The DMC is independent of the University of Oxford and Boehringer Ingelheim.

2.5.2.2 Early stopping for benefit

In addition, the DMC may review a single formal interim efficacy analysis once 150 participants have experienced a first ESKD event (by which time it is expected that approximately 60% of all first primary outcomes will have occurred). Full details of the stopping guidelines at this interim analysis, including the alpha spent at this analysis and the alpha remaining for the final analysis, will be provided in the DMC Charter. Separate alpha-spending functions will be used for the testing of the primary and key secondary outcomes to control the type I error rate across two analysis time-points, and a gatekeeping approach followed by the Hochberg procedure will be used to control the type I error rate across multiple endpoints.

Briefly, in order for the DMC to recommend that the trial is stopped early for benefit at this formal interim analysis, both of the following conditions must be met:

(1) a reduction in the primary outcome with the Hwang-Shih-DeCani alpha-spending function (γ=-8) used to define the required two-sided p-value and its corresponding critical Cox hazard ratio; and (2) a reduction in the secondary composite outcome of time to cardiovascular death or ESKD to at least the same critical Cox hazard ratio as observed in the primary outcome, but with the proviso that the p-value is constrained to be < 0.05. For example, for the scenario when 60% of the first primary outcomes (i.e. 642 first primary outcomes) have occurred at the time of the interim analysis, this would equate to stopping criteria of: (i) a two-sided p-value <0.002 with a critical Cox hazard ratio <0.78 for the primary outcome; and (ii) a critical Cox hazard ratio of <0.78 and a two-sided p-value <0.05 for the secondary outcome of time to cardiovascular death or ESKD [with 400 such events and a critical Cox hazard ratio of <0.78 a p-value of <0.014 would be observed]. Note the secondary outcome of time to cardiovascular death or ESKD is not part of the prospectively defined hypothesis testing strategy but is included as an additional stopping criterion to

ensure the trial is only stopped early if there is also substantial evidence of efficacy in this endpoint.

If these criteria are met, the key secondary outcomes will be formally analysed via the Hochberg procedure with the familywise error rate controlled at 3.0% (as per the Hwang-Shih-DeCani alpha-spending function, γ=0). If the trial is not stopped at the formal interim analysis, it will continue as planned until a minimum of 1070 participants have experienced the primary outcome, and the final two-sided p-value for the primary outcome would need to be <0.0497 to be deemed statistically significant. If statistically significant the Hochberg procedure with the familywise error rate controlled at 3.1% would be used for the key secondary outcomes. Note that if the proportion of first primary outcome events available at the interim analysis is not equal to 60% (information fraction used for both alpha-spending functions), then the p-values and critical hazard ratios at the interim/final analysis for the primary and key secondary outcomes will be adjusted accordingly (detailed separately, i.e. in the trial's DMC Charter). If this interim analysis is not considered appropriate (due to operational reasons, for example) then the alpha-level for the final analysis will be adjusted accordingly. No interim analysis for futility is planned.

2.6 CENTRAL AND REGIONAL COORDINATION OF LOCAL CLINICAL CENTRES

The Study will be coordinated by the CCO, based at the Medical Research Council Population Health Research Unit, which is part of the Clinical Trial Service Unit and Epidemiological Studies Unit (CTSU) at the University of Oxford. The CCO will oversee RCCs which will assist with selection of LCCs within their region and for the administrative support and monitoring of those LCCs. At each LCC, a Local Lead Investigator and LCC Research Coordinator (usually a qualified nurse, but in some cases may be medically qualified or have other relevant qualifications and experience) will be responsible for identification, recruitment, and follow-up (see Appendix 1: Organisational Structure and Responsibilities). It is intended that approximately 6000 participants will be randomized at about 200-250 LCCs worldwide.

2.6.1 Training and quality assurance

The study will be conducted in accordance with the principles of International Conference on Harmonisation Guidelines for Good Clinical Research Practice (ICH-GCP) and relevant local, national and international regulations (including the EU Clinical Trial Directive and the US Code of Federal Regulations Chapter 21). Prior to initiation of the study at any LCC, the RCC will confirm that the LCC has adequate facilities and resources to carry out the study (and, if considered necessary, a site visit will be undertaken). LLI and LCC Research Coordinators will be provided with materials detailing relevant study procedures and receive standardized training in study methods, including how to perform interviews, code using MedDRA, ensure appropriate levels of investigator support and oversight, and use the bespoke computer-based study management systems (see Section 2.6.3). Training will include information about empagliflozin, including the potential for ketoacidosis to present without excessive hyperglycaemia whilst treated with SGLT-2 inhibition. Full details are provided in the study-specific Training Standard Operating Procedure (SOP).

The study will use Quality-by-Design[39] approaches to prospectively build quality into the study design and operations rather than relying on retrospective monitoring. The focus will therefore be on those factors that are critical-to-quality (the protection of the participants and reliability of the trial results) rather than on the accuracy of individual data points. The Steering Committee will be responsible for reviewing study quality and risk-based management approaches and ensuring that the focus is always on issues that have (or the

potential to have) a substantial impact on the protection of the study participants or the reliability of the study results (with full details provided in a Quality Assurance Systems SOP).

Throughout the study, the CCO will centrally monitor performance against the predefined critical-to-quality factors. This process will predominantly be quantitative in nature and remedial actions will be determined based on the detection of deviations and totality of the evidence.

The relevant RCC and/or the CCO will arrange monitoring visits to LCCs as considered appropriate based on perceived training needs and the results of central process monitoring and statistical monitoring of study data (i.e. monitoring visits will be spaced by several months). The purpose of such visits will be to ensure that the study is conducted according to the protocol, ICH-GCP, and the applicable regulatory requirements, and by helping LCC clinic staff to resolve any local issues with the study and by providing additional focused training where necessary. Particular attention will be given to the effectiveness of strategies to recruit appropriate participants, the consenting process, the completeness of follow-up, the maintenance of participant compliance with the study treatments (which will be assessed by participant self-report), the reporting of study outcomes and reportable AEs (see Section 2.5.1), and collection of relevant supporting documentation to support the adjudication process (see Section 3.7). With the exception of local laboratory results (where a random subset will be assessed), no routine source data review and verification will take place as such data are obtained directly from participants (or occasionally from relatives or doctors) by interview. Where possible, monitoring visits will include observation of a participant's study visit. A report of each monitoring visit will be prepared by the study monitor and provided to LCC, RCC and CCO staff (including the Head of Monitoring) for review, and filed appropriately. Copies of these reports will be supplied to Boehringer Ingelheim on request. With prior arrangement, representatives of Boehringer Ingelheim may attend monitoring visits. Details of monitoring are provided in an On-Site Monitoring SOP.

2.6.2 Supply of study treatment

Study treatments will be manufactured, packaged, labelled and delivered to each LCC or RCC by Boehringer Ingelheim (or their subcontractor) under the direction of the CCO and according to Good Manufacturing Practices. An inventory of study drug supplies will be maintained on the study computer-based system and monitored at the CCO. LCC Local Investigators will be responsible for making appropriate arrangements for the storage and issuing of study treatments, and for the disposal of unused study drug in accordance with study SOPs.

2.6.3 Data management

All data in the study will be processed electronically using a set of custom-written applications developed to meet the requirements of the protocol and to comply with 21 CFR Part 11 and other relevant regulatory, legal and information security requirements. The LCC staff (usually the LCC Research Coordinator) will use bespoke web-based applications for local study management and to enter participant data (including study visit forms and AE information) directly into the database. These source data will be held in central databases located both at the CCO and at an independent third party where it will remain under the control of the Local Investigators (i.e. no paper case report forms exist). Any data queries reported by LCCs to the CCO or RCC (as per regional arrangements) during the study will be recorded onto the computer-based study management system. Data queries will be reviewed and managed by the CCO in accordance with an Internal Operating Procedure,

with data changes only made to pre-defined critical-to-quality data points. Clear electronic documentation will be maintained so the original data entry is not obscured and there is an audit trail for each entered data error and data change.

RCC and CCO staff will use the suite of administration applications on the computer-based system to manage LCCs and study participants, including central clinical supervision (review of AEs and laboratory results) by the CCO, management of follow-up and compliance, tracking of samples for central analysis, collection of supporting documentation for relevant events, and clinical outcome adjudication.

All data accesses will require a unique username and password, and any changes to data will require the user to enter their username and password as an electronic signature. Staff will have access restricted to only the functionality and data that are appropriate to their role in the study.

2.6.4 Biological sample assay, transport and storage

2.6.4.1 Local analysis of eligibility and safety bloods

Local laboratories will be used in all LCC study clinics for eligibility checks at the Screening visit (urine albumin:creatinine ratio [or protein:creatinine ratio, according to local practice], and blood creatinine plus liver transaminases [AST or ALT]), at the Randomization visit (blood creatinine, potassium, liver transaminases, bilirubin and haematocrit) and for clinical safety oversight at each follow-up visit (including blood creatinine, potassium and liver transaminases with bilirubin; see Section 4.2.1).

Haematocrit, haemoglobin, phosphate, sodium and corrected calcium will also be measured locally at 18 months of follow-up in a subset of about 20% (e.g. UK participants) of the surviving population.

2.6.4.2 Central assessment of samples collected at the randomization visit and during follow-up

Samples of both blood and urine are to be collected from all participants at the Randomization visit for central analysis and storage, including subsequent DNA extraction (subject to relevant consent, see Section 2.6.4.3) at a central ISO 17025 accredited laboratory. Central samples will not be used to assess eligibility. Further samples of both blood and urine are to be collected from all participants at the 2 month, 18 month (i.e. the approximate study midpoint) and Final Follow-up visits. Blood will be collected for central analysis of creatinine at the time of every scheduled Follow-up visit (see Section 4.2.1). RCCs will supply LCC staff with kits to collect these blood and urine samples. Blood is to be kept cool before centrifugation, separation into bar-coded cryovials, and storage at below -18°C within a day of the study clinic visit. Samples are to be transferred to below -40°C within 4 weeks. At appropriate intervals, samples will be collected from the LCCs (by the RCC or CCO) and transferred to the central laboratory for analysis (see Section 4.2.1) and for long-term frozen storage. Full details of sample collection, transport, storage and analysis are provided in a separate SOP.

2.6.4.3 Consent approval for unspecified analyses on blood and urine samples

Sample tubes will be labelled with a unique Sample ID which will be linked to the participant and the study visit using the study computer-based data entry system (i.e. samples will be pseudonymised). Outside the study clinic, staff involved in the transport, storage and analysis of these samples will have no means of linking tubes to an identifiable participant. Consent for protocol-specified analyses will be included in the main consent form. In addition, all participants will be asked if they would provide Supplementary Consent to allow

samples that have been collected for central laboratory analyses to be retained and used for unspecified analyses in the future. Similarly, Supplementary Consent will be sought to permit genetic material in the blood samples to be analysed. In all cases, participants will be free to opt in or out of any part of the Supplementary Consent without affecting their eligibility for the trial.

2.6.5 Administrative details

2.6.5.1 Source documents and archiving

Source documents for the study constitute the clinic visit records held in the study main database, results of protocol-mandated local laboratory blood and urine analyses, the additional information obtained on reported adverse events that are relevant to the outcome measures (see Section 3.7), death certificates, and drug supply records. These will be retained for at least 25 years from the completion of the study. Boehringer Ingelheim and regulatory agencies will have the right to commission a confidential audit of such records in the CCO, RCCs, and LCCs provided this does not result in unblinding while the study is in progress.

2.6.5.2 Funding

This study was initiated by CTSU, University of Oxford and developed as an academic collaboration with clinical scientists at Boehringer Ingelheim. Boehringer Ingelheim is the sponsor, and will perform regulatory submissions and interactions. It will also provide funding and packaged study medication (empagliflozin and matching placebo) for the study. Boehringer Ingelheim has delegated other roles to the University of Oxford, which is responsible for leading the trial scientifically and methodologically worldwide, including its conduct and statistical analysis. It is intended that the study will be conducted in the US in collaboration with independent scientists from the Duke Clinical Research Institute, Duke University. Data will be collected and analysed independently from the source of funding.

2.6.5.3 Indemnity

Boehringer Ingelheim will, at all times, indemnify the study investigators and study staff from claims that may be made against them for any injury sustained by a study participant as a consequence of participation in the study in accordance with this protocol. The indemnity will be outlined in detail in the agreements between the CCO, RCCs and LCCs (and in a letter from Boehringer Ingelheim).

2.6.5.4 End of the within-trial period

When the minimum number of required study outcomes has accrued (see Section 2.4.1.2), or the DMC advises the trial should be stopped early, participants will be invited to Final Follow-up visits. This visit may occur earlier than their planned next 6-monthly visit. The end of the trial is then defined as the latest of the following two dates: 7 days after the last participant's Final Follow-up visit, or the date of the last 4-week post-Final Follow-up blood draw.

2.6.5.5 Publications and reports

The Steering Committee (which includes representatives from University of Oxford, Duke University and Boehringer Ingelheim, as well as other individuals with relevant expertise) will be responsible for drafting the primary manuscript from the study and will establish a publication plan for secondary and supplementary analyses. In general, papers initiated by the Steering Committee (including the primary manuscript) will be written in the name of the Collaborative Group, with individual investigators named personally at the end of the report (or, to comply with journal requirements, in web-based material posted with the report). Draft copies of any manuscripts relating to the effects of empagliflozin from this trial will be

provided to Boehringer Ingelheim for review prior to publication but the decision to submit for publication will rest with the Steering Committee.

The Steering Committee will also institute a process by which proposals for additional publications (including from clinician scientists within Boehringer Ingelheim seeking to further evaluate the benefit-risk profile of empagliflozin and from independent external researchers) are considered by the Steering Committee before they begin. The Steering Committee will facilitate the use of the study data and approval will not be unreasonably withheld. However, the Steering Committee will need to be satisfied that any proposed publication is of high quality, honours the commitments made to the study participants in the consent documentation and ethical approvals, and is compliant with relevant legal and regulatory requirements (e.g. relating to data protection and privacy). The Steering Committee will have the right to review and comment on any draft manuscripts prior to publication.

2.6.5.6 Substudies

Proposals for substudies must be approved by the Steering Committee before they begin. In considering such proposals, the Steering Committee will need to be satisfied that the proposed substudy is of a high quality, and that it will not compromise the main study in any way (e.g. by reducing the recruitment rate or compliance with study treatment or overuse of stored biological samples).

FLOWCHART OF TRIAL ACTIVITIES

PRE-SCREENING PHASE

- Identify potentially eligible individuals: age ≥18 years*; chronic kidney disease (i.e. CKD-EPI eGFR ≥20, <45 mL/min/1.73m^2 or eGFR ≥45, <90 mL/min/1.73m^2 with urinary albumin: creatinine ratio ≥200 mg/g [or, if not available, urinary protein: creatinine ratio ≥300 mg/g])
- Invite to attend Screening visit clinic appointment in local study clinic

SCREENING VISIT (-8 to -12 WEEKS) AND PRE-RANDOMIZATION RUN-IN PHASE

- Relevant details to confirm participants meet certain inclusion criteria collected
- Written informed consent sought from potentially eligible and willing individuals
- Relevant medical history and medication to assess eligibility recorded
- Blood pressure measured and recorded
- Blood sample for creatinine, liver transaminases and urine sample for albumin:creatinine (or protein:creatinine) ratio taken for local laboratory analysis. Pregnancy test required only in women of child-bearing potential if, after questioning, pregnancy is considered reasonably possible.
- Eligible participants asked to start single-blind placebo Run-in phase
- Randomization visit appointment scheduled for 8-12 weeks later
- Local Research Co-ordinator enters local laboratory results and those ineligible dropped out
- Local Investigators review results, confirm individualized standard of care including appropriate dose of RAS-inhibition (where indicated and tolerated) and management of other relevant co-morbidities and approve participation

RANDOMIZATION VISIT (0 MONTHS)

- All SAEs during Run-in and full list of non-study medication recorded
- Blood pressure measured and recorded
- Check of compliance, eligibility and consent
- Assessment of other medical information (including history of peripheral arterial disease, amputation, heart failure, NYHA class & health related quality of life measured using the EQ5D-5L questionnaire)
- Height, weight, and hip and waist circumference recorded
- Blood samples taken for local laboratory analysis (creatinine/potassium/liver transaminases/bilirubin and haematocrit) and for central analyses (creatinine/HbA1c/NT-proBNP) and frozen storage
- Urine collected for central analysis (albumin and creatinine) and frozen storage
- Randomization via computer-based system: allocated empagliflozin 10 mg daily or matching placebo
- First Follow-up visit appointment scheduled for 2 months' time
- Participant's doctor informed of participant's randomization

FOLLOW-UP VISITS AT 2 and 6 MONTHS, THEN 6-MONTHLY

- SAEs, AESIs, selected non-serious AEs, adherence, and changes to non-study medication recorded
- Reasons for stopping study treatments and date of stopping recorded (where relevant)
- Blood pressure and weight measured and recorded (waist and hip circumference at 18 months and Final Follow-up)
- Blood sample taken for local laboratory analysis of creatinine, potassium, liver transaminases, and bilirubin at each visit in all participants and, in a 20% subset, haematocrit/ haemoglobin/ sodium/ phosphate/ corrected calcium at 18 months
- Central analysis for creatinine at each visit; HbA1c also measured in central samples in all participants at 2 and 18 months, and at the Final Follow-up (when scheduled study treatment ends)
- Urine samples taken for central analysis and storage for future assays in all participants at 2 and 18 months, and at the Final Follow-up
- Follow-up randomization treatment pack issued and assessment of compliance
- Assessment of quality of life (by EQ5D-5L questionnaire) at 18 months and Final Follow-up visit only
- Extra local blood sample taken 4 weeks after Final Follow-up for local laboratory analysis of creatinine in a 20% subset

OVERSIGHT AND MONITORING OF SAFETY AND EFFICACY

- LLI will meet regularly with LCC clinic staff to review study progress, including the delegation of duties log and approve listings of reported adverse events
- Central monitoring of laboratory results & adverse events by CCO clinicians (+ Early Recall if needed)
- Further details on relevant outcomes sought from participant's doctor and other sources (e.g. registries and electronic healthcare records) as required to support outcome adjudication
- Relevant events confirmed centrally by clinicians blind to treatment allocation

* Or "full age" as required by local regulation (e.g. 20 years in Japan)

3.1 ELIGIBILITY FOR THE STUDY

Consenting individuals are eligible for randomization if:

(i) Age is ≥18 years[c] at Screening;

(ii) There is evidence of chronic kidney disease at risk of kidney disease progression (see Section 3.1.1);

(iii) A local Investigator judges that the participant neither requires empagliflozin (or any other SGLT-2 or SGLT-1/2 inhibitor), nor that such treatment is inappropriate; and

(iv) None of the exclusion criteria apply (see Section 3.1.2).

Participants will be treated with appropriate doses of single agent RAS-inhibition with either ACEi or ARB unless such treatment is either not tolerated or not indicated (see Section 3.3.4).

No potential participant currently being treated with empagliflozin (or other SGLT-2 or SGLT-1/2 inhibitor) should be taken off this therapy to meet the eligibility criteria.

3.1.1 Inclusion criteria

Evidence of progressive CKD at risk of kidney disease progression is defined on the basis of local laboratory results recorded at least 3 months before and at the time of the Screening visit, and requires that:

(a) CKD-EPI eGFR ≥20 <45 mL/min/1.73m^2; or

(b) CKD-EPI eGFR ≥45 <90 mL/min/1.73m^2 with urinary albumin:creatinine ratio ≥200 mg/g (or protein:creatinine ratio ≥300 mg/g)

Note: the number of participants with or without diabetes mellitus (of any type) will be at least one-third of each, and the number of participants with an eGFR >45 mL/min/1.73m^2 limited to about one-third. The Steering Committee will monitor these proportions and will limit recruitment of particular categories of participant in whom sufficient numbers have already been screened or randomized.

3.1.2 Exclusion criteria

None of the following must be fulfilled:

(i) Currently receiving SGLT-2 or SGLT-1/2 inhibitor[d];

(ii) Diabetes mellitus type 2 and prior atherosclerotic cardiovascular disease[e] with an eGFR >60 mL/min/1.73m^2 at Screening;

(iii) Receiving combined ACEi and ARB[f] treatment[d];

(iv) Maintenance dialysis, functioning kidney transplant, or scheduled living donor transplant[d];

(v) Polycystic kidney disease;

(vi) Previous or scheduled bariatric surgery;

(vii) Ketoacidosis in the past 5 years;

(viii) Symptomatic hypotension[d], or systolic blood pressure <90 or >180 mmHg at Screening;

(ix) ALT or AST >3x ULN at Screening;

(x) Hypersensitivity to empagliflozin or other SGLT-2 inhibitor;

---

[c] Or “full age” as required by local regulation (e.g. 20 years in Japan).

[d] Based on self-reports at Screening and Randomization visits.

[e] Myocardial infarction, angina, stroke or peripheral arterial disease (including lower limb amputation)

[f] Or renin-inhibitor combined with ACEi or ARB.

(xi) Any intravenous immunosuppression therapy in last 3 months; or anyone currently on >45 mg prednisolone (or equivalent)[d];
(xii) Use of an investigational medicinal product in the 30 days prior to Screening visit;
(xiii) Known to be poorly compliant with clinic visits or prescribed medication[d];
(xiv) Medical history that might limit the individual's ability to take trial treatments for the duration of the study (e.g. severe respiratory disease; history of cancer or evidence of spread within last 4 years, other than non-melanoma skin cancer; or recent history of alcohol or substance misuse)[d];
(xv) Current pregnancy, lactation or women of childbearing potential (WOCBP), unless using highly-effective contraception[g];
(xvi) Type 1 diabetes mellitus.

In addition, individuals will be excluded at the Randomization visit if the participant:
(i) Does not adhere to Run-in treatment;
(ii) Is no longer willing to be randomized and followed for at least 3 years;
(iii) Is considered by a local investigator not to be suitable for randomization (see Section 3.3.4); or
(iv) Experiences ketoacidosis, heart attack, stroke, or hospitalization for heart failure, or hospitalization for urinary tract infection or acute kidney injury during Run-in.

Note that individuals who do not fulfil one or more inclusion criteria, or who fulfil one or more exclusion criteria, may be re-screened and later become eligible.

3.2 IDENTIFICATION AND INVITATION

3.2.1 Identification and invitation of potentially eligible participants

Extensive pre-screening efforts will be made to identify large numbers of potential participants at each LCC. The exact methods will vary by centre and by country, and in all cases will be subject to appropriate institutional review board approval and compliance with data privacy regulations. In general, potentially eligible participants (based on age, blood and urine results) will be identified from clinical records (including electronic health care records) and contacted to seek their provisional agreement to attend a Screening visit. Potential participants will be given information about the study.

3.3 SCREENING VISIT AND PRE-RANDOMIZATION RUN-IN

3.3.1 Assessment of relevant medical history and eligibility

LCC clinic staff will recheck basic inclusion criteria are met (e.g. age and the blood/urine results used to identify potential participants) then take written informed consent.

3.3.2 Written consent

Individuals who appear initially to be eligible will have the study explained to them by the clinic staff, using the Participant Information Leaflet and Consent Form as a basis for discussion. Where relevant, supplementary Consent will also be sought (see Section 2.6.4.3). Each individual will have an opportunity to initiate discussion, and have time to think about their participation in the study, perhaps after discussing it with their family or a local doctor. Individuals who choose to do this will be asked to attend a repeat Screening visit

[d] Based on self-reports at Screening and Randomization visits.
[g] Highly effective methods of contraception include implants, injectables, combined oral contraceptives (the participant must have been on a stable dose for at least 3 months prior to entering the trial), intrauterine device, vasectomised partner, or true sexual abstinence (when this is the preferred and usual lifestyle of the patient and does not include periodic abstinence [e.g. calendar, ovulation, symptothermal or post-ovulation methods]). Use of such methods must be maintained throughout the trial and for 7 days after the end of the trial.

within a few weeks. Attendees will be discouraged from participating if it is thought unlikely that they would be willing and able to continue attending Follow-up visits for at least 3 years.

3.3.3 Confirmation of eligibility and collection of blood and urine samples

After providing written consent, blood/urine test results will be recorded. Other medical history (including primary renal diagnosis and other co-morbidity), non-study medication, blood pressure and other factors pertinent to eligibility will be obtained directly from participants (rather than from hospital record review) and recorded directly into the Screening Form. These inclusion and exclusion criteria will be checked with the assistance of the study computer-based system.

A non-fasting blood sample will be taken for local analysis of creatinine and liver transaminases (AST or ALT), and a urine sample will be taken for local analysis of urinary albumin:creatinine ratio (or, if albuminuria measurement unavailable in local laboratory, protein:creatinine ratio). The LCC clinic staff will issue a 15 week supply of placebo tablets to eligible participants.

WOCBP, defined as women less than 55 years of age unless surgically sterile or with history of a postmenopausal state, will be requested to use highly effective methods of contraception. A pregnancy test will be offered if, after questioning about recent menses and regularity of menstrual cycle, pregnancy is considered reasonably possible (or if a pregnancy test is required by local regulation).

Participants with diabetes will be educated about the risks of diabetic ketoacidosis and the actions they should take if they suspect it, and will be provided with a specific information card (see Section 3.5.6).

An appointment will be made for the Randomization visit in 8-12 weeks. The participant's doctor(s) will be informed that the participant has entered Run-in.

Following the Screening visit, the locally analysed blood and urine results will be recorded onto the study computer-based system, which will provide another assessment of eligibility (see Section 3.1). If these results indicate that the participant is not eligible for the trial, they may be repeated once if in the opinion of a Local Investigator they were spurious, otherwise the participant will be withdrawn from the Run-in period and asked to stop and return all placebo Run-in medication.

3.3.4 Review of eligibility and renin-angiotensin system inhibition by a Local Investigator

During Run-in, the LLI (or authorised delegate) will be given a description of the participant's medical history (including primary renal diagnosis), single agent RAS-inhibition treatment and blood and urine results all based on the Screening visit, and asked to indicate whether, in their view, these results (or any other factor) make the participant unsuitable for entry into the randomized phase of the study. Additionally, participants should be randomized only if a Local Investigator judges that the participant does not require empagliflozin (or any other SGLT-2 or SGLT-1/2 inhibitor), and neither is such treatment inappropriate. No patient currently being treated with empagliflozin (or other SGLT-2 or SGLT-1/2 inhibitor) should be taken off this therapy to meet the eligibility criteria.

For eligible participants, the LLI (or authorized and medically qualified delegate) will also be asked to confirm that the participant is prescribed, in their opinion, an appropriate dose of

single agent RAS-inhibition (i.e. ACEi or ARB, but not both). Those participants for whom RAS inhibition is not considered indicated (e.g. due to concomitant medication or co-morbidity), or who cannot tolerate RAS inhibition, will still be eligible to enter the trial, but the reason for not using RAS-inhibition will be documented. Those participants who, in the opinion of the LLI, need to start RAS inhibition or are not on an appropriate dose will be excluded from the study (but may be rescreened later, e.g. once established on an appropriate dose).

Additionally, throughout the study, the care of participants will remain the responsibility of their local doctors, who are asked to ensure appropriate and individualized care. This includes appropriate management of risk of kidney disease progression, risk of cardiovascular disease, and other conditions which are common in CKD (such as mineral-bone disorder, renal anaemia, metabolic acidosis). Modifiable risk factors include but are not limited to glycaemic control in participants with diabetes, blood pressure control, and treatment of dyslipidaemia. It is advised that this is conducted in the context of prevailing local, national or international guidance.

3.4 Randomization Visit (0 months)

3.4.1 Final check of eligibility and compliance before randomization

For individuals who attend their Randomization Clinic appointment, study eligibility will be confirmed (see Section 3.1). The participant will also be asked if they have experienced any SAE or significant problems during the Run-in period. Information on other relevant factors will also be collected, such as prior history of urosepsis, heart failure and New York Heart Association (NYHA) functional classification, history of peripheral neuropathy, diabetic foot ulcer, lower limb infection or gangrene, smoking history and alcohol intake, and an assessment of health related quality of life is to be made (using the EQ5D-5L questionnaire). Details of all non-study treatments will be sought, compliance with Run-in treatment checked, and consent information checked. Blood pressure, height, weight, and hip and waist circumference will be measured. The participant's willingness to take study medication and attend follow-up visits for at least 3 years will be confirmed. Details will be recorded directly onto the Randomization Form on the study computer-based system (which is designed to obtain complete information, assess eligibility, and to prompt appropriate actions).

3.4.1.1 Collection of blood and urine samples

Eligible participants will have a blood sample taken for local measurement of creatinine, potassium, liver function (ALT or AST, and bilirubin), and haemoglobin/haematocrit. Blood and urine samples will also be processed in preparation for subsequent transportation to the central laboratory (see Section 2.6.4).

3.4.2 Random allocation of study treatment

Eligible and consenting individuals will be allocated empagliflozin or matching placebo using a minimized randomization program on the study computer-based system (see Section 2.2.2).[33] Participants will be allocated a numbered treatment pack containing a 7-month supply of one tablet daily of either active empagliflozin 10 mg or matching placebo.

The numbered treatment packs will be issued to the participant by the LCC clinic staff or their local hospital pharmacy. An appointment for the first post-randomization Follow-up visit will then be made by the study staff, with guidance from the study computer-based system. The participant's doctor(s) will be informed that the participant has been randomized. Following the Randomization visit, the locally analysed blood results will be recorded onto the study computer-based system by the LCC.

3.5 Follow-up Visits (2 and 6 months and then 6-monthly)

3.5.1 Recording adverse events and adherence to study treatment

Following randomization, all participants are scheduled to attend Follow-up visits at 2 and 6 months, and then 6-monthly until the end of the study.

At each visit, details of all hospital admissions and any other SAEs will be sought from participants, and questions will specifically be asked about SAEs due to urinary tract infection, genital infection, hyperkalaemia, acute kidney injury and dehydration. Information about new-onset of diabetes, gout, AESIs (i.e. liver injury, ketoacidosis and lower limb amputation), bone fractures, severe hypoglycaemia and symptomatic dehydration will also be recorded. The source of the information/data is the report from the participant (or where unavailable a relative or other doctor) entered directly into the electronic case report form. In this study, paper or electronic hospital notes are not routinely reviewed to identify AEs. Any SAE considered to be due to study treatment (i.e. a possible SSAR) is to be discussed as soon as possible with a RCC/CCO study clinician in order that additional information can be collected (see Section 2.5.1.3).

Key changes to non-study medication will be sought, and adherence to study treatment will be reviewed. Adherence will be assessed and documented by LCC clinic staff at every study visit by asking participants about missed doses and visual inspection of remaining tablets (a pill count will not be performed). The amount of study treatment taken since the last visit will be estimated and recorded as “most”, “some” or “little/none” (with further guidance on these categories provided in the LCC Clinic Manual). The LCC clinic staff will discuss any reasons for non-adherence with the participant (and with their LLI or with CCO clinical staff if necessary) and encourage the participant to take study treatment regularly whenever appropriate. For participants who discontinue study treatment, the reason for doing so will be sought.

Blood pressure and weight will be measured at each Follow-up visit. At the 18 month and the Final Follow-up visit, hip and waist circumference will be measured and health related quality of life (using EQ5D-5L questionnaire) will be assessed. Details are to be recorded directly onto the electronic Follow-up form on the study computer-based system.

Local Research Co-ordinators will be trained to ask participants to report any relevant AEs (related or not) occurring up to 7 days after their Final Follow-Up Visit directly to LCC clinic staff.

3.5.2 Collection of blood and urine samples

At each Follow-up visit, a non-fasting blood sample will be taken for local analysis for creatinine, potassium and liver function (ALT or AST, and bilirubin). Results are ideally entered onto the computer-based system within 2 working days. At 18 months of follow-up, a 20% subset of participants (e.g. UK participants) will also have haematocrit, haemoglobin, phosphate, sodium and corrected calcium measured locally.

Four weeks after the Final Follow-up visit, a subset of about 20% of participants (who have not started dialysis or have a functioning kidney transplant) will provide a further non-fasting blood sample for local analysis of creatinine (known as the 4-week post-Final Follow-up blood draw) and a urine sample for local analysis of urine albumin and creatinine.

At each scheduled follow-up visit a central blood sample will be collected. At the 2 and 18 month and Final Follow-up visits, urine samples will also be collected (see Section 2.6.4.2).

3.5.3 Issuing study treatment and arranging further appointments

Provided continuing study treatment remains appropriate, participants will be given a further 7-month supply of their randomly allocated study treatment (empagliflozin 10 mg or matching placebo), and any previously allocated treatment will be retrieved (except at the 2-month visit). An appointment will then be made for their next scheduled Follow-up visit.

3.5.4 Follow-up for randomized participants not attending study clinics

Follow-up information is to be collected from all study participants, irrespective of whether they continue to take study treatment, usually at routine Follow-up clinic visits, unless they withdraw consent (see Section 3.6.5). If, however, a participant becomes unwilling or unable to attend study clinic visits then LCC staff will telephone the participant (or, where appropriate, their relative or carer) at the time of each of their scheduled Follow-up appointments and complete the necessary Follow-up form on the study computer-based system. If monitoring of blood is no longer possible (e.g. because the participant no longer attends clinic visits and no other means of measuring creatinine/liver function can be arranged), then the participant will be asked to discontinue all study treatment and advised to see a local doctor. All efforts will be made to continue to follow-up such participants (as described above), and those being followed by telephone or other remote method will be encouraged to provide blood samples for central analysis at relevant time points. If this is not possible, then LCC or RCC staff will attempt to check a participant’s progress by interview or direct correspondence with the participant’s own local doctors or (where appropriate consent and approvals are in place) by reviewing available information on routine healthcare systems (including local blood result systems) and registries. (In the UK, for example, there are registries for treated ESKD, hospital admissions, cancers, and deaths.) Such information could also be used for long-term follow-up, alongside participant questionnaires administered by telephone, mail or electronically.

3.5.5 Monitoring of women of child bearing potential

Animal studies show that empagliflozin crosses the placenta during late gestation to a very limited extent, but it is considered preferable to avoid its use during pregnancy. WOCBP will therefore have to agree to use highly effective methods of contraception during the trial. At each visit, a pregnancy test will be offered if, after questioning about recent menses and regularity of menstrual cycle, pregnancy is considered reasonably possible (or if a pregnancy test is required by local regulation). LCC clinic staff will also reinforce the need for highly effective contraception at each visit. If a participant becomes pregnant during the trial, the trial medication will be stopped and the participant will be followed up until birth or termination of the pregnancy (see further details for reporting of pregnancy in Section 2.5.1.1).

3.5.6 Monitoring of people with diabetes mellitus

LCC clinic staff will receive training on the specific risk of ketoacidosis (which can present with lower than anticipated blood glucose levels in people with diabetes treated with SGLT-2 inhibitors) and will be asked to provide additional written information about ketoacidosis to participants with diabetes (e.g. a trial information card). Testing equipment and materials to detect blood ketones will be available to people with type 1 diabetes before Randomization.

3.5.7 Local Lead Investigator supervision

LLI will meet regularly with the other LCC staff to review study progress, the delegation of duties log and approve listings of reported adverse events. The RCC or CCO will be contacted if adverse event information needs to be refined.

3.6 Central Monitoring of Participant Safety, Early Recall Visits and Modifying Study Treatment

3.6.1 Early Recall Visits

An Early Recall visit may be arranged for any participant who requires review outside their planned visit schedule. Examples of circumstances where this may be necessary include the assessment of abnormal values in safety blood results from routine Follow-up visits, or if symptoms of liver disease (e.g. icterus) develop between scheduled Follow-up visits, or an extra visit is required a few weeks after the Randomization Visit (e.g. if requested by local regulators or Local Investigator). As at routine study visits, the results of blood tests performed at Early Recall visits will be entered by LCC clinic staff into the study computer-based system (which is designed to prompt appropriate actions) and these results will be monitored centrally by clinical staff at the CCO and RCCs in accordance with the study procedures.

3.6.2 Monitoring liver function, potassium, creatinine and AESIs

CCO study clinicians will be responsible for reviewing local results on liver function, potassium and creatinine, and all reports of AESIs. They will advise on the need for (and timing of) Early Recall visits and whether study treatment should be stopped or restarted. In so doing, CCO study clinicians will collaborate with the LLI and other LCC clinic staff (or RCC in certain regions), as necessary, and will generally initiate contact if there have been results that fulfil the definition of liver injury or a 50% increase in creatinine since the preceding Follow-up visit. Management strategies include ascertainment of a more detailed clinical picture, additional investigations, more frequent study visits, or a lower threshold for stopping study treatment.

3.6.3 Modifying study treatment

If adverse events occur that are believed to be due to empagliflozin, including significant elevation of liver transaminases, the study treatments may be temporarily or permanently discontinued. The following events are also sufficient reason to discontinue the study empagliflozin or placebo:

- SAE considered likely to be due to the study treatment (i.e. SSAR, see Section 2.5.1.3)
- Kidney transplantation
- New reason to prescribe empagliflozin or another SGLT-2 or SGLT-1/2 inhibitor (e.g. a local doctor of the opinion that it should be included as part of the current standard of care for prevention of cardiovascular events); or new reason not to use an SGLT-2 inhibitor (e.g. local doctors may choose to stop study treatment on initiation of maintenance dialysis or after ketoacidosis)
- Pregnancy or suspected pregnancy
- At the request of the participant or their doctors (for whatever reason) or any other situation where continuing study treatment is not considered to be in the participant’s best interests by their own doctors or the study clinical team (including cessation of use of reliable contraception in WOCBP or use of high potency immunosuppression).

Whenever possible, the study computer-based system will prompt LCC clinic staff to consider whether there are specific reasons to discontinue or to restart study treatment (if appropriate, i.e. if reasons for discontinuation do not exist any longer). CCO study clinicians will provide advice if required.

3.6.4 Unblinding of study treatment

There are two main situations in which unblinding of the treatment allocation (empagliflozin or placebo) for an individual participant may be warranted:

- When knowledge of the treatment allocation could materially influence the immediate medical management (e.g. after overdose)
- When unblinding is necessary as part of Safety Reporting (see Section 2.5.1.4).

Urgent unblinding is available by contacting a CCO study clinician on a 24-hour basis via the CTSU Freefone telephone service. For the avoidance of doubt, if an investigator or local doctor requests the unblinded treatment allocation, it will be provided. All unblinding episodes are logged within the study computer-based system.

3.6.5 Withdrawal of consent

Participants may decide that they no longer wish to take study treatment or are no longer willing to attend study visits. LCCs may be able to help participants overcome problems associated with personal circumstances (e.g. provide transport support to attend clinics). These decisions are not considered to be withdrawals of consent, and appropriate procedures for dealing with them are described elsewhere in this protocol (e.g. for discontinuation of study treatment see Section 3.6.3 and for alternative methods of follow-up see Section 3.5.4). However, participants are free to withdraw consent for some or all aspects of the study at any time. In order to ensure that relevant safeguards are put in place to maintain the individual’s safety (e.g. if an important safety issue comes to light that might affect a participant who has previously withdrawn from the study) and to prevent a breach of the individual’s decision to withdraw (e.g. to prevent re-invitation of an individual who had previously withdrawn consent), the decision to withdraw should ideally be put in writing and a copy maintained at the LCC (with key data items being recorded on the study computer-based system). This written information should specify which aspect(s) of the study consent is being withdrawn: for example, direct contact from study staff; collection of information from a relative or friend; collection of information from local doctors or routine data sources; or the storage and analysis of samples for protocol-specified future unspecified assays. (In accordance with regulatory guidance, data that have already been collected and incorporated into the study database, including the results of laboratory assays, will continue to be processed.)

3.7 CONFIRMATION AND VERIFICATION OF STUDY OUTCOMES (“ADJUDICATION”)

Outcomes (and components) purely based on laboratory values (e.g. sustained ≥40% decline in eGFR,) will not be adjudicated and analyses will emphasize the results of measurements made at central laboratories. Wherever possible, eGFR will be calculated using centrally measured serum creatinine (with local results substituted if central results are unavailable). eGFR will be calculated using the CKD-EPI creatinine formula.[40]

Receipt of a kidney transplant or initiation of maintenance dialysis will also not be adjudicated. Instead, LCC reports will be cross-checked by the CCO with information at

subsequent follow-up visits or (where available) with additional medical information collected for adjudication of deaths.

Additional medical information will be sought for AEs that undergo adjudication. In general, these will be limited to all deaths and events initially reported as hospitalization for heart failure, myocardial infarction, stroke, liver injury, ketoacidosis, lower limb amputation and acute kidney injury. Other events may be added to the adjudication list if considered necessary to ensure a reliable assessment of the clinical effects (and particularly safety) of empagliflozin. Most hospitalizations will not be adjudicated and so analyses of hospitalizations will mainly be based on LCC reports.

Relevant information needed for adjudication may come from the records held at the LCCs and other hospitals, from participant's own doctors, or from electronic sources and registries. In some cases it may be necessary to obtain information that predates randomization into the study. A central panel of clinicians based at, or overseen by, the CCO will provide the adjudication. Review, processing and adjudication of AEs will be conducted in accordance with the study SOPs and will be blinded to study treatment allocation (empagliflozin or placebo). The relevant SOP will detail a quality control process where the first events adjudicated by each adjudicator and a random subsample thereafter (about 5%) will be reviewed by a second adjudicator (blind to original adjudication).

3.8 CITED REFERENCES

1. Levey AS, Coresh J. Chronic kidney disease. *Lancet* 2012; **379**(9811): 165-80.

2. Stevens PE, O'Donoghue DJ, de Lusignan S, et al. Chronic kidney disease management in the United Kingdom: NEOERICA project results. *Kidney Int* 2007; **72**(1): 92-9.

3. Matsushita K, van der Velde M, Astor BC, et al. Association of estimated glomerular filtration rate and albuminuria with all-cause and cardiovascular mortality in general population cohorts: a collaborative meta-analysis. *Lancet* 2010; **375**(9731): 2073-81.

4. Go AS, Chertow GM, Fan D, McCulloch CE, Hsu CY. Chronic kidney disease and the risks of death, cardiovascular events, and hospitalization. *N Engl J Med* 2004; **351**(13): 1296-305.

5. Cholesterol Treatment Trialists Collaboration. Impact of renal function on the effects of LDL cholesterol lowering with statin-based regimens: a meta-analysis of individual participant data from 28 randomised trials. *The lancet Diabetes & endocrinology* 2016; **4**(10): 829-39.

6. Ninomiya T, Perkovic V, Turnbull F, et al. Blood pressure lowering and major cardiovascular events in people with and without chronic kidney disease: meta-analysis of randomised controlled trials. *BMJ* 2013; **347**: f5680.

7. Park M, Hsu CY, Li Y, et al. Associations between kidney function and subclinical cardiac abnormalities in CKD. *J Am Soc Nephrol* 2012; **23**(10): 1725-34.

8. Foley RN, Parfrey PS, Kent GM, Harnett JD, Murray DC, Barre PE. Long-term evolution of cardiomyopathy in dialysis patients. *Kidney Int* 1998; **54**(5): 1720-5.

9. Zinman B, Wanner C, Lachin JM, et al. Empagliflozin, Cardiovascular Outcomes, and Mortality in Type 2 Diabetes. *N Engl J Med* 2015; **373**(22): 2117-28.

10. Fox CS, Matsushita K, Woodward M, et al. Associations of kidney disease measures with mortality and end-stage renal disease in individuals with and without diabetes: a meta-analysis. *Lancet* 2012; **380**(9854): 1662-73.

11. Jafar TH, Schmid CH, Landa M, et al. Angiotensin-converting enzyme inhibitors and progression of nondiabetic renal disease. A meta-analysis of patient-level data. *Ann Intern Med* 2001; **135**(2): 73-87.

12. Lewis EJ, Hunsicker LG, Clarke WR, et al. Renoprotective effect of the angiotensin-receptor antagonist irbesartan in patients with nephropathy due to type 2 diabetes. *N Engl J Med* 2001; **345**(12): 851-60.

13. Brenner BM, Cooper ME, de Zeeuw D, et al. Effects of losartan on renal and cardiovascular outcomes in patients with type 2 diabetes and nephropathy. *N Engl J Med* 2001; **345**(12): 861-9.

14. Fried LF, Emanuele N, Zhang JH, et al. Combined angiotensin inhibition for the treatment of diabetic nephropathy. *N Engl J Med* 2013; **369**(20): 1892-903.

15. Wanner C, Inzucchi SE, Lachin JM, et al. Empagliflozin and Progression of Kidney Disease in Type 2 Diabetes. *N Engl J Med* 2016; **375**(4): 323-34.

16. Schnaper HW. Remnant nephron physiology and the progression of chronic kidney disease. *Pediatric nephrology* 2014; **29**(2): 193-202.

17. Ruggenenti P, Porrini EL, Gaspari F, et al. Glomerular hyperfiltration and renal disease progression in type 2 diabetes. *Diabetes Care* 2012; **35**(10): 2061-8.

18. Tonneijck L, Muskiet MH, Smits MM, et al. Glomerular Hyperfiltration in Diabetes: Mechanisms, Clinical Significance, and Treatment. *J Am Soc Nephrol* 2017; **28**(4): 1023-39.

19. Haynes R, Staplin N, Emberson J, et al. Evaluating the contribution of the cause of kidney disease to prognosis in CKD: results from the Study of Heart and Renal Protection (SHARP). *Am J Kidney Dis* 2014; **64**(1): 40-8.

20. Jha V, Garcia-Garcia G, Iseki K, et al. Chronic kidney disease: global dimension and perspectives. *Lancet* 2013; **382**(9888): 260-72.

21. USRDS. International Comparison. 2014 Annual Data Report: Atlas of End-Stage Renal Disease in the United States. , National Institutes of Health, National Institute of Diabetes and Digestive and Kidney Diseases, Bethesda, MD. 2014; **2**.

22. Ridderstrale M, Andersen KR, Zeller C, et al. Comparison of empagliflozin and glimepiride as add-on to metformin in patients with type 2 diabetes: a 104-week randomised, active-controlled, double-blind, phase 3 trial. *The lancet Diabetes & endocrinology* 2014; **2**(9): 691-700.

23. Haring HU, Merker L, Seewaldt-Becker E, et al. Empagliflozin as add-on to metformin in patients with type 2 diabetes: a 24-week, randomized, double-blind, placebo-controlled trial. *Diabetes Care* 2014; **37**(6): 1650-9.

24. Haring HU, Merker L, Seewaldt-Becker E, et al. Empagliflozin as add-on to metformin plus sulfonylurea in patients with type 2 diabetes: a 24-week, randomized, double-blind, placebo-controlled trial. *Diabetes Care* 2013; **36**(11): 3396-404.

25. Kovacs CS, Seshiah V, Swallow R, et al. Empagliflozin improves glycaemic and weight control as add-on therapy to pioglitazone or pioglitazone plus metformin in patients with type 2 diabetes: a 24-week, randomized, placebo-controlled trial. *Diabetes Obes Metab* 2014; **16**(2): 147-58.

26. Roden M, Weng J, Eilbracht J, et al. Empagliflozin monotherapy with sitagliptin as an active comparator in patients with type 2 diabetes: a randomised, double-blind, placebo-controlled, phase 3 trial. *The lancet Diabetes & endocrinology* 2013; **1**(3): 208-19.

27. Rosenstock J, Jelaska A, Frappin G, et al. Improved glucose control with weight loss, lower insulin doses, and no increased hypoglycemia with empagliflozin added to titrated multiple daily injections of insulin in obese inadequately controlled type 2 diabetes. *Diabetes care* 2014; **37**(7): 1815-23.

28. Rosenstock J, Jelaska A, Zeller C, et al. Impact of empagliflozin added on to basal insulin in type 2 diabetes inadequately controlled on basal insulin: a 78-week randomized, double-blind, placebo-controlled trial. *Diabetes Obes Metab* 2015; **17**(10): 936-48.

29. Tikkanen I, Narko K, Zeller C, et al. Empagliflozin reduces blood pressure in patients with type 2 diabetes and hypertension. *Diabetes Care* 2015; **38**(3): 420-8.

30. Barnett AH, Mithal A, Manassie J, et al. Efficacy and safety of empagliflozin added to existing antidiabetes treatment in patients with type 2 diabetes and chronic kidney disease: a randomised, double-blind, placebo-controlled trial. *The lancet Diabetes & endocrinology* 2014; **2**(5): 369-84.

31. Kohler S, Zeller C, Iliev H, Kaspers S. Safety and Tolerability of Empagliflozin in Patients with Type 2 Diabetes: Pooled Analysis of Phase I-III Clinical Trials. *Advances in therapy* 2017; **34**(7): 1707-26.

32. Neal B, Perkovic V, Mahaffey KW, et al. Canagliflozin and Cardiovascular and Renal Events in Type 2 Diabetes. *N Engl J Med* 2017; **377**(7): 644-57.

33. Pocock SJ, Simon R. Sequential treatment assignment with balancing for prognostic factors in the controlled clinical trial. *Biometrics* 1975; **31**(1): 103-15.

34. Peto R, Pike MC, Armitage P, et al. Design and analysis of randomized clinical trials requiring prolonged observation of each patient. II. analysis and examples. *Br J Cancer* 1977; **35**(1): 1-39.

35. U.S. Department of Health and Human Services Food and Drug Administration. Guidance for Industry Determining the Extent of Safety Data Collection Needed in Late Stage Premarket and Postapproval Clinical Investigations. 2015.

36. U.S. Department of Health and Human Services Food and Drug Administration. Guidance for Industry. Safety Assessment for IND Reporting. Dec 2015.

37. ICH Harmonised tripartite guideline Clinical safety data management: definitions and standards for expedited reporting E2A. Oct 1994.

38. Risk proportionate approaches in clinical trials. Recommendations of the expert group on clinical trials for the implementation of Regulation (EU) No 536/2014 on clinical trials on medicinal products for human use. Apr 2017.

39. Meeker-O'Connell A, Glessner C, Behm M, et al. Enhancing clinical evidence by proactively building quality into clinical trials. *Clinical trials* 2016; **13**(4): 439-44.

40. Levey AS, Stevens LA, Schmid CH, et al. A new equation to estimate glomerular filtration rate. *Ann Intern Med* 2009; **150**(9): 604-12.

4.1 APPENDIX 1: ORGANISATIONAL STRUCTURE AND RESPONSIBILITIES FOR STUDY DESIGN AND CONDUCT

Boehringer Ingelheim

Boehringer Ingelheim has delegated certain responsibilities to the independent Principal Investigators and CCO at the University of Oxford, and to the Steering Committee. Boehringer Ingelheim remains fully responsible for:

- Provision of study funding
- Provision and distribution (but not allocation) of manufactured and labelled study drug
- Regulatory submissions and interactions (with support from the Principal Investigators)
- Auditing of investigational sites, facilities, study setup, study processes, etc. as per the Audit Plan.

Responsibility for oversight of the quality and integrity of the trial data remains with Boehringer Ingelheim.

Principal Investigators

The Principal Investigators have overall responsibility for:

- Design of the study (in collaboration with Boehringer Ingelheim)
- Preparation of the Protocol and subsequent revisions
- Managing the CCO
- Development of computer-based systems and study SOPs.

Steering Committee

The Steering Committee is responsible for:

- Agreeing the Data Analysis Plans
- Reviewing progress of the study and, if necessary, suggesting and agreeing changes to the Protocol
- Reviewing new scientific evidence that may be of relevance
- Drafting, review and approval of study main publication(s)
- Review and approval of proposals for subsequent analyses and publications
- Approval of substudy proposals
- Reviewing study quality and risk management approaches, and ensuring that the focus is always on issues that have (or the potential to have) a substantial impact on the safety of the study participants or the reliability of the study results
- Monitoring participant characteristics and limiting recruitment of particular categories of participant when sufficient numbers have already entered the trial.

Data Monitoring Committee

The independent Data Monitoring Committee is responsible for:

- Reviewing unblinded interim data according to the schedule outlined in the Protocol
- Advising the Steering Committee if, in their view, the randomized data provide evidence that may warrant early termination of all or part of the study for either efficacy or safety

- Review of the formal interim analyses (see Section 2.5.2.2).

Central Coordinating Office
The CCO is responsible for the overall coordination of the Study, including:

- Study planning and organisation of Steering Committee meetings
- Agreement of each regional recruitment plan (including countries, number of LCCs, number of participants, and timelines)
- Contractual issues with RCCs and budget administration
- Co-ordination of Ethics Committee applications
- Supporting Boehringer Ingelheim in their interactions with regulatory authorities and other outside agencies as appropriate
- Design, implementation and maintenance of computer-based systems for the study (including CCO/RCC computer-based system for administration and study computer-based system for direct data entry)
- Provision of study materials to RCCs and LCCs, and provision of IT support to RCCs
- Monitoring of drug supply in liaison with Boehringer Ingelheim (who will be responsible for drug distribution to each LCC)
- Central laboratory assay and long-term storage of blood and urine samples
- Monitoring of overall progress of the study, with a focus on critical-to-quality factors
- Clinical safety monitoring, including reporting of SSARs to the Chair of the Data Monitoring Committee and to Boehringer Ingelheim
- Responding to technical, medical, data and administrative queries from the RCCs
- Manage data queries and data changes (with a clear audit trail)
- Management of outcome adjudication
- Liaison with the Data Monitoring Committee.

Regional Coordinating Centres
Each RCC is responsible, under the direction of its Regional Coordinator, for:

- Identification of potential LCCs and agreement of their recruitment plans (including number of participants and timelines)
- Contractual issues with LCCs and regional budget administration
- Obtaining any central Ethics Committee approval (where appropriate) and assisting LCCs with local Ethics Committee applications
- Training of LCC staff and assistants
- Distribution of study materials to LCCs
- Responding to technical, medical and administrative queries from the LCCs
- Perform LCC on-site monitoring visits by trained study monitors
- “Process” monitoring of LCCs by responding to regular or occasional reports on regional progress prepared by the CCO
- Ensuring appropriate follow-up of abnormal safety blood results
- Collection and initial processing of relevant documentation to confirm reported events in line with study SOPs
- Collection and short-term storage of blood and urine samples from LCCs, and subsequent transport of them to the CCO
- Organisation of meetings of collaborators within the region
- Entering data entry errors reported by LCCs
- Supporting Boehringer Ingelheim in their interactions with regulatory authorities as appropriate

- Ensuring that trial-related activities are performed according to local regulations.

Local Clinical Centres
The LCC lead investigator and LCC staff are responsible for:

- Meeting regularly as a team to review study progress locally, the delegation of duties log and for Local Investigators to review and approve listings of locally reported adverse events (i.e. provide LLI oversight)
- Obtaining local Ethics Committee approval were necessary (aided by the RCC)
- Obtaining local management approval where necessary
- Performing trial-related activities according to local regulations
- Provision of adequate clinic space and access to appropriate systems for the identification of potentially eligible individuals
- Conducting clinic procedures: managing and distributing study drugs (in conjunction, if required, with the hospital pharmacy), and maintaining relevant study equipment in accordance with the Protocol and SOPs
- Ensuring adequate local laboratory facilities for safety monitoring and, if necessary, processing and temporarily storing samples for central analysis
- Reviewing Screening Form data, confirm appropriate dose of RAS-inhibition (where relevant), confirm no reasons to prescribe or not prescribe empagliflozin (or other SGLT-2 or SGLT-1/-2 inhibitor), and approving participants for randomization (Local Investigators only)
- Providing individualized care, including management of cardiovascular risk factors and other existing comorbidities (e.g. hypertension, diabetes) according to relevant guidelines
- Dealing with routine enquiries from participants and their families in collaboration with the RCC where necessary
- Obtaining clinical information when requested to confirm potential primary and secondary, tertiary and safety study outcomes
- Informing the RCC or CCO of any possible data entry errors.

4.2 APPENDIX 2: VISIT SCHEDULE AND PROCEDURES

4.2.1 Clinic procedures

Task	Activity	Registration	Screening	Randomization			In-trial follow-up	
			- 12 to -8 weeks	0 months	2 months	6 months	6 monthly visits	Final visit**
Demographics	Record contact details	X	check	check	check	check	check	check
Medical history & eligibility assessment	Relevant medical history (incl. inclusion criteria & an dose of RAS inhibitor)		X	X				
	Exclusion criteria (incl. relevant non-study medication)		X	X				
	Other information (smoking, alcohol)			X				
	Use of non-study medication		X	X	X	X	X	X
Consent	Obtain consent		X					
	Confirm consent			X				
Safety & outcomes reporting	Adverse events (incl. Suspected Serious Adverse Reactions)			X	X	X	X	X
	Self-reported compliance			X	X	X	X	X
	Reasons for stopping study treatment (incl. SAEs, non-serious adverse events, other reasons)			X	X	X	X	X
	Remote follow-up using routine data sources and/or participant surveys*				(X)*	(X)*	(X)*	(X)*
	Quality of life (by EQ5D-5L)			X			18 month only	X
Physical measurements	Blood pressure		X	X	X	X	X	X
	Height			X				
	Weight			X	X	X	X	X
	Hip & waist circumference			X			18 month only	X
Local laboratory assessments	Creatinine and liver function tests (transaminases and bilirubin)		X	X	X	X	X	X**
	Potassium			X	X	X	X	X
	Haematocrit, haemoglobin			X (all)			18 month only (in about a 20% subset)	
	Sodium, corrected calcium, phosphate (in about a 20% subset)						18 month only	
	Urinary albumin and creatinine		X					
	Pregnancy test***		X	X	X	X	X	X
Central sample collection	Blood samples for central analysis of creatinine & storage****			X	X	X	X	X
	Urine for central analysis of albumin & creatinine, and storage			X	X		18 month only	X
Randomization & study treatment handling	Issue placebo		X					
	Randomize eligible & willing participants			X				
	Issue randomized treatment (empagliflozin 10 mg or placebo; 210 day supply)			X		X	X	
	Retrieve unused treatment			X		X	X	X
Appointment management & advice	Create appointment		X	X	X	X	X	X
	Provide advice		X	X	X	X	X	X

* Remote follow-up may be used for some participants who are unwilling or unable to attend study visits, and for all surviving participants for several years after the final visit; ** Additional local blood creatinine and urine albumin:creatinine ratio measurement 4 weeks after final follow-up (~20% subset). Final follow-up timing is determined by the Steering Committee in response to numbers of events & DMC recommendations.

*** If pregnancy reasonably possible as indicated by participant's history (or if required by local regulation)

**** NT-pro BNP measured at 0 months and HbA1c measured at 0, 2, 18 months and final visit.

4.3 APPENDIX 3: STUDY ADDRESSES

Sponsor
Boehringer Ingelheim International GmbH
Binger Strasse 173, 55216 Ingelheim, Germany

Central Coordinating Office and Wolfson Laboratories
Clinical Trial Service Unit and Epidemiological Studies Unit, Richard Doll Building,
Old Road Campus, Roosevelt Drive, Oxford OX3 7LF, UK
Tel: +44(0)1865 743868; E-mail: cco.empakidney@ndph.ox.ac.uk

5.1 VERSION HISTORY

EDMS #5434

Version	Version date	Summary
1.0	8th January 2018	Version 1.0 finalized
1.1	8th January 2018	Footnote update
1.2	25th January 2018	Formal interim analysis update
1.3	26th March 2018	Update following MHRA review
1.4	25th April 2018	Update following FDA review of formal interim analysis
2.0	13th January 2020	Recruitment target increase to 6000 participants; exclusion of further participation from people with type 1 diabetes after introduction of a cap; clarification on the subdivisions of cardiovascular death; reducing tertiary subgroup analyses solely to the primary outcome and highlighting the subgroups of key interest; section 3.1.2 exclusion criterion (ix) modified; substituting local creatinine results when central creatinine results are missing; and clarification in table 4.2.1 that Hb/Hct is measured in all participants at randomization (to be consistent with the protocol text in section 3.4.1.1)

A multicentre international randomized parallel group double-blind placebo-controlled clinical trial of EMPAgliflozin once daily to assess cardio-renal outcomes in patients with chronic KIDNEY disease

EMPA-KIDNEY

Data Analysis Plan (EDMS #6290)
(Standard Operating Procedure 11)

Version History

Version	Description	
1.0	First released version	Created by: Natalie Staplin, Will Herrington, Richard Haynes, Jonathan Emberson, Colin Baigent (30MAY2019)<br>Reviewed by Boehringer Ingelheim (sponsor) (JUN2019)<br>Review and agreement by Steering Committee (23OCT2019)<br>Released for use by Will Herrington (01NOV2019)
1.1	Inclusion of Appendix II, subgroup analyses by kidney disease cause, KDIGO risk & analyses using different % declines in eGFR. Clarifications on handling of missing values in subgroup analyses and definitions of tertiles.	Proposed to Steering Committee (30MAR2021)<br>Updated by Will Herrington, Richard Haynes (21JUN2021)<br>Reviewed by Boehringer Ingelheim (sponsor) (JUL2021) and reminder of key changes provided to Steering Committee (26OCT2021)<br>Released for use by Will Herrington (12NOV2021)

This is a controlled document. Distribution and approval is to be managed using the Electronic Document Management System.

Contents

1 Introduction ... 3
2 Baseline characteristics ... 3
3 Definitions for efficacy and safety endpoints ... 4
3.1 Estimands ... 4
3.2 Hypotheses ... 5
3.3 Primary assessments ... 5
3.4 Secondary efficacy assessments ... 5
3.5 Tertiary efficacy assessments ... 6
3.5.1 Subgroup analyses ... 6
3.6 Safety assessments ... 7
3.7 Laboratory assessments ... 8
3.8 Exploratory assessments ... 9
4 Analysis sets for efficacy and safety analyses ... 9
5 Statistical methodology for efficacy and safety analyses ... 10
5.1 Methods of analysis ... 10
5.1.1 Analyses of time to first event ... 10
5.1.2 Recurrent event analyses ... 10
5.1.3 Annual rate of change in eGFR ... 11
5.1.4 Continuous outcomes ... 11
5.1.5 Categorical outcomes ... 12
5.1.6 Exploratory analyses ... 12
5.2 Multiplicity adjustments ... 12
5.3 Subgroup analyses and tests for heterogeneity ... 13
5.4 Handling of missing and incomplete data ... 14
5.4.1 Handling of missing central eGFR values ... 14
5.5 Censoring schema for time-to-event endpoints ... 15
5.5.1 Date of censoring for intention-to-treat analyses ... 15
5.5.2 Date of censoring for on-treatment analyses ... 15
6 Appendix I: Definitions of Study Scheduled Follow-up Visit period windows for analysis purposes 16
7 Appendix II: Algorithm used to substitute local creatinine values when central samples are missing ... 17
8 References ... 18

1 Introduction

The purpose of this Data Analysis Plan is to define, before unblinding of the treatment allocation, all randomized analyses to be presented in the primary publication of the EMPA-KIDNEY trial results. Additional pre-specified analyses and summaries required for regulatory submission will be detailed in a separate analysis plan. The nature of further post-hoc analyses including those related to subsequent publications cannot be specified in detail but, where appropriate, a general analytical approach is set out. The current latest version of SAS® will be used for analyses. Primary and secondary outcomes have been defined in SOP9a (Approach to recording of outcomes, adjudication and primary/secondary outcome definitions; EDMS #5452) and will be adjudicated using SOP9b (Adjudication procedures; EDMS #6062).

2 Baseline characteristics

In order to assess balance of baseline characteristics between randomized arms, the following variables recorded at randomization (or at Screening) will be presented for each of the empagliflozin and placebo groups:

- Age
- Sex
- Region (Europe, North America, China and Malaysia, Japan)
- History of prior disease (presence vs. absence): diabetes mellitus (overall and by type), cardiovascular disease, heart failure, and peripheral arterial disease
- Blood pressure (systolic and diastolic separately)
- Body mass index
- Laboratory values at Randomization:
    - CKD-EPI estimated glomerular filtration rate (eGFR) (mL/min/1.73m^2): as a continuous variable and in the following categories <30, ≥30<45, ≥45 estimated from central enzymatic creatinine (or local creatinine where central value unavailable)
    - Urinary albumin:creatinine ratio (mg/g): as a continuous variable and in the following categories <30, ≥30≤300, >300, or missing (based on central measurement)
    - Glycosylated haemoglobin (HbA1c) (mmol/mol): as a continuous variable and in the following categories <39 (normoglycaemia), ≥39<48 (pre-diabetes), ≥48<75 (well-controlled diabetes), ≥75 (poor glycaemic control), or missing
    - N-terminus prohormone of bone natriuretic peptide (NT-proBNP) as a continuous variable
    - Haematocrit as a continuous variable
- Medication (yes vs. no): renin-angiotensin system (RAS) inhibitors, beta-blockers, diuretics (overall, and loop, thiazide mineralocorticoid receptor antagonists, and other considered separately), LDL lowering medication, antiplatelet therapy, anticoagulants, insulin, sulphonylureas, biguanides, GLP-1 receptor agonists, DPP-4 inhibitors, and other antidiabetic drugs

- KDIGO risk category: low-high versus very high
- Cause of kidney disease: diabetic, glomerular, hypertensive/renovascular, other and unknown

For continuous variables, mean (standard deviation) will be presented unless the variable has a skewed distribution, in which case median (interquartile range) will be used. For all categorical variables, the number and percentage of participants in the category will be presented. All possible categories will be displayed, zero-filled where necessary, the category 'missing' will only be displayed (e.g. in footnotes) if there are actually missing values. Extra details on baseline characteristics may be provided in baseline paper and other publications (see Section 5.4 for details of how missing values are handled in subgroup analyses).

3 Definitions for efficacy and safety endpoints

Unless otherwise specified, all analyses will involve an intention-to-treat comparison among all randomized participants of the effects of allocation to empagliflozin versus placebo during the scheduled treatment period (i.e. all participants will be included irrespective of whether they take none, some or all of their allocated treatment). The event-free survival time will be calculated as the time from the date of randomization to either the date of the first occurrence of the event of interest or the censoring date for those who do not have such an event (see section 5.5 for censoring rules).

In accordance with the protocol, all deaths and events initially reported as hospitalization for heart failure, myocardial infarction, stroke, liver injury, ketoacidosis, lower limb amputation, and acute kidney injury will be subject to adjudication as set out in SOP 9b: Adjudication procedures (EDMS #6062). For those events that are subject to adjudication, analyses will include all confirmed and unrefuted events.

3.1 Estimands

For the efficacy and safety endpoints (with the exception of all cause hospitalizations and annual rate of change in eGFR), the estimand of interest will be the hazard ratio of the first occurrence of the endpoint in the target population for participants allocated empagliflozin relative to those allocated placebo, ignoring any non-fatal intercurrent events and in the hypothetical absence of death from any cause not included in the endpoint.

For all-cause hospitalizations the estimand of interest will be the hazard ratio of all occurrences of the endpoint in the target population for participants allocated empagliflozin relative to those allocated placebo, ignoring any non-hospitalization non-fatal intercurrent events and in the hypothetical absence of death from any cause.

For annual rate of change in eGFR the estimand of interest will be the difference in the mean annual rate of change in eGFR in the target population allocated empagliflozin compared to the target

population allocated placebo, ignoring any non-fatal intercurrent events (except end-stage kidney disease [ESKD], defined as the initiation of maintenance dialysis or receipt of a kidney transplant) and in the hypothetical absence of ESKD or death from any cause.

3.2 Hypotheses

For all statistical tests (other than tests for heterogeneity or trend), the null hypothesis will be that the effect of allocation to empagliflozin on the endpoint of interest in the target population is the same as the effect of allocation to placebo (and hence the alternative hypothesis will be that the effect of allocation to empagliflozin is not the same as the effect of allocation to placebo).

3.3 Primary assessments

The primary endpoint will be the time to the first occurrence of the composite outcome of:

(i) Kidney disease progression (defined as ESKD, a sustained decline in eGFR to <10 mL/min/1.73m^2, renal death, or a sustained decline of ≥40% in eGFR from randomization); or

(ii) Cardiovascular death.

The term 'sustained' will be taken to mean that it is either:

(a) Measured at two consecutive scheduled study Follow-up Visits (at least 30 days apart); or

(b) Measured at the last scheduled study Follow-up Visit or the last scheduled visit before death (or withdrawal of consent or loss to follow-up).

If the conditions for a sustained decline in eGFR are met, the date of the event will be the date of the earlier of the two eGFR measurements.

The analysis method for the primary assessment is described in section 5.1.1.

3.4 Secondary efficacy assessments

The key secondary efficacy outcomes are:

(i) Time to first occurrence of hospitalization for heart failure or cardiovascular death;

(ii) Time to occurrences of all-cause hospitalisations (first and recurrent combined); and

(iii) Time to death from any cause.

The other secondary efficacy outcomes are:

(iv) Time to first occurrence of kidney disease progression;

(v) Time to cardiovascular death; and

(vi) Time to first occurrence of cardiovascular death or ESKD.

The analysis method for the secondary efficacy assessments is described in section 5.1.1, with the exception of the key secondary outcome of recurrent all cause hospitalizations which is given in section 5.1.2.

3.5 Tertiary efficacy assessments

The tertiary efficacy assessments are:

(i) Time to components of kidney disease progression defined as follows:

(a) Time to first occurrence of ESKD, a sustained decline in eGFR to <10 mL/min/1.73m^2, or renal death;

(b) Time to first occurrence of a sustained decline of ≥40% in eGFR from randomization;

(ii) Annual rate of change in eGFR, calculated separately:

(a) For the period from baseline to the final follow-up visit (i.e. “total slope”);

(b) For the period from 2 months to the final follow-up visit (i.e. “chronic slope”);

(iii) Time to first occurrence of ESKD or death from any cause combined;

(iv) Time to first occurrence of kidney disease progression or death from any cause combined;

(v) Time to death from particular categories of causes, including cardiovascular (coronary death, other cardiac [including heart failure and sudden cardiac death not known to be coronary], stroke, other cardiovascular and presumed cardiovascular) and non-cardiovascular (renal, infection, cancer, other medical, and non-medical) causes;

(vi) Time to first occurrence of a major cardiovascular event (defined as the composite of cardiovascular death, myocardial infarction, stroke or hospitalization for heart failure);

(vii) Time to new-onset diabetes mellitus (defined as clinical diagnosis, commencement of glucose-lowering treatment, or HbA1c ≥48 mmol/mol measured by central laboratory on at least one occasion) among participants without diabetes at randomization*, overall and separately among those with normoglycaemia or “pre-diabetes” (defined as HbA1c <39 mmol/mol [normoglycaemia] and ≥39 to <48 mmol/mol [pre-diabetes], respectively);

(viii) Time to first self-reported episode of gout;

(ix) Subgroup analyses of the primary composite outcome (see section 3.5.1 below for details).

* Diabetes at randomization is defined as participant-reported history of diabetes of any type, use of glucose-lowering medication or baseline HbA1c ≥48 mmol/mol at Randomization visit.

The analysis method for the tertiary efficacy assessments is described in section 5.1.1, with the exception of annual rate of change in eGFR which is described in section 5.1.3.

3.5.1 Subgroup analyses

Subgroup analyses are planned for the primary composite outcome. Exploratory subgroup analyses are also pre-specified in section 3.8:

Subgroup subcategories are based on randomization values of:

a. History of prior disease:

   i. Diabetes mellitus* (presence vs absence);

   ii. Cardiovascular disease (presence vs absence);

   iii. Heart failure (presence vs absence);

   iv. Peripheral arterial disease (presence vs absence);

   v. Primary renal diagnosis (diabetic nephropathy, ischaemic/hypertensive, glomerulonephritis; other & unknown combined)

b. Patient characteristics;
   i. Age (split by tertiles of baseline distribution);
   ii. Sex (male vs female);
   iii. Region (Europe, North America, China and Malaysia, Japan);
   iv. Blood pressure (split by tertiles of baseline distribution);
   v. Body mass index (split by tertiles of baseline distribution);

c. Laboratory values at Randomization (as defined in section 2):
   i. HbA1c (split by tertiles of baseline distribution);
   ii. eGFR (<30, ≥30<45, ≥45 mL/min/1.73m^2);
   iii. Urinary albumin:creatinine ratio (<30, ≥30≤300, >300 mg/g);
   iv. NT-proBNP (split by tertiles of baseline distribution);
   v. Haematocrit (split by tertiles of baseline distribution);
   vi. KDIGO risk category (low-high versus very high)

d. Medication use at randomization:
   i. RAS-inhibition (yes vs no);
   ii. Beta-blocker (yes vs no);
   iii. Diuretics (yes vs no).

The analysis method for these subgroup analyses is detailed in section 5.3. The subgroup analyses of the primary composite outcome which are of key interest are those involving subdivision by: (a) baseline diabetes status, (b) baseline eGFR, and (c) urinary albumin:creatinine ratio. The exact cutpoints for age, HbA1c, blood pressure, body mass index, NT-proBNP and haematocrit at randomization will be based on approximate tertiles and specified prior to unblinding.

3.6 Safety assessments

All adverse events are coded using MedDRA version 20.1. The safety outcomes are:

(i) Time to first occurrence of an SAE due to:
   (a) Urinary tract infection, overall and separately by sex;
   (b) Genital infection, overall and separately by sex;
   (c) Hyperkalaemia;
   (d) Acute kidney injury;
   (e) Dehydration;

(ii) Time to first occurrence of an AE of Special Interest (AESI):
   (a) Liver injury, both overall and separately by cause (defined as ALT or AST ≥5x Upper Limit of Normal [ULN] or the combination of ALT or AST ≥3x ULN with bilirubin ≥2x ULN; measured in the same blood sample);
   (b) Ketoacidosis, both overall and separately by baseline diabetes status;

(c) Lower limb amputations (overall and by level [i.e. toe, forefoot, foot, below knee or above knee]);

(iii) Time to first occurrence of another AE relevant to the study question:

(a) Bone fracture, both overall and separately by site (long bones versus non-long bones) and aetiology (i.e. distinguishing those resulting from high and low impact trauma, and other causes);

(b) Severe hypoglycaemia (defined as low blood sugar causing severe cognitive impairment which requires assistance from another person for recovery);

(c) Symptomatic dehydration (defined as whether or not a participant has experienced symptoms they attribute to dehydration, such as feeling faint or fainting);

(iv) Time to first occurrence of hospitalization by specific causes (events are to be categorised according to their MedDRA Primary System Organ Class (SOC));

(v) Time to first occurrence of SAEs both overall and, separately, by Primary SOC category;

(vi) Discontinuation of study treatment overall and by various causes (including SAEs, non-serious adverse events, and other reasons [using Primary SOC categories for SAEs and non-serious adverse events]);

(vii) Changes in weight and systolic and diastolic blood pressure from baseline.

All adverse events and hospitalization endpoints will be analysed as described in section 5.1.1, discontinuation of study treatment will use the methods in section 5.1.5 and changes in weight and blood pressure will be analysed using methods outlined in section 5.1.4.

3.7 Laboratory assessments

Biochemical efficacy assessments using central laboratory measurements will be:

- Difference in mean urinary albumin:creatinine ratio calculated as a weighted average over all post-randomization time points (urine collected at the Randomization visit, 2 months, 18 months and the Final-follow-up visit)
- Difference in mean HbA1c calculated as a weighted average over all post-randomization time points, overall and by those with and without diabetes separately (blood collected at the Randomization visit, 2 months, 18 months and the Final-follow-up visit).

For all participants, biochemical safety assessments using the local laboratory results will be:

- Difference in mean potassium calculated as a weighted average over all post-randomization time points (measured at each scheduled Follow-up Visit)
- Elevations in ALT/AST in various categories (ALT or AST ≥5x ULN, ALT or AST ≥3x ULN with bilirubin ≥2x ULN in the same blood sample measured at each scheduled Follow-up Visit).

In the subset of UK participants, biochemical safety assessments using local laboratory values collected at 18 months of follow-up will also be conducted on:

- Sodium
- Corrected calcium
- Phosphate
- Haematocrit
- Haemoglobin.

See sections 5.1.4 and 5.1.5 for details of how these biochemical markers will be analysed.

3.8 Exploratory assessments

Exploratory assessments are planned to assess the effect of:

a) Allocation to empagliflozin versus placebo on subgroups (using the categories set out in section 3.5.1) for the following outcomes:
   i. Time to kidney disease progression;
   ii. Time to cardiovascular death or hospitalization for heart failure;
   iii. Time to cardiovascular death; and
   iv. Annual rate of change of eGFR.

b) Allocation to empagliflozin versus placebo on:
   i. Mean eGFR at each scheduled visit;
   ii. Subcategories of SAEs by MedDRA High Level Group Terms (HLGTs); and
   iii. The primary outcome by year since Randomization;

c) Effect of stopping study treatment on:
   i. Mean eGFR (using the surviving UK participants with a 4-week post-Final Follow-up blood draw); and
   ii. Urinary albumin:creatinine ratio (using the surviving UK participants with a measurement 4 weeks after Final Follow-up).

d) Allocation of empagliflozin versus placebo on time to the first occurrence of the Kidney disease progression or cardiovascular death, where kidney disease progression utilises the following alternative thresholds:
   i. ≥50% decline in eGFR
   ii. ≥57% decline in eGFR (i.e. approximately consistent with a doubling of creatinine)

The analysis methods to be used for these exploratory assessments are described in section 5.1.6. Additional exploratory assessments may be conducted after unblinding if it is deemed appropriate.

4 Analysis sets for efficacy and safety analyses

For all outcomes, the primary analysis will compare the outcome from randomization to the end of the scheduled treatment period (see section 5.5) among all those participants who are allocated at

randomization to receive empagliflozin versus all those allocated to receive matching placebo (i.e. “intention-to-treat” analyses).[1-3]

For the AESIs (see section 3.6), additional analyses will subsequently be performed to compare the effects of empagliflozin among only those participants who are recorded as being adherent to study treatment at the time of the event (or the preceding follow-up visit) compared to those who are not. However, the emphasis in interpretation will be on the intention to treat analyses. Unless otherwise indicated, all analyses will be based on the first occurrence of the specified outcome.

No further on-treatment analyses for either efficacy or safety will be presented in the primary publication, but may be conducted as part of the regulatory submission process (these will be pre-specified in a separate analysis plan).

5 Statistical methodology for efficacy and safety analyses

5.1 Methods of analysis

5.1.1 Analyses of time to first event

Cox proportional hazards regression adjusted for the variables used in the minimization algorithm (in the same categories used in the minimization process) will be used to estimate the hazard ratio associated with allocation to empagliflozin versus placebo (with the Wald chi-square statistic used to both test significance and generate an asymptotic 95% confidence interval).[4] Any ties will be handled using Breslow’s method. Kaplan-Meier estimates for the time to each of the primary and secondary outcomes will also be calculated. If any regression models fail to converge, the hazard ratio and its confidence interval will instead be estimated from a Cox model adjusted only for treatment allocation. For any outcome where there are insufficient numbers of events to reliably estimate a hazard ratio (e.g. fewer than 5 participants with the event), Fisher’s exact test will be used to compare the number of participants affected in each arm. A participant may contribute to more than one analysis if they have events of more than one type (e.g. hospitalization for heart failure followed by ESKD).

5.1.2 Recurrent event analyses

For the key secondary outcome of all-cause hospitalizations, a semi-parametric joint frailty model will be used[5]. The approach will jointly model:

a) The hazard function for recurrent all-cause hospitalizations conditional on the patient specific random frailty; and
b) The hazard function for time to death conditional on the patient specific random frailty.

It will be assumed that the patient specific random frailty follows a gamma distribution with mean 1 and variance θ, where θ is the correlation between the recurrent events. Piecewise constant hazards will be assumed for both hazard functions to allow estimation of the likelihood by Gaussian quadrature, with follow-up time split into five equally sized intervals.[6] Hazards ratios for the effect of treatment on the

rate of recurrent all-cause hospitalizations and the rate of death will be calculated by the model, but only the former will be formally tested. The joint frailty model will be adjusted for the prognostic variables used in the minimization algorithm (in the same categories used in the minimization process). If the adjusted model fails to converge, then the hazard ratio for recurrent all-cause hospitalizations will instead be estimated from a joint frailty model adjusted only for treatment allocation.

The methods outlined here would also be used if any exploratory analyses for other recurrent event outcomes (e.g. hospitalization for heart failure) are conducted.

5.1.3 Annual rate of change in eGFR

The annual rate of change in eGFR across the whole study (i.e. the “total” eGFR slope) will be compared between all those allocated to empagliflozin and all those allocated to placebo using shared parameter models.[7] The approach will jointly model:

(a) The annual rate of change in eGFR using a linear mixed model with random effects for each patient’s slope and intercept; and

(b) The time to event for ESKD or death using a Weibull survival model in which the scale parameter is assumed to be linearly related to the random effects from the linear mixed model. This allows for the dependence between annual rate of change in eGFR and time to ESKD or death (i.e. those with faster rates of change in eGFR will generally have a shorter time to ESKD or death).

The shared parameter model will be adjusted for the prognostic variables used in the minimization algorithm (in the same categories used in the minimization process). If the adjusted shared parameter model does not converge, then the difference in the annual rate of change in eGFR will instead be estimated from a shared parameter model adjusted only for treatment allocation. The eGFR slope from the 2 month scheduled Follow-up Visit until the last scheduled visits (known as the “chronic” slope) will be assessed using similar methods.

5.1.4 Continuous outcomes

For outcomes with only post-randomization measurements planned (i.e. sodium, corrected calcium, phosphate), mean values of measurements will be compared by t-tests. Where measurements were planned at randomization and a single follow-up visit (e.g. haematocrit and haemoglobin), comparisons of baseline-adjusted mean follow-up values between the allocated treatment arms will be performed using ANCOVA adjusted for each patient’s value at randomization.[8]

Where randomization and multiple post-randomization measurements were planned (i.e. all other continuous outcomes), baseline-adjusted mean follow-up values averaged over time (with weights proportional to the amount of time between visits) will be compared between the allocated treatment arms using a mixed model repeated measures (MMRM) approach. The model will include fixed, categorical effects of treatment allocation, time treatment-by-time interaction, and the prognostic

variables used in the minimization algorithm (in the same categories used in the minimization process) along with continuous effects of baseline (randomization) measurement and baseline-by-time interaction. The within-person error correlations will be assumed to be unstructured. Baseline-adjusted mean values at each of the follow-up times will also be presented (but not formally tested for differences between the allocated treatment arms).

As urinary albumin:creatinine ratio is not normally distributed a log transformation will be applied before analysis.

5.1.5 Categorical outcomes

For categorical outcomes, the effect of allocated treatment on the number of randomized participants with at least 1 event will be compared using chi squared tests, unless any of the expected cell counts in the 2x2 contingency table are less than 5, in which case Fisher's exact test will be used. No adjustment for other covariates will be made.

5.1.6 Exploratory analyses

The effect of allocation to empagliflozin versus placebo on mean eGFR at each scheduled visit will be estimated using the MMRM approach previously outlined in Section 5.1.4, as will the effect of stopping study treatment on mean eGFR and urinary albumin:creatinine ratio (but restricted to the surviving UK participants with a measurement about 4 weeks after Final Follow-up).

Analyses of the effect of allocation to empagliflozin versus placebo on subcategories of SAEs by MedDRA HLGTs will use the Cox proportional hazards model as described in Section 5.1.1.

To assess whether the effect of allocation to empagliflozin versus placebo on the primary outcome differs over time, follow-up time will be split into categories by year since Randomization and the significance of the time x treatment allocation interaction will be assessed.

Technical documentation to accompany this Data Analysis Plan may also be added as an appendix, before any unblinding, if additional methodological details for the approaches described in section 5 are found to be required.

5.2 Multiplicity adjustments

As a formal interim efficacy analysis performed by the DMC is planned during the trial (see DMC charter [EDMS #5708] for details), the required alpha-level for the primary outcome in the final analysis will be adjusted as per the Hwang-Shih-DeCani alpha-spending function (γ=-8). For example, with 60% of the primary outcomes accrued at the time the interim analysis, the final two-sided p-value would need to be <0.0497 to be deemed statistically significant.

If the primary assessment shows a significant benefit of empagliflozin, the key secondary outcomes will be assessed with their p-values corrected for multiple testing using the Hochberg “step-up” procedure that controls the familywise error rate. To account for the formal interim efficacy analysis, the Hwang-Shih-DeCani alpha-spending function (γ=0) will be used to specify the familywise error rate. Other secondary outcomes will be assessed without adjustment for multiplicity at a nominal level of $\alpha = 0.05$ (two-sided). Further details are provided in Section 2.5.2.2 of the protocol.

If based on the results of the interim analysis (which will be based in large part on local creatinine results), stopping criteria are met and the DMC recommend stopping the trial for efficacy, substantial further data will be collected as participants will attend their final follow-up visits and provide latest information on their renal status and blood for central creatinine analysis (to apply the definition of sustained). It may take a several weeks/months to see all participants at a final follow-up visit at each site, as the trial aims to establish large sites. All analyses will be based on final database using the results from the final follow-up visits. Note the information fraction used in the alpha-spending function for the primary and key secondary outcomes will be based on the number of primary outcome events observed at the time of the DMC analysis.

If a formal interim efficacy analysis is not conducted, for example for operational reasons, the primary outcome will be assessed without adjustment for multiplicity and the key secondary outcomes controlled at the familywise error rate of 5.0%.

For the tertiary efficacy, subgroup, safety, laboratory and exploratory analyses, allowance in their interpretation will be made for multiple hypothesis testing,[1, 2] taking into account the nature of events (including timing, duration and severity) and evidence from other studies. In addition to the pre-specified comparisons, other post-hoc analyses may be performed with due allowance for their exploratory and, perhaps, data-dependent nature.

5.3 Subgroup analyses and tests for heterogeneity

Tests for heterogeneity of the proportional effect observed in subgroups, through the inclusion of relevant interaction terms (with main effects if not already included in model) in Cox models for time to event outcomes and the shared parameter model for annual rate of change in eGFR, will be used to determine whether the proportional effects in specific subcategories are clearly different from the overall effect. If, however, three or more patient categories can be arranged in some meaningful order then assessment of any trend will be made. Tests of trend will be conducted for age, blood pressure, body mass index, HbA1c, eGFR, urinary albumin:creatinine ratio, NT-proBNP and haematocrit by assessing the significance of the interaction term between treatment allocation and the relevant linear continuous factor.

5.4 Handling of missing and incomplete data

Missing eGFR values will be handled as detailed below. Participants with missing values relevant to subgroup analyses (e.g. body mass index) will be included in the subgroup containing the median value.

5.4.1 Handling of missing central eGFR values

eGFR measured at scheduled visits will be calculated using the CKD-EPI formula,[9] irrespective of whether the creatinine is measured centrally or locally. Appendix I provides definitions of when scheduled Follow-up Visits are expected and how follow-up periods are defined for the purposes of analyses. If multiple central eGFR measurements are available in any one scheduled follow-up period, then the eGFR closest to the ideal follow-up day will be used to define the eGFR for that particularly scheduled Follow-Up Visit period. The eGFR measured at the Final Follow-up Visit will be usually be the last of the eGFRs to be included in analyses, and will be included irrespective of whether or not it is the eGFR closest to the ideal follow-up day. This may result in two eGFRs in the final scheduled follow-up period.

eGFR will be estimated from creatinine measured in the central laboratory wherever possible, but where a central laboratory eGFR measurement is expected (e.g. because a scheduled Follow-up Visit was completed whilst the participant was alive) but missing, the local blood creatinine measurement closest to the ideal follow-up day within the scheduled Follow-up visit period (if one exists) will be used to estimate the local eGFR in its place. In instances where a local Follow-up Visit eGFR is used, percentage change in eGFR will be calculated relative to the local Randomization Visit eGFR measurement and the local eGFR closest to the ideal follow-up day of the next scheduled Follow-up Visit period with a local measurement used to assess the definition of sustained (see Appendix II for details of how this is to be implemented).

Sensitivity analyses where the ≥40% decline and <10 ml/min/1.73m^2 eGFR components of the Kidney Disease Progression outcome are based solely on central laboratory eGFR measurements will be conducted.

Those without any Randomization Visit eGFR measurement (central or local) will still be included in the ≥40% decline in eGFR component of the Kidney Disease Progression outcome by using the latest pre-randomization locally measured eGFR value. Sensitivity analyses excluding these participants from the ≥40% decline in eGFR component of the Kidney Disease Progression outcome (but still counting them in the other components of the primary outcome) will be conducted.

eGFR slope analyses will use all eGFR measurements. The main analysis for eGFR slopes is restricted to only central eGFR measurements. Sensitivity analyses using only local eGFR measurements (both with and without the local eGFR measurements planned for 4 weeks after the end of scheduled study treatment in surviving UK participants) will be performed.

5.5 Censoring schema for time-to-event endpoints

5.5.1 Date of censoring for intention-to-treat analyses

It is the aim at each scheduled Follow-up Visit to ascertain all components of the primary outcomes, including the Final Follow-up Visit, with subsequent adjudication of any recorded deaths. Follow-up Visits are usually conducted by direct participant interview and central blood sampling. Occasionally follow-up will need to be by interview with their relative, carer or doctor, or by medical record review (e.g. paper, electronic or registry). During such indirect follow-ups, the date last known to be alive is recorded. Every effort to collect a central blood sample is made at each scheduled visit (direct or not), but for those known to be alive who do not provide a blood sample, the most recent local blood creatinine measurement can be recorded.

Censoring dates for those who withdraw consent or who are lost to follow-up will be derived from information collected at their most recent Follow-up Visit before consent withdrawal or loss to follow-up. Otherwise, the censoring date will be the date of death or the date of the Final Follow-up Visit (except when the Final Follow-up Visit is indirect, in which case the date last known to be alive will be used). Outcomes recorded as starting after the Final Follow-up Visit will not be included in any within-trial analyses.

5.5.2 Date of censoring for on-treatment analyses

A censoring date for on-treatment analyses may need to be derived for safety assessments (see Section 3.6). For those participants compliant with any dose of study treatment at their Final Follow-up Visit (or their last Follow-up Visit before death, withdrawal of consent or loss to follow-up), their on-treatment analysis censoring date will be their intention-to-treat analysis censoring date. Where participants are recorded as stopping study treatment and are never recorded as restarting, their on-treatment analysis censoring date will be the date they were recorded as stopping treatment, plus an additional 7 days to allow for any residual effect of treatment.

6 Appendix I: Definitions of Study Scheduled Follow-up Visit period windows for analysis purposes

Scheduled Follow-up Visits relative to the Randomization Visit date			
**Visit number**	**Follow-up month**	**Follow-up period**	**Ideal follow-up day**
1	2	≥1, <121 days	60 days
2	6	≥121, < 271 days	180 days
3	12	≥271, <451 days	360 days
4	18	≥451, <631 days	540 days
5	24	≥631, <811 days	720 days
6	30	≥811, <991 days	900 days
7	36	≥991, <1171 days	1080 days
8	42	≥1171, <1351 days	1260 days
9	48	≥1351, <1531 days	1440 days
10…	54...	≥1531, <1711 days…	1620 days

7 Appendix II: Algorithm used to substitute local creatinine values when central samples are missing

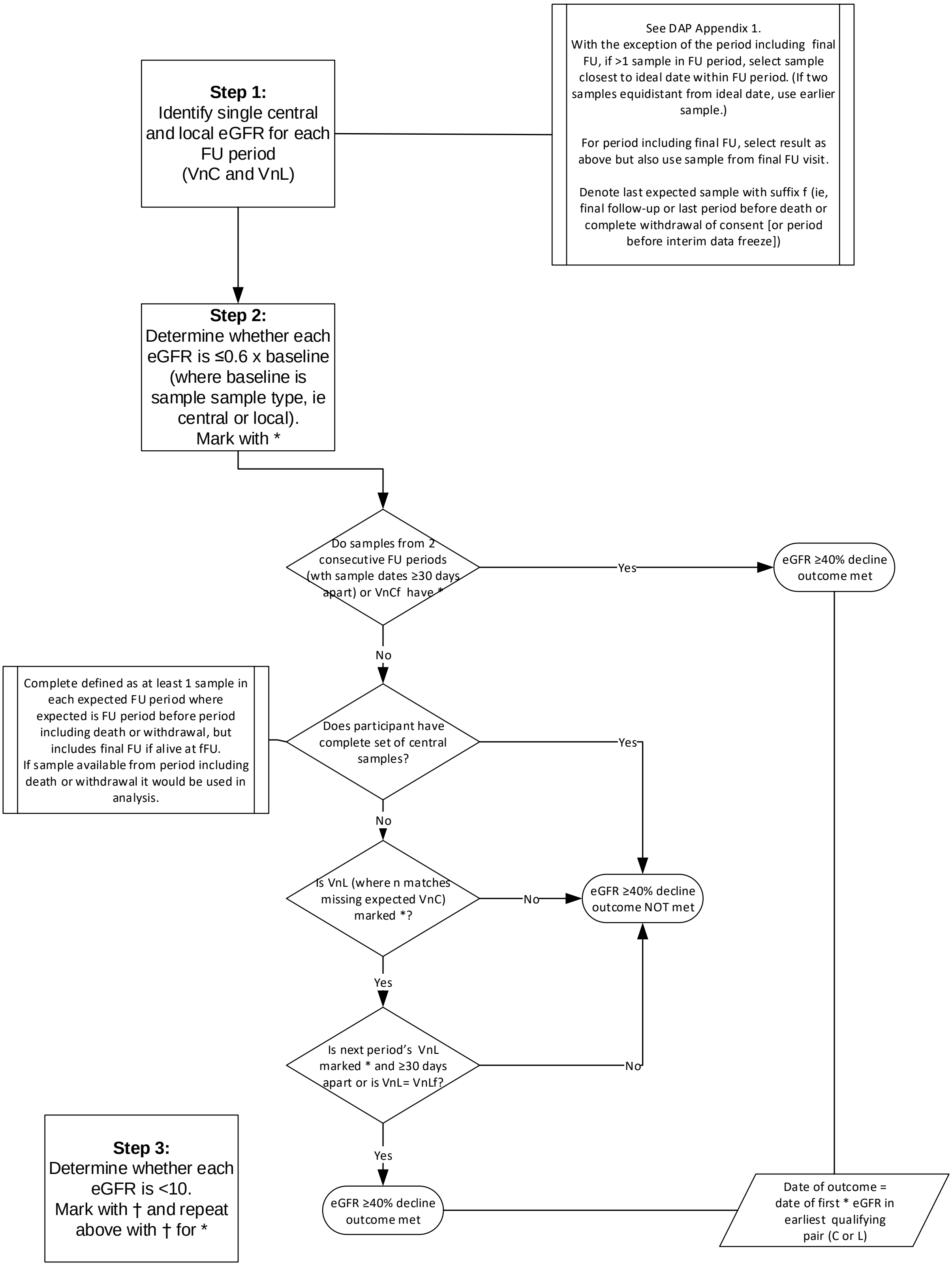

8 References

1. Peto R, Pike MC, Armitage P, Breslow NE, Cox DR, Howard SV, et al. Design and analysis of randomized clinical trials requiring prolonged observation of each patient. I. Introduction and design. British Journal of Cancer. 1976; **34**(6): 585-612.
2. Peto R, Pike MC, Armitage P, Breslow NE, Cox DR, Howard SV, et al. Design and analysis of randomized clinical trials requiring prolonged observation of each patient. II. analysis and examples. British Journal of Cancer. 1977; **35**(1): 1-39.
3. Peto R, Peto J. Asymptotically efficient invariant test procedures. J Roy Stat Soc. 1972; **135**(2): 185-207.
4. Cox DR. Regression Models and Life-Tables. J R Stat Soc B. 1972; **34**(2): 187-+.
5. Rogers JK, Yaroshinsky A, Pocock SJ, Stokar D, Pogoda J. Analysis of recurrent events with an associated informative dropout time: Application of the joint frailty model. Statistics in Medicine. 2016; **35**(13): 2195-205.
6. Liu L, Huang X. The use of Gaussian quadrature for estimation in frailty proportional hazards models. Statistics in Medicine. 2008; **27**(14): 2665-83.
7. Vonesh EF, Greene T, Schluchter MD. Shared parameter models for the joint analysis of longitudinal data and event times. Statistics in Medicine. 2006; **25**(1): 143-63.
8. Vickers AJ, Altman DG. Analysing controlled trials with baseline and follow up measurements. BMJ. 2001; **323**(7321): 1123-4.
9. Levey AS, Stevens LA, Schmid CH, Zhang YL, Castro AF, Feldman HI, et al. A new equation to estimate glomerular filtration rate. Ann Intern Med. 2009; **150**(9): 604-12.

Members of the EMPA-KIDNEY Collaborative Group

Executive Committee
Colin Baigent (co-Chair), Martin J. Landray (co-Chair), Christoph Wanner (Deputy Chair), William G. Herrington (Chief Investigator), Richard Haynes (co-Principal Investigator), Jennifer B. Green, Sibylle J. Hauske*, Martina Brueckmann*, Mark Hopley*
(Previous members: Maximillian von-Eynatten* & Jyothis George*)

Steering Committee
Executive Committee members plus National Representatives: Susanne Brenner (Germany); Alfred K. Cheung (United States); David Preiss (United Kingdom); Zhihong Liu, Jing Li (China); Laiseong Hooi, Wen Liu (Malaysia); Takashi Kadowaki, Masaomi Nangaku (Japan); Adeera Levin, David Cherney (Canada); Roberto Pontremoli, Aldo Pietro Maggioni (Italy); plus statistician members: Natalie Staplin, Jonathan Emberson, Stefan Hantel*; plus other expert members: Shinya Goto, Rajat Deo, Katherine Tuttle. Non-voting members: Sarah Y.A. Ng, Francisco Javier Rossello Lozano, Emily Sammons, Doreen Zhu
* denotes a Boehringer Ingelheim employee

Independent Data Monitoring Committee
Peter Sandercock (Chair), Rudolf Bilous, Charles Herzog, Paul Whelton, Janet Wittes, Derrick Bennett (non-voting statistician)

Central Coordinating Office

Administration: Patricia Achiri, Chrissie Ambrose, Jill Barton, Richard Brown, Andy Burke, Sebastian Butler, Rejive Dayanandan, Pia Donaldson, Robert Dykas, Lucy Fletcher, Kate Frederick, Hannah Kingston, Mo Gray, Emily Harding, Akiko Hashimoto, Lyn Howie, Susan Hurley, Ryonfa Lee, Nik Luker, Kevin Murphy, Mariko Nakahara, John Nolan, Michelle Nunn, Sorcha Mulligan, Akiko Omata, Sandra Pickworth, YanRu Qiao, Shraddha Shah, Karen Taylor, Alison Timadjer, Monique Willett, Liz Wincott, Qin Yan, Hui Yu; *Clinical*: Louise Bowman, Fang Chen, Robert Clarke, Michelle Goonasekera, Richard Haynes, William G. Herrington, Parminder Judge, Waseem Karsan, Marion Mafham, Kaitlin J. Mayne, Sarah Y. A. Ng, David Preiss, Christina Reith, Emily Sammons, Mohammed Zayed, Doreen Zhu; *Data Analysis*: Ritva Ellison, Rowan Moys, Will Stevens, Kevin Verdel, Karl Wallendszus; *Finance*: Chris Bowler, Anna Brewer, Andy Measor; *IT Validation*: Guanguo Cui, Charles Daniels, Angela Field, Bob Goodenough, Ashley Lawson, Youcef Mostefai, Dheeptha Radhakrishnan, Samee Syed, Shuang Xia; *Laboratory*: Ruth Adewuyi-Dalton, Thomas Arnold, Anne-Marie Beneat, Anoushka Bhatt, Chloe Bird, Andrew Breach, Laura Brown, Mark Caple, Tatyana Chavagnon, Karen Chung, Sarah Clark, Luminita Condurache, Katarzyna Eichstadt, Marta Espino Obrero, Scarlett Forest, Helen French, Nick Goodwin, Joanne Gordon, Cat Guest, Tina Harding, Michael Hill, Michal Hozak, Matthew Lacey, David MacLean, Louise Messinger, Stewart Moffat, Martin Radley, Claire Shenton, Sarah Tipper, Jon Tyler, Lesley Weaving, James Wheeler, Elissa Williams, Tim Williams, Hamish Woodhouse; *Monitoring*: Angela Chamberlain, Jo Chambers, Joanne Davies, Denise Donaldson, Pati Faria-Shayler, Denise Fleming-Brown, Jennifer Ingell, Carol Knott, Anna Liew, Helen Lochhead, Juliette Meek, Isabel Rodriguez-Bachiller, Andrea Wilson, Patrick Zettergren; *Programming*: Rach AitSadi, Ian Barton, Alex Baxter, Yonghong Bu, Lukasz Danel, Sonja Grotjahn, Rijo Kurien, Michael Lay, Archie Maskill, Aleksandra Murawska, Rachel Raff, Allen Young; *Principal Investigators*: Colin Baigent, Richard Haynes, William G. Herrington, Martin J. Landray, David Preiss; *Statistics*: Jonathan Emberson, Rebecca Sardell, Natalie Staplin

Regional Coordinating Centres

Germany: *National Representative*: Christoph Wanner; Vladimir Cejka, Marcela Fajardo-Moser, Christian Hartner, Anja Knoppe, Doris Poehler, Janina Renner, Franziska Scheidemantel

United States: *National Representative*: Jennifer B. Green; Miya Bryant, Anita Hepditch, Cassandra Johnson, Erin Latore, Lauren Price, Merilee Whalen, Ashleigh Wheeler

UK: *National Representatives*: Richard Haynes, David Preiss; Jo Chambers, Joanne Davies, Denise Donaldson, Mo Gray, Emily Harding, Jenny Ingell, Yanru Qiao, Shraddha Shah, Andrea Wilson, Patrick Zettergren

China: *National Representatives*: Zhihong Liu, Jing Li; Yu An, Yinghua Chen, Peiling Chen, Hao Dai, Hong Du, Fang Feng, Qing Guo, Libo Hou, Wuhanbilige Hundei, Binbin Jin, Yan Li, Jiamin Liu, Xia Song, Yanping Wang, Yanwu Yu, Ning Zhang, Lingshan Zhao, Hui Zhong

Malaysia: Cheng Beng Goh, Ye Mun Low, Soon Yi Sor, Farah Hanis Zulkipli, Sarojini Sivanandam

Japan: Natsuki Arai, Ai Fukasawa, Mizue Furukawa, Keisuke Habuki, Shoko Hayashi, Wakako Isari, Saki Kanegae, Maria Kawai, Reiki Kobayashi, Takako Kuramae, Chika Kuribayashi, Sawako Maeno, Satoshi Masumoto, Tomoko Morisaki, Minoru Oda, Kazue Sawada, Kenta Sugamori, Ayana Tatsuzawa, Aiko Tomita, Kazuyuki Yuasa, Hiroko Inazawa

Canada: *National Representative*: Adeera Levin*;* Amanda Axler, Kerri Gallo

Italy: *National Representative*: Aldo Pietro Maggioni; Ester Baldini, Barbara Bartolomei Mecatti, Francesca Bianchini, Martina Ceseri, Laura Cipressa, Gianna Fabbri, Andrea Lorimer, Donata Lucci

Local Clinical Centres

Germany: *Universitatsklinikum Wurzburg*: Christoph Wanner, Susanne Brenner, Vladimir Cejka, Sharang Ghavampour, Anja Knoppe; *Zentrum fuer Nieren-, Hochdruck- und Stoffwechselerkrankungen Hannover*: Hans Schmidt-Gurtler, Sybille Merscher, Georg Rainer Schlieper, Anke Torp, Maja Zietz; *Nephrologisches Zentrum Villingen-Schwenningen*: Bernd Hohenstein, Urs Benck, Diliana Draganova, Thomas Weinreich, Lothar Wolf, Jasmine Gaidu; *Nierenzentrum Freiburg*: Daniel Steffl, Marie Breitenfeldt, Annette Kraemer-Guth, Christine Braun, Simone Hagge*; Dialysezentrum Heilbronn*: Michael Schomig, Stephan Matthias, Beate Schumacher; *Klinikum der Universitat Munchen*: Thomas Sitter, Louise Fuessl, Julia Krappe, Victoria Kretschmer, Jerome Loutan, Marija Petkovic, Volker Vielhauer, Marc Weidenbusch, Magdalena Maurer; *ClinPhenomics GmbH Co. KG*: Bernhard Winkelmann, Martin Dursch, Linda Tenbusch; *Universitatsmedizin Mainz*: Julia Weinmann-Menke, Simone Boedecker, Wiebke Kaluza-Schilling, Daniel Kraus, Carina Krieger, Margit Schmude, Anne Schreiber; *Herz- und Diabeteszentrum Nordrhein-Westfalen*: Diethelm Tschope, Abdulwahab Arbi, Maryam Kalusche-Afsah, Young Lee-Barkey, Bernd Stratmann, Natalie Prib, Sina Rolfsmeier, Irina Schneider; *Universitatsklinikum Dusseldorf*: Lars Rump, Johannes Stegbauer, Claudia Schmidt; *Nephrocare Mettmann - Standort Velbert*: Michael Koch, Sendogan Aker, Annika Hoeschen; *Diaverum MVZ Potsdam*: Thiemo Pfab, Christian Albert, Michael Haase, Barbara Zander, Claudia Schneider-Danwitz; *Praxis fur Dialyse und Nierenkrankheiten - Arztezentrum Helle Mitte*: Wolfgang Seeger, Wolf-Adam Seeger, Britta Zemann; *Klinikum Bielefeld*: Christoph Stellbrink, Kristin Marx, Ekaterina Stellbrink, Britta

Brettschneider; *Studienzentrum Aschaffenburg*: Gerhard Klausmann, Inga-Nadine Kummer, Auguste Kutschat, Simone Streitenberger; *Universitatsklinikum Halle*: Matthias Girndt, Silke Markau, Ina Girakossyan, Claudia Hanf; *Klinikum St. Georg Leipzig*: Joachim Beige, Ralph Wendt, Ulrike Schmidt; *Studienzentrum Nephrologie Nurnberg-Langwasser*: Andreas Schneider, Roland Veelken, Claudia Donhauser; *UBAG fur Nephrologie und Dialyse Neckarsulm*: Luis Becker, Nexhat Miftari, Ricarda Wolfling; *Universitatsklinikum Dresden*: Christian Hugo, Alexander Paliege, Jens Passauer, Julian Stumpf, Annegret Fleischer, Kerstin Haaser; *Universitatsklinikum Mannheim*: Bernhard Kraemer, Jan Jochims, Bernd Kruger, Thomas Michaeli, Claudia Foellinger; *Nierenzentrum Wiesbaden*: Frank Strutz, Stefan Haack, Ursula Hohenstatt; *Universitatsklinikum Jena*: Martin Busch, Konstantin Herfurth, Gunter Wolf, Rainer Paul; *Studienzentrum fur Nieren- und Hochdruckerkrankungen Hannover*: Hermann Haller, Jessica Kaufeld, Jan Menne, Elisabeth Bahlmann-Kroll, Angela Bergner; *Universitatsklinikum Augsburg*: Horst Weihprecht, Aydin Er, Elif Turan; *Nephrologisches Zentrum Gottingen*: Volker Schettler, Egbert Schulz, Elke Schettler; *Universitaetsklinik Ulm*: Bernd Schroppel, Rene van Erp, Martin Kachele, Ulla Ludwig, Lena Schulte-Kemna, Waltraud Kmietschak, Martina Ruocco; *AGAPLESION Markus-Krankenhaus*: Gunnar Heine, Martin Brzoska, Sebastian Gabel, Christina Hantzi; *Universitatsklinikum Regensburg*: Bernhard Banas, Bernadette Assenbrunner, Tobias Bergler, Yvonne Ehrl, Franz Putz, Antonia Schuster, Stefanie Kuhn, Torsten Schramm; *DaVita Viersen - Nettetal*: Stefan Degenhardt, Gerhard Schmidt, Lea Weiland, Ulrike Giebeln-Hudnell; *Klinikum Braunschweig*: Jan Kielstein, Gabriele Eden, Gina Morig, Manuela Winkler; *Nephrocare Mettmann*: Annika Hoeschen; *Vivantes Klinikum Neukolln*: Harald Darius, Charalampos Kriatselis, Carl-Philipp Roesch; *Robert-Bosch-Krankenhaus Stuttgart*: Dominik Alscher, Markus Ketteler, Jens Kruth, Moritz Schanz, Severin Schricker, Bianka Rettenmaier

United States: *Clinical Advancement Center*: Pablo Pergola, Irene Leal, Melissa Cagle, Anna Romo, Anthony Torres; *Seacoast Kidney and Hypertension Specialists*: Sucharit Joshi, Kulli Barrett, Alexis Africano, Vicki Dodds, Dorleena Gowen, Ashlee Morris; *Total Research Group, LLC*: Juan Fernandez, Guillermo Jimenez, Ricardo Viera, Ryan Barrios, Maylin Garcia, Kerelyn Garcia, Iradis Leal; *Nephrology Consultants, LLC*: David Tietjen, David Bains, Carlo Castillo, Genielle Brewer, Justin Davis, Natalie Freking, Brittany Golson, Sally Ham, Jesslyn Roesch; *Sumter Medical Specialists*: Pusadee Suchinda, Shameem Beigh, Usah Lilavivat, Joyce Bilton, Kim Bocchicchia; *Yale University*: Jeffrey Turner, Neera Dahl, Aldo Peixoto, Yasemin Kavak, Hari Nair, Nicolas Page, Stephanie Rosenberg, Kathryn Simmons; *Northwestern University*: Tamara Isakova, Rebecca Frazier, Rupal Mehta, Anand Srivastava, Patrick Fox, Jonathan Hecktman, Alexander Hodakowski, Carlos Martinez, Rachel Phillips, Alexis Stevenson; *University of Kansas Medical Center*: Reem Mustafa, Kyle Jansson, Cassandra Kimber, Jason Stubbs, Ahmad Tuffaha, Sri Yarlagadda, Debbie Griffin, Zhuo Tang; *Providence Sacred Heart Medical Center and Childrens Hospital*: Radica Alicic, Katherine Tuttle, Ann Cooper, Lisa Davis; *East Coast Research Institute*: Ashwini Gore, Rebecca Goldfaden, Leslie Harvill, Lisa Hichkad, Barry Johns, Thomas Jones, Kayla Merritt, Jennifer Sheldon, Jennifer Stanfield, Lindsay Alexander, Kaitlyn Preston, Lindsey Wood; *Monument Health*: Rajesh Pradhan, Roger DeRaad, Kelli McIntosh, Louis Raymond, Michael Shepperd, Susan McLaughlin, Mary Seifert, Andrew Shepherd; *Mountain Kidney & Hypertension Associates*: Joseph Aiello, William Durham, Laurie Loudermilk, John Manley, Sabrina Burnette, Stephanie Evans, Tara Johnson; *Texas Institute for Kidney and Endocrine Disorders*: Lance Sloan, Judy Ann Acosta, Stacy Gillham, Katia Sloan, SueAnn Squyres; *Wake Forest University Health Sciences*: Michael Rocco, Amret Hawfield, Ben Bagwell, Lauren Richmond; *Chase Medical Research*: Joseph Soufer, Subha Clarke, Amanda Aliu, Kristine Calabrese, Amanda Davis, Veronica Poma, Tracy Spinola; *East Coast Institute for Research LLC*: James Magee, Ricardo Silva, Rushab Choksi, Lorraine Dajani, John Evans, Anil George, Rebecca Goldfaden, Prasanth Krish, Gerard Martins, Mae Sheikh-Ali, David Sutton, Freda Driver, Abraham Hanburry, Laura Hume, Amber Hurst, Matthew Taddeo, Marla Turner, Veronica Yousif; *University of Utah Health Sciences*: Srinivasan Beddhu, Laith Al-Rabadi, Nikita Abraham, Amalia Caamano, Judy Carle, Victoria Gonce, Kaitlyn Staylor, Na

Zhou; *University of Texas Health Science Center at San Antonio*: Shweta Bansal, Manoj Bhattarai, Kumar Sharma, Subrata Debnath, Aliseiya Garza, Chakradhar Velagapudi; *Academy of Diabetes, Thyroid, and Endocrine, PA*: Sergio Rovner, Javier Almeida, Pablo Casares, Verlaine Stewart-Ray, Rene Almaraz, Renata Dayrell, Ana Moncada, Ricardo Pulido, Roxana Rodriquez; *East Coast Institute for Research*: James Magee, Wasim Deeb, Kathryn DeGoursey, Rodel Gloria, Trevor Greene, Robert Miller, Edward Pereira, Miguel Roura, Mae Sheikh-Ali, David Sutton, Debbie Domingo, Sasha Dorestin, William Hodge, Cathy Jackson, Deborah Lund, Katrina Taylor; *Aventiv Research*: Kenneth Boren, Brittany Cleveland, Sandra Gaiser, Mandeep Sahani, Logan Aldrich, Exodus Edmerson, Edmond Limon, Cole Valletta, Patricia Vasquez; *St. Clair Nephrology Research*: Christopher Provenzano, Navkiranjot Brar, Heather Henderson, Bellovich Keith, Qur Khai, Quresh Khairullah, Gail Makos, Joel Topf, Sherry Gasko, Rosemarie Henschel, Kaitlin Knapp, Teresa Kozlowski, Paula LaFleur, Ashwathy Varughese; *Kaiser Permanente San Diego*: Hui Xue, Patricia Wu, Olga Arechiga, Shan Darbeau, Michael Fechter, Stephanie Martinez; *Hanson Clinical Research Center*: Lenita Hanson, Nyla Cooper, Arelis Madera, Jay Cadorna, Rita Sheridan, Helen Sparks; *Saint Elizabeth Healthcare*: Bradley Eilerman, Susanne Bodine, Wael Eid, Rebecca Flora, Amber Avery, Cashmere Hardy; *Thomas Jefferson/ARIA Health Northeast Endocrine Metabolic Associates*: Mihaela Biscoveanu, Steven Nagelberg, Tracey Cummins; *Emory University*: Frederic Rahbari-Oskoui, Anju Oommen, Zohreh Forghani, Stacie Hitchcock, Darya Hosein, Diane Watkins; *East Coast Institute Research, LLC*: Minesh Patel, Anthony Lambert, Elizabeth Newman, Autumn Wood, Tammy Ross, Stephany Topping; *Kidney Care and Transplant Services of New England*: Jeffrey Mulhern, Lorna Murphy, Ann Vasseur; *Brookview Hills Research Associates LLC*: Gregory Greenwood, Alexander Hadley, Denise Laurienti, Christopher Marshall, Nicholas McLean, Scott Satko, Brandy Caudill, Jacob Maris, Janice Rogers, Cindy Vanhoy; *Cleveland Clinic*: George Thomas, Georges Nakhoul, John O'Toole, Jonathan Taliercio, Leslie Cooperman, Marina Markovic, Barbara Tucky; *Salem V.A. Medical Center*: Devasmita Dev, Alia Hasan, Hima Yalamanchili, Lesley McNeil, Eric Wines; *Medstar Health Research Institute*: Jean Park, Adline Ghazi, Mia Hamm, Tejas Patel; *University of North Carolina Hospital*: Amy Mottl, Emily Chang, Emmie Cole, Anne Froment, Sara Kelley, Jordan Osmond Foster; *Olive View - UCLA Medical Center*: Vahid Mahabadi, Golriz Jafari, Anita Kamarzarian, Wendy Arriaga, Daisy Arteaga, Genesis Naverrete; *P&I Clinical Research, LLC*: Prashant Kumar, Imran Nazeer, Karina Urquia, Tammi Glider, Vickie Jones, Savannah Rucker, Jennifer Wiley; *Pioneer Research Solutions*: Rahul Pandey, Jesus Arroyo, Harish Pariani, Mohammad Ahmad, Shahin Mozaffari, Erika Perez; *Los Angeles Biomedical Research Institute at Harbor-UCLA Medical Center*: Matthew Budoff, Sion Roy, Divya Birudaraju, Ahmed Ghanem, Sajad Hamal; *Research Institute of Dallas*: Stephen Aronoff, Elisa Joye Petr, Richard Sachson, Jaime Wiebel, Sana Akram, Laurie Jones, Maurie Tarlac; *Renal Disease Research Institute*: Shahbaz Ahmed, Harold Szerlip, Akinwande Akinfolarin, Ankit Mehta, Shana Camp, Cindy Castro, Zanaida Cooper, Jessica Terry; *Clinical Research Consultants*: Ahmed Awad, Bhavya Kothapalli, Ryan Lustig, Serine Alfaress, Hyder Jasim, Mary Parrigon; *Lexington V.A. Health Care System*: Dennis Karounos, Sadiq Ahmed, Maggie Berry, Ruth Oremus; *VA Southern Nevada Healthcare System*: Carlos Hernandez-Cassis, Elias Ugwu, Nazia Junejo, Nancy Suazo; *University of Florida Health*: Mark Segal, Amir Kazory, Sherry Brown, Tristan Daniels, Sofia Dayi, Renee Hogan, Kathy McCray, Jennifer Stickley; *University Hospitals Cleveland Medical Center*: Mahboob Rahman, Mirela Dobre, Lavinia Negrea, Aparna Padiyar, Nishigandha Pradhan, Arash Rashidi, Nagaraju Sarabu, Vicki Donley, Tricia Young; *Midland Florida Clinical Research Center*: Godson Oguchi, Judepatricks Onyema, Kahla Damianik, Jack Dienes, Judith Plummer-Morgan, Marilyn Roman, Mauver Skipper, Stacey-Ann Villaruel; *Cedar Crosse Research Center*: Danny Sugimoto, Jeffrey Dugas, Ismeal Ahmed, Jamie Bhairoo, Dolores Rijos, Huzaifa Salim

UK: *Oxford University Hospitals*: Richard Haynes, William G. Herrington, Doreen Zhu, Madita Gavrila, Kathryn Lafferty, Ria Rabara, Sally Ruse, Maria Weetman; *Southmead Hospital, Bristol*: James Bushnell, Albert Power, Alison Jenkins, Stefanie Jones, Amanda Scott; *Nottingham City Hospital*: Cath Byrne, Mark Jesky, Alison Cowley, Emma McHaffie, Holly

Waterfall; *Dorset County Hospital*: Jo Taylor, Laura Bough, Thomas Phillips, Barbara Winter-Goodwin; *King's College Hospital, London*: Sui Phin Kon, Iain MacDougall, Eirini Lioudaki, Sapna Shah, Claire Sharpe, Francisco Aguilar, Abegail Hernandez Pena, Conception Pugay, Amelia Te; *Queen Elizabeth Hospital Birmingham*: Wasim Hanif, Samiul Mostafa, Alice Aitken, Katharine Draxlbauer, Evelina Grobovaite, Jennifer Kearney, Theresa McCarthy; *Royal Cornwall Hospital*: Giorgio Gentile, Duncan Browne, Palanichamy Chellamuthu, Tabinda Dugal, Terri Chant, Laura Jones, Emily Laity, Megan Miners, James Muir, Elizabeth Swanson; *Imperial College Healthcare NHS Trust*: Andrew Frankel, James Tomlinson, Marlon Alegata, Anthoula Apostolidi, Maria Vourvou, Thomas Walters; *Royal Derby Hospital*: Maarten Taal, Hari Dukka, Nitin Kolhe, Carly McDonald, Kelly White; *The Queen Elizabeth Hospital, King's Lynn*: Shiva Ugni, Smita Gunda, Rotimi Oluyombo, Vicki Brindle, Ping Coutts, Tracy Fuller, Evelyn Nadar; *Princess Royal Hospital, Telford*: Suresh Ramadoss, Denise Donaldson, Nichola Motherwell, Susannah Pajak, Louise Tonks; *Hull Royal Infirmary*: Sunil Bhandari, Richard Bodington, Adil Hazara, Dominic Fellowes; *University Hospital Aintree*: Christopher Wong, Christopher Goldsmith, Sherald Barnes, Ann Bennett, Claire Burston, Samantha Hope, Nicola Hunt; *UHNM Royal Stoke University Hospital*: Richard Fish, Daniela Farrugia, Judy Lee, Emma Sadler, Hannah Turner; *Belfast City Hospital*: Christopher Hill, Henry Brown, Agnes Masengu, Peter Maxwell, Nina Bleakley, Hugh Murtagh; *West Suffolk NHS Foundation Trust*: William Petchey, Vivian Yiu, Joanne Kellett, Angharad Williams; *Royal Devon and Exeter Hospital*: Helen Clarke, Victoria Carnall, Sarah Benyon, Caroline Blake, Stephanie Estcourt, Jane Piper; *Daisy Hill Hospital*: Neal Morgan, Carolyn Hutchinson, Teresa McKinley; *Ulster Hospital, Dundonald*: Alastair Woodman, Judi Graham, Niall Leonard, John Smyth, Vicki Adell, Samantha Hagan; *Royal Free London NHS Foundation Trust*: Ben Caplin, Amin Oomatia, Eleanor Damian, Toluleyi Sobande; *Kent & Canterbury Hospital*: Tim Doulton, Michael Delaney, Mahmoud Montasser, Jenny Hansen, David Loader, Angela Moon, Frances Morris; *Salford Royal NHS Foundation Trust*: Smeeta Sinha, Chukwuma Chukwu, Amy Hudson, Diane Campbell, Melanie Kershaw, Stephanie Whittaker; *Brighton and Sussex University Hospital's NHS Trust*: Ayesha Irtiza-Ali, Farid Ghalli, Heba Nosseir, Allison Leslie, Kate Trivedi; *University Hospital of Wales, Cardiff*: Donald Fraser, Mohammad Alhadj Ali, Sian Griffin, Farah Latif, Justyna Witczak, Alexa Wonnacott, Lynda Jeffers, Yvette Webley; *Edinburgh Royal Infirmary*: Paul Phelan, Eve Miller-Hodges, Ailsa Geddes, Margaret Glenwright; *Gloucestershire Hospitals NHS Foundation Trust*: Thomas Pickett, Jim Moriarty, Linda Hill, Amanda Tyler; *University Hospitals Coventry and Warwickshire*: Waqar Ayub, Gail Evans, Sue Hewins, Davina Hewitt, Kerry Read; *Ninewells Hospital*: Samira Bell, Rachel Craik, Shona Murray; *Royal Berkshire Hospital, Reading*: Nitin Bhandary, Holly Coles, Rashmi Easow, Maya Joseph; *Northern General Hospital, Sheffield*: Arif Khwaja, Yvonne Jackson, Angeline Mbuyisa, Rachel Sellars; *Darent Valley Hospital, Dartford*: Nihil Chitalia, Cynthia Mohandas, Anca Gherman, Charlotte Kamundi, Olumide Olufuwa; *Royal London Hospital*: Kieran McCafferty, Adedolapo Adeleke, Cara Healy, Damini Jeyarajah, Edward Kinsella-Perks; *Ipswich Hospital*: Richard Smith, Brian Camilleri, Carol Buckman, Jenny Finch, Vanessa Rivers; *University Hospitals Plymouth NHS Trust*: Andrew Connor, Sheila Carr, Lisa Shainberg; *Cheltenham General Hospital*: Thomas Pickett, Linda Hill, Amanda Tyler; *St. James's University Hospital, Leeds*: Andrew Lewington, Richard Baker, Suzannah Dorey, Kay Tobin, Rosalyn Wheatley; *St. George's University Hospitals NHS Foundation Trust*: Debasish Banerjee, Richard Hull, Sharirose Abat, Riny Paul; *Norfolk and Norwich University Hospitals*: Mahzuz Karim, Zay Htet, Rotimi Oluyombo, Saad Tufail, Ravi Varma, Karen Convery, Deirdre Fottrell-Gould, Lisa Hudig, Emily Tropman; *Walsall Healthcare NHS Trust*: Thahir Abdul-Samad, Anne Grace, Marie Phipps; *St Helier Hospital, Carshalton*: Rebecca Suckling, Subash Somalanka, Bhrigu Sood, Pauline Swift, Sarah Acheampong, Kwame Ansu, Martia Augustin; *Wessex Kidney Centre, Queen Alexandra Hospital, Portsmouth*: Anna Sampson, Lynn Vinall, Kim Wren; *St Bartholomew's Medical Centre*: Shamila Wanninayake, Nicholas Wooding, Heather Edwards, Lydia Owen; *Antrim Area Hospital*: Stephanie Bolton, Marion Carson, Michael Matthews; *University Hospitals of Leicester*: Nigel Brunskill, Jorge Jesus-Silva, Alex Howson, Mary Quashie-Akponeware; *North Middlesex University Trust Diabetes Department, North Middlesex University Hospital*:

Hilary Tindall, Chidambaram Nethaji, Helen Eldon; *Glasgow Clinical Research Facility, Queen Elizabeth University Hospital*: Rajan Patel, Patrick Mark, Alastair Rankin, Michael Sullivan, Kirsty Forsyth, Rowan McDougall; *Great Western Hospital, Swindon*: Tanaji Dasgupta, Louisa Davies, Maggie Ryder; *Hathaway Medical Centre, Chippenham*: Philip Grimmer, Clare Macdonald, Mary Webster; Newcastle: Timothy Ellam, Edwin Wong, Christine Meshykhi, Andrea Webster, Peter Wilson; *Lister Hospital*: Enric Vilar, Jocelyn Berdeprado, Eunice Doctolero, Lily Wilkinson; *Altnagelvin Hospital, Western Health & Social Care Trust*: Frank McCarroll, Hesham Ammar, Ying Kuan, Conor Moran, Girish Shivashankar, Ryan Campbell, Deborah Glowski, Paula McDermott; *Oakenhurst Medical Practice, Blackburn*: Amar Ali, Zuber Patel, Christine Bond, Gillian Whalley

China: *National Clinical Research Center of Kidney Diseases, Jinling Hospital, Nanjing University School of Medicine*: Haitao Zhang, Peiling Chen, Yu An, Yinghua Chen, Liu Yang, Lihua Zhang, Tingting Kan, Ling Zhu; *The Second Affiliated Hospital of Army Medical University, PLA*: Jinghong Zhao, Weiping Hou, Jing Wu; *Beijing Anzhen Hospital, Capital Medical University*: Hong Cheng, Weijing Bian, Zhirui Zhao; *Henan Provincial People's Hospital*: Fengmin Shao, Huixia Cao, Xiaojing Jiao, Peiyuan Niu; *Shanghai Fifth People's Hospital, Fudan University*: Jianying Niu, Yu Chen, Lihong Zhang; *Huazhong University of Science and Technology Union Shenzhen Hospital*: Shenglang Zhu, Jiehui Chen, Ying Jiang, Haiyan Lin, Shaopeng Yao; *The second affiliated hospital of Zhejiang University School of Medicine*: Ying Hu, Huaying Xiao, Fuye Yang; *Shenzhen People's Hospital*: Xinzhou Zhang, Baochun Guo, Qiu Jin, Lixia Liu; *Xiangya Hospital, Central South University*: Xiangcheng Xiao, Ting Meng, Yanyun Xie; *Wuhan Fourth Hospital*: Chuanwen Xu, Jie Huang; Suzhou Kowloon Hospital: Weixin Kong, Qianpan Liu, Xiaoliang Wang; *Jinzhou Central Hospital*: Xueying Wang, Ming Gao; *Zhuzhou Central Hospital (Nephrology)*: Xiumei Hu, Ying Lu; *Sichuan Provincial People's Hospital*: Li Wang, Kun Peng, Wei Wang; *Fuwai Hospital, Chinese Academy of Medical Sciences*: Qiuhong Gong, Jianfang Cai, Xiaojue Li, Xuejiao Liu, Haitao Zhang, Shuhan Zhou; *Zhuzhou Central Hospital (Endocrinology)*: Hong Liu, Shuai Tang, Yao Weng, Yao Yao; *The Central Hospital of Wuhan*: Shi Zhao, Chen Cheng, Wei Wei

Malaysia: *Hospital Kajang*: Sadanah Aqashiah Mazlan, Alia Zubaidah Bahtar, Elliyyin Katiman, Noraini Othman; *Hospital Tuanku Ja'afar*: Lily Mushahar, Nurdiana Mazlan, Nur Sharafina Safiee, Sarasa Ramasamy; *Hospital Selayang*: Hin Seng Wong, Hajar Ahmad Rosdi, Esther Zhao Zhi Tan, Ju Fan Tay; *Hospital Taiping*: Kok Seng Teng, Hasnah Yahaya; *Hospital Sultanah Aminah*: Wen Jiun Liu, Lik Wee Ee, Kenneth Kay Leong Khoo, Yuana Mohd Yusoff; *Hospital Tengku Ampuan Afzan*: Fariz Safhan Mohamad Nor, Mohd Kamil Ahmad, Mohd Ramli Seman; *Hospital Umum Sarawak*: Clare Hui Hong Tan, Laura Lui Sian Ngu, Jaime Yoke May Chan, Javelin Peji; *Hospital Raja Permaisuri Bainun*: Chek Loong Loh, Yee Yan Lee, Sridhar Ramanaidu, Kah Mean Thong, Yik Hong Wong, Suria Junus; *Hospital Sultanah Bahiyah*: Chen Hua Ching, Mohammad Faisal Asmee, Ku Ruziana Ku Md Razi, Chun Leong Low, Christopher Sze Bing Sim, Zhang Duan Tham, Noor Kamila Abdullah; *Hospital Sultan Abdul Halim*: Tai Meng Chen, Yong Chieh Chan, Eason Chang, Huan Yean Kang, Kai Quan Lee, Sue Ann Lee, Aik Kheng Lee, Jeevika Vinathan; *Universiti Kebangsaan Malaysia Medical Centre*: Rizna Abdul Cader, Ruslinda Mustafar, Lydia Kamaruzaman, Rozita Mohd, Rahimah Ismail; *Hospital Kulim*: Chong Men Leong, Chee Koon Low, Liang Wei Wong, Norlezah Adnan, Sabariah Ibrahim; *Hospital Kuala Lumpur*: Mohamad Zaimi Abdul Wahab, Sunita Bavanandan, Yik Shen Lim, Zhang Duan Tham, Wan Hazlina Wan Mohamad, Siti Munirah Jaafar, Nur Ashykeen Mohd Fauzi, Aziee Sudin; *University Malaya Medical Centre*: Soo Kun Lim, Chye Chung Gan, Albert Hing, Wan Ahmad Faizal Alaidin Razali; *Hospital Pulau Pinang*: Yew Fong Liew, Chelsia Bao Tyng Chan, Mei Chih Cheng, Yu Chen Ong, Loke Meng Ong, Farah Amalina Mohamed Affandi; *Hospital Melaka*: Korina Rahmat, Ban Chai Peng, Masayu Amat; *Hospital Pakar Sultanah Fatimah*: Nuzaimin Hadafi Ahmad, Doo Yee Mah, Yi Loon Tye, Zaid Azhari, Siti Nabilah Mohamad Zaini, Mohd Aidil Musa; *Hospital Ampang*: Norazinizah Ahmad Miswan, Rafizanur Ramli, Nor Aziah Ahmad; *Hospital Serdang*: Bak Leong Goh, Nurul Izah Ahmad, Fairol Huda Ibrahim, Tze Jian Ng,

Malini Shanmuganathan, Li Lian Tay; *Hospital Sultanah Nur Zahirah*: Zaiha Harun, Salmi Ramli, Nurul 'Ain Yusof, Rossenizal Abd Rahman; *Pusat Perubatan UiTM*: Muhammad Iqbal Abdul Hafidz, Nur Hidayati Mohd Sharif, Irda Yasmoon Awang

Japan: *Chubu Rosai Hospital*: Eitaro Nakashima, Rui Imamine, Makiko Minatoguchi, Yukari Miura, Miduki Nakaoka, Yoshiki Suzuki, Hitomi Yoshikawa; *Shin Clinic*: Koki Shin, Kanae Fujita, Misuzu Iwasa, Haruka Sasajima, Airi Sato; *Kansai Electric Power Hospital*: Yoshiyuki Hamamoto, Yuki Fujita, Takuya Haraguchi, Takanori Hyo, Kiyohiro Izumi, Toshiyuki Komiya, Sodai Kubota, Takeshi Kurose, Hitoshi Kuwata, Susumu Nakatani, Kaori Oishi, Saki Okamoto, Kaori Okamura, Jun Takeoka, Nagaaki Tanaka, Katsuya Tanigaki, Naohiro Toda, Koin Watanabe, Hiromi Komori, Rika Kumuji, Asako Takesada, Aya Tanaka; *Nagoya University Hospital*: Shoichi Maruyama, Tomonori Hasegawa, Akiko Ishiguro, Takuji Ishimoto, Kazuhiro Ito, Yutaka Kamimura, Noritoshi Kato, Sawako Kato, Hiroshi Kojima, Tomoki Kosugi, Kayaho Maeda, Masasi Mizuno, Shoji Saito, Yuka Sato, Yasuhiro Suzuki, Akihito Tanaka, Yoshinari Yasuda, Fujiko Hasegawa, Maiko Hayashi, Shizuka Higashi, Kaho Shimamura, Momoko Sumi, Kazuki Tajima, Chimaki Unekawa, Kana Wakayama, Yukiko Wakita; *Ota diabetes clinic*: Takatoshi Otani, Ayako Imai, Sayaka Kawashima, Eri Kogure, Tomoe Sato, Misato Takezawa, Shinya Yoshida; *Fukui Prefectural Hospital*: Hideo Araki, Yuko Katsuda, Masahiro Konishi, Takahiro Matsunaga, Masashi Oe, Kunihiro Ogane, Masato Sakai, Tomoko Takahashi, Takahiro Yamano, Takuya Yokoyama, Hitomi Ito, Masayo Katayama, Emi Kuroda; *Medical Corporation Seijinkai Ikeda Hospital*: Toru Ikeda, Takuma Kojo, Etsuo Yoshidome, Rieko Mizumachi, Akane Yamamoto, Narihisa Yamasaki, Yoshihiko Yamasaki; *Okayama University Hospital*: Jun Wada, Jun Eguchi, Chigusa Higuchi, Akihiro Katayama, Masaru Kinomura, Masashi Kitagawa, Shinji Kitamura, Satoshi Miyamoto, Hiroshi Morinaga, Atsuko Nakatsuka, Ichiro Nojima, Kenichi Shikata, Hitoshi Sugiyama, Katsuyuki Tanabe, Kenji Tsuji, Haruhito Uchida, Mayu Watanabe, Chie Hashimoto, Takahiro Kato, Sayaka Yamamoto; *Tokai University Hospital*: Takehiko Wada, Masafumi Fukagawa, Naoto Hamano, Masahiro Koizumi, Hirotaka Komaba, Yosuke Nakagawa, Michiyo Iwamoto; *Fukuoka University Hospital*: Kosuke Masutani, Akane Katanosaka, Mayu Kiyota, Hikari Uchi, Sonoka Yamamoto; *Kawasaki Medical School Hospital*: Hajime Nagasu, Seiji Itano, Tsukasa Iwakura, Hiroyuki Kadoya, Eiichiro Kanda, Naoki Kashihara, Kengo Kidokoro, Megumi Kondo, Tamaki Sasaki, Minoru Satoh, Atsuyuki Tokuyama, Reina Umeno, Yoshihisa Wada, Toshiya Yamamoto, Yu Yamanouchi, Masumi Abe, Yoko Inukai; *Kobe University Hospital*: Wataru Ogawa, Shunichiro Asahara, Hideki Fujii, Shunsuke Goto, Yushi Hirota, Tetsuya Hosooka, Keiji Kono, Shinichi Nishi, Yuko Okada, Kazuhiko Sakaguchi, Kenji Sugawara, Michiko Takahashi, Tomoko Takai, Yoshikazu Tamori, Kentaro Watanabe, Miyu Kitajima, Misaki Nishi, Junko Wada; *Aichi Medical University Hospital*: Yasuhiko Ito, Hideki Kamiya, Akimasa Asai, Nao Asai, Saeko Asano, Shogo Banno, Yohei Ejima, Hanako Hase, Tomohide Hayami, Tatsuhito Himeno, Takahiro Ishikawa, Mayumi Ito, Shiho Iwagaitsu, Rina Kasagi, Yoshiro Kato, Makoto Kato, Koichi Kato, Takayuki Katsuno, Miyuka Kawai, Hiroshi Kinashi, Masaki Kondo, Masako Koshino, Naoya Matsuoka, Yoshiaki Morishita, Mikio Motegi, Jiro Nakamura, Hiromi Shimoda, Hirokazu Sugiyama, Shin Tsunekawa, Makoto Yamaguchi, Kazuyo Takahashi; *Juntendo University Hospital*: Hirotaka Watada, Takashi Funayama, Yasuhiko Furukawa, Tomohito Gohda, Hiromasa Goto, Hideyoshi Kaga, Yasuhiko Kanaguchi, Akio Kanazawa, Kayo Kaneko, Toshiki Kano, Masao Kihara, Shogo Kimura, Takashi Kobayashi, Masayuki Maiguma, Yuko Makita, Satoshi Mano, Tomoya Mita, Takeshi Miyatsuka, Maki Murakoshi, Masahiro Muto, Masami Nakata, Junichiro Nakata, Yuya Nishida, Nao Nohara, Takeshi Ogihara, Daisuke Sato, Junko Sato, Hiroaki Sato, Yusuke Suzuki, Ruka Suzuki, Hitoshi Suzuki, Miyuki Takagi, Yoshifumi Tamura, Toyoyoshi Uchida, Seiji Ueda, Miki Asawa, Minako Miyaji, Eri Nagashima, Yoshie Shibata, Eri Yanagisawa; *The University of Tokyo Hospital*: Takashi Kadowaki, Toshimasa Yamauchi, Masaomi Nangaku, Yosuke Hirakawa, Hiroshi Nishi, Nobuhiro Shojima, Satoko Horikawa, Yukiko Nakayama, Yamada Naoko, Yuki Omori; *Maebashi Hirosegawa Clinic*: Shintaro Yano, Miyabi Ioka, Nahoko Kuwabara, Remi Nagano, Megumi Nozawa, Yumi Osawa; *Shiga University of Medical Science Hospital*: Hiroshi Maegawa, Shinji Kume, Shinichi Araki, Itsuko Miyazawa, Katsutaro Morino, Ikuko

Kawai, Masumi Sobata, Motoko Takaoka; *Koukan Clinic*: Yasushi Iwaita, Takashi Udagawa, Ami Inamori, Misuzu Iwasa, Aya Kawase, Aya Yamanaka; *University of Tsukuba Hospital*: Hitoshi Shimano, Akiko Fujita, Hitoshi Iwasaki, Hirayasu Kai, Yoshinori Osaki, Chie Saito, Motohiro Sekiya, Ryoya Tsunoda, Kunihiro Yamagata, Rikako Nakamura, Aiko Yamada; *Center Hospital of the National Center for Global Health and Medicine*: Mitsuru Ohsugi, Motoharu Awazawa, Ryotaro Bouchi, Shota Hashimoto, Makiko Hashimoto, Tomoko Hisatake, Noriko Ihana, Koko Ishizuka, Kazuo Izumi, Hiroshi Kajio, Michi Kobayashi, Noriko Kodani, Koji Maruyama, Michihiro Matsumoto, Maya Matsushita, Tomoka Nakamura, Takehiro Sugiyama, Akiyo Tanabe, Aiko Terakawa, Kojiro Ueki, Yuko Orimo, Takako Ozawa, Eriko Takahira; *AMC Nishi-Umeda Clinic*: Yoshimitsu Yamasaki, Masakazu Haneda, Tadahiro Tomita, Saori Akimoto, Akihiro Fujimoto, Kenji Ishihara, Akiyo Nishiyama, Yukiko Toyonaga, Kana Uozumi, Yukihiro Yamaji; *Jyoumou Ohashi Clinic*: Tetsuya Shigehara, Jun Okajyo, Yukihiro Shimizu, Miyabi Ioka, Nahoko Kuwabara, Megumi Nozawa, Yumi Osawa; *Iwasaki internal medicine clinic*: Shingo Iwasaki, Saori Akimoto, Akihiro Fujimoto, Yuki Fukao, Megumi Furusho, Shintaro Nunokawa; *Tohoku University Hospital*: Hideki Katagiri, Tomohito Izumi, Keizo Kaneko, Shinjiro Kodama, Mariko Miyazaki, Yuichiro Munakata, Tasuku Nagasawa, Yuji Oe, Kei Takahashi, Kazushige Hirata, Keiko Inomata, Taeko Uchida, Chigusa Yamashita; *Tokyo-eki Center-building Clinic*: Arihiro Kiyosue, Ryota Tamura

Canada: *CRIUCPQ*: Francois Dube, Marilene Bolduc, Marie-Christine Talbot; *University Health Network-Toronto General Hospital*: David Cherney, Leslie Cham, Vesta Lai, Josephine Tse; *Clinical Research Solutions Inc.*: Shivinder Jolly, Tabbatha Duck; *Interior Health Kelowna General Hospital*: Scott Lyle, Rachel Epp, Camille Galloway, Susan Haskett, Elizabeta Matvienko, Liam Paulsen; *London Health Sciences Centre*: Louise Moist, Kerri Gallo, Zabrina Lozon, Tina Ramsey, Brittany Whitmore; *St Paul's Hospital*: Adeera Levin, Bader Al-Zeer, Paula Macleod, Aoife O'Sullivan, Zainab Sheriff; *Cambridge Cardiac Care Centre*: Amritanshu Pandey, Samantha Armstrong, Bethelihem Gebeyehu, Patrick Toth; *LMC Clinical Research Inc. (Thornhill)*: Ronald Goldenberg, Mahsa Jahangiriesmaili, Shariff Sanguila, Neethi Suresh, Tanvi Talsania; *Vancouver General Hospital*: Nadia Zalunardo, Bader Al-Zeer, Paula Macleod, Aoife O'Sullivan, Zainab Sheriff; *CHU de Quebec-Universite Laval*: Mohsen Agharazii, Marie-Pier Roussel, Annie Saillant, France Samson; *LMC Clinical Research Inc. Brampton*: Harpreet Bajaj, Miken Bhavsar, Parul Dhall, Gagandeep Dhillon, Bhupinder Grewal, Taniya Nimbkar; *CIUSSS Nord de l'ile de Montreal*: Francois Madore, Guylaine Marcotte; *LMC Clinical Research Inc. (Bayview)*: Oren Steen, Mathura Bullen, Shayani Raguwaran, Andre Valleteau; *CIUSSS de l'Estrie-CHUS, Hopital Fleurimont*: Marie-France Langlois, Christine Brown; *Lakeridge Health*: Andrew Steele, Melissa Garrity, Taneera Ghate, Holly Robinson, Michael Tolibas; *LMC Clinical Research Inc. (Ottawa)*: Chetna Tailor, Lauren Elliott; *Fadia El Boreky Medicine Professional*: Fadia Boreky, Sameh Fikry, Ayesha Ali, Chintankumar Barot, Wagdy Basily, Bethelihem Gebeyehu, Thisun Saram; *LMC Clinical Research Inc (Etobicoke)*: Hasnain Khandwala, Patricia Alvarez, Balwinder Gill, Nazihah Huda, Aamir Navivala, Daniel Pinto; *Kidney Care Centre-Fraser Health*: Micheli Bevilacqua, Elaine Fung, Geraldine Hernandez; *Institut de recherches cliniques de Montreal*: Remi Rabasa-Lhoret, Danijela Bovan, Marie Devaux

Italy: *Policlinico San Martino, Genova*: Roberto Pontremoli, Cecilia Barnini, Giovanna Leoncini, Luca Manco, Giulia Nobili; *Ospedale Casa Sollievo della Sofferenza, San Giovanni Rotondo*: Matteo Piemontese, Filippo Aucella, Rachele Grifa, Francesco Totaro; *Policlinico S. Orsola-Malpighi, Bologna*: Gaetano La Manna, Irene Capelli, Giuseppe Cianciolo, Sarah Lerario, Fulvia Zappulo; *Ospedale S. Giovanni di Dio, Firenze*: Alberto Rosati, Filippo Fani, Giuseppe Spatoliatore, Ester Baldini, Francesca Bianchini; *AOU Policlinico, Bari*: Loreto Gesualdo, Francesco Pesce, Maria Russo, Maria Zippo, Cesira Cafiero; *Ospedale Martini, Torino*: Daria Motta, Simona Bianco, Donatella Bilucaglia; *Ospedale Maggiore Policlinico, Milano*: Piergiorgio Messa, Laura Pavone, Federica Tripodi, Simone Vettoretti; *AOU*

Padova: Paola Fioretto, Gianni Carraro, Filippo Farnia, Anna Postal; *Ospedale Sacro Cuore di Gesù, Gallipoli*: Alessandro D'Amelio, Antonio Cardone, Giovanni Piccinni, Annalisa Aloisi; *ASST Spedali Civili, Brescia*: Francesco Scolari, Federico Alberici, Alice Guerini, Chiara Saccà, Chiara Salviani, Roberta Zani; *AOU L. Vanvitelli, Napoli*: Luca De Nicola, Carlo Garofalo, Maria Liberti, Roberto Minutolo, Luigi Pennino, Lucio Polese; AOU *Sant' Andrea, Roma*: Paolo Mené, Simona Barberi, Clorinda Falcone; *Ospedale Ignazio Veris delli Ponti, Scorrano*: Francesco Russo, Maurizio Caroppo; *Ospedale di Circolo, Desio*: Gennaro Santorelli, Rodolfo Rivera; AOU *Policlinico G. Martino, Messina*: Domenico Santoro, Alfio Giuffrida, Fortunata Zirino; *Ospedale Civile SS. Antonio e Biagio, Alessandria*: Cristina Calvi, Luca Estienne; *AOUI, Verona*: Giovanni Gambaro, Concetta Gangemi, Vittorio Ortalda, Giuseppina Pessolano; *Fondazione Policlinico Universitario Agostino Gemelli, Roma*: Giuseppe Grandaliano, Rocco Baccaro, Pietro Ferraro, Roberto Mangiacapra; *IRCCS Ospedale San Raffaele, Milano*: Marco Melandri, Nadia Foligno, Rita Quartagno, Giuseppe Vezzoli, Elena Brioni

Individual Financial Disclosures for the Writing Committee

Writing Committee
William G. Herrington, Christoph Wanner, Jennifer B. Green, Sibylle J. Hauske, Parminder Judge, Kaitlin J. Mayne, Sarah Y.A. Ng, Emily Sammons, Doreen Zhu, Natalie Staplin, David Preiss, Will Stevens, Karl Wallendszus, Rejive Dayanandan, Carol Knott, Michael Hill, Jonathan Emberson, Susanne Brenner, Vladimir Cejka, Alfred K. Cheung, Zhihong Liu, Jing Li, Peiling Chen, Laiseong Hooi, Wen Liu, Takashi Kadowaki, Masaomi Nangaku, Adeera Levin, David Cherney, Roberto Pontremoli, Aldo Pietro Maggioni, Shinya Goto, Aiko Tomita, Rajat Deo, Katherine Tuttle, Jens Eilbracht, Stefan Hantel, Mark Hopley, Martin J. Landray†, Colin Baigent†, Richard Haynes† († denotes joint senior authors)

Disclosures

CTSU at the University of Oxford has a staff policy of not accepting honoraria or consultancy fees (see https://www.ctsu.ox.ac.uk/about/ctsu_honoraria_25june14-1.pdf).
A.L. reported grants to her institution to conduct of EMPA-KIDNEY in Canada from the University of Oxford and Boehringer Ingleheim.
A.T. None.
A.P.M. reported grants to his institution to conduct EMPA-KIDNEY in Italy, and personal fees outside the present work from Bayer, Novartis, AstraZeneca, and Fresenius. He reported participation on a Data Safety Monitoring Board for Bayer and received personal fees to participate at a Data Monitoring Committee, outside the present work. Other financial or non-financial interests include Novartis, AstraZeneca and Fresenius where personal fees were paid for participation in study committees, outside the present work.
A.K.C reported grants to his institution from the National Institutes of Health and US Department of Veterans Affairs, and personal fees from Boehringer Ingelheim, UptoDate, Tricidia, Bart, Amgen, FibroGen, and Merck. He reported consultancy payments from Boehringer Ingleheim, and has received grants or contracts from National Institutes of Health, and salary support from the U.S. Department of Veterans Affairs. He reported consulting fees from UptoDate, Tricida, Bart, Amgen, FibroGen, National Institutes of Health, and support for attending meetings and/or travel from the National Institutes of Health, U.S. National Kidney Foundation. He participates on a Data Safety Monitoring Board or Advisory Boards for the National Institutes of Health and has stock or stock options in Merck.
C.K. None.
C.W. reported grants to his institution from the University of Oxford and Boehringer Ingelheim and that he received consulting fees from Amicus, Astellas, AstraZeneca, Akibia, Bayer, Boehringer Ingelheim, Chiesi, Gilead, GSK, Idorsia, Novartis, MSD, Sanofi-Genzyme, Tricida, and Vifor. He has also received payments or honoraria for lectures, presentations, speakers bureaus, manuscript writing or educational events from AstraZeneca, Bayer, Boehringer Ingelheim, Eli Lilly, Fresenius Medical Care, Shire-Takeda. He is the current President of the European Renal Association.
C.B. reported that Boehringer Ingelheim provides support through a grant to the University of Oxford for the EMPA-KIDNEY trial, and has received institutional grants for the following: Medical Research Council UK as director of the MRC-Population Health Research Unit 2019-24; Therapy Acceleration Laboratory Award 2021; NIHR HTA - 17/140/02: Cost-effectiveness of statin therapies evaluated using individual participant data from large randomised clinical trials 2019-22; Health Data Research UK - Substantive Site award 2018-23. He currently has Steering Committee roles for trials funded by Merck, the NIHR HTA, and the British Heart Foundation and chairs the European Society of Cardiology Committee on Practice Guidelines.
D.C. reported grants to his institution from Boehringer Ingelheim and Lilly, Merck, Janssen, Sanofi, AstraZeneca and Novo-Nordisk. He has received consulting fees from Boehringer Ingelheim and Lilly, Merck, AstraZeneca, Sanofi, Janssen, Bayer, BMS, Maze, CSL-Behring, and Novo-Nordisk. He also received payments or honoraria for lectures, presentations, speakers bureaus, manuscript writing or educational events from Boehringer Ingelheim and Lilly, Merck, AstraZeneca, Mitsubishi-Tanabe, Janssen, Bayer and Novo-Nordisk, and educational support to his institution from Boehringer Ingelheim and Eli Lilly, AstraZeneca, Janssen and Bayer.
D.P. reported grants to the University of Oxford from Boehringer Ingleheim and Novo Nordisk, and receipt of provision of study drug (fenofibrate/placebo) for the LENS trial.
D.Z. – None.
E.S. reported that her institution is in receipt of a grant from the British Heart Foundation. Her non-financial entity interests include Health Intelligence Ltd and Northgate Ltd who are contracted with the University of Oxford on a data linkage project.
J.B.G. reported support through grants paid to her institution by Boehringer Ingleheim and Lilly, Sanofi/Lexon, Roche, AstraZeneca and Merck. She has received consulting fees from Boehringer Ingelheim and Lilly, Hawthorne Effect/Omada, AstraZeneca, Bayer, Pfizer, and Sanofi/Lexicon. She has previously participated on a Data Safety Monitoring Board for Novo Nordisk, and has leadership or fiduciary roles on other board, society, committee or advocacy groups including being a Member of the American Diabetes Association's Professional Practice Committee.
Je.E. reported he is a full-time employee of Boehringer Ingelheim, the sponsor of the EMPA-KIDNEY trial.
Jo.E reported that Boehringher Ingleheim provides support through a research grant to the University of Oxford to conduct the EMPA-KIDNEY trial.
J.L. reported a grant from the University of Oxford for conducting the EMPA-KIDNEY trial to her institution (Fuwai Hospital).

K.J.M. – None.
K.W. reported that Boeringher Ingleheim and Eli Lily provided an Institutional Grant for conduct of EMPA-KIDNEY.
K.T. reported grants were paid to her institution by the NIH, and awards from the CDC, Goldfinch Bio, Bayer and Travere. She has received consulting fees from Boeringher Ingleheim, Astra Zeneca, Goldfinch Bio, Travere, Novo Nordisk and Bayer, the NIH, and George Clinical - Institution. She has leadership or fiduciary role in other board, society, committee or advocacy group, paid or unpaid including being on the Diabetic Kidney Disease Collaborative Task Force -Institution, American Society of Nephrology.
L.H. – None.
Ma.H. reported that he is a full-time employee of Boehringer Ingelheim, the sponsor of the EMPA-KIDNEY trial.
M.J.L. reported that Boehringer Ingelheim provides funding to his institution to support the clinical trial, and grants were paid to his institution by Novartis to support a clinical trial and from Janssen for a fellowship programme. He reports Regeneron and Roche provided a donation of treatment for clinical trials.
M.N. reported that Boehringher Ingleheim is financially supporting the EMPA-KIDNEY trial and that he has received advisory fees about drug development from Boeringher Ingleheim. He has leadership or fiduciary role in other board, society, committee or advocacy group, paid or unpaid include being a Vice President of the Japanese Society of Internal Medicine, President of the Asian Pacific Society of Nephrology and President-Elect of the International Society of Nephrology.
Mi.H. reported that the EMPA-KIDNEY trial was funded by Boehringer Ingelheim. The Clinical Trial Service Unit at the University of Oxford receives support from the UK Medical Research Council (which funds the MRC Population Health Research Unit in a strategic partnership with the University of Oxford), the British Heart Foundation and Cancer Research UK. He reports payments from Novartis to his institution (University of Oxford).
N.S. reported grants from Boehringer Ingleheim and Novo Nordisk paid to her institution.
P.J. – None.
P.C. – None.
Ra.D. reported that Boehringer Ingleheim provides funding to his institution to support the clinical trial, and grants were paid to his institution by National Institute of Health. He reports payments from Boehringer Ingleheim, including support for attending meetings and/or travel.
Re.D. – None.
R.H. reported that Boehringer Ingelheim provided an Institutional Grant for the EMPA-KIDNEY trial. He reports institutional grants from Novartis, and supply of drug for trials from Roche and Regeneron.
R.P. reported that he received payments or honoraria for lectures, presentations, speakers bureaus, manuscript writing or educational events from Astra Zeneca, Boehringher Ingleheim, Lilly, Novartis, MSD and Alfasigma
S.Y.A.N. – None.
S.G. reported that Boehringer Ingleheim is financially supporting the EMPA-KIDNEY trial. He reported a grant from Vehicle Racing Commemorative Foundation (6236). He has received consulting fees were from Bristol Myer Squibb, Jansen Pharma and Cantos. He has leadership or fiduciary role in other board, society, committee or advocacy groups, paid or unpaid, including being President for The Japenese Society of Biorheology and Vice President for The Japanese College of Angiology.
S.J.H. reported that Boehringer Ingelheim International GmbH is her full time employer.
S.H. reported that Boehringer Ingelheim Pharma GmbH & Co. KG is his employer.
S.B. – None.
T.K. reported grants were to his institution from Nippon Boehringer Ingelheim Co., Ltd., Eli Lilly Japan K.K., Kyowa Kirin Co., Ltd., MSD Corporation, Daiichi Sankyo Co., Ltd., Novo Nordisk Pharma Ltd., Sanofi K.K., Takeda Pharmaceutical Co., Ltd., Astellas Pharma Inc., Ono Pharmaceutical Co., Ltd., Mitsubishi Tanabe Pharma Corporation, Sumitomo Dainippon Pharma Co., Ltd. and Asahi Mutual Life Insurance Company. He has received payments or honoraria for lectures, presentations, speakers bureaus, manuscript writing or educational events from MSD Corporation, Takeda Pharmaceutical Co., Ltd., Mitsubishi Tanabe Pharma Corporation, Astellas Pharma Inc., Abbott Japan LLC, Terumo CO., Teijin Pharma Ltd., Ono Pharmaceutical Co., Ltd., Astra Zeneca K.K, Sumitomo Dainippon Pharma Co., Ltd., Sanofi K.K., Eli Lilly Japan K.K., Nippon Boehringer Ingelheim Co., Ltd, Novo Nordisk Pharma Ltd, Bayer Holding Ltd, Novartis Pharma K.K., Daiichi Sankyo Co., Ltd., FUJIFILM Toyama Chemical Co., Ltd., Kowa Co., Ltd., Kyowa Kirin Co., Ltd., and Medtronic Japan Co., Ltd.
V.C. reported support for attending meetings and/or travel from the University of Oxford.
W.L. – None.
W.S. – None.
W.G.H. reported grants to his institution from Boehringer Ingelheim and EliLilly for the EMPA-KIDNEY trial, and grants were paid to his institution from the UK Medical Research Council and Kidney Research UK. He participates unpaid on a data safety monitoring board for Bayer. He has leadership or fiduciary role in other board, society, committee or advocacy group, unpaid, including the European Society of Cardiology, the UK Kidney Association, and NIHR-HTA.
Z.L. – None.
