# Supplementary material for: Design, recruitment, and baseline characteristics of the EMPA-KIDNEY trial
Source: Nephrol Dial Transplant. Author manuscript; Available in PMC 2022 Jun 25. (PMC9217655; doi:10.1093/ndt/gfac040)
Supplement: Appendix [file EMS143886-supplement-Appendix.pdf]

APPENDIX

The EMPA-KIDNEY Collaborative Group

Writing Committee

William G. Herrington, Christoph Wanner, Jennifer B. Green, Sibylle J. Hauske, Parminder Judge, Kaitlin J. Mayne, Sarah Y.A. Ng, Emily Sammons, Doreen Zhu, Natalie Staplin, David Preiss, Will Stevens, Karl Wallendszus, Rejive Dayanandan, Carol Knott, Michael Hill, Jonathan Emberson, Susanne Brenner, Vladimir Cejka, Alfred K. Cheung, Zhihong Liu, Jing Li, Peiling Chen, Laiseong Hooi, Wen Liu, Takashi Kadowaki, Masaomi Nangaku, Adeera Levin, David Cherney, Roberto Pontremoli, Aldo Pietro Maggioni, Shinya Goto, Aiko Tomita, Rajat Deo, Katherine Tuttle, Jens Eilbracht, Stefan Hantel, Mark Hopley, Martin J. Landray[†], Colin Baigent[†], Richard Haynes[†] ([†]denotes joint senior authors).

Writing Committee affiliations

Affiliations of the Writing Committee are as follows: Clinical Trial Service Unit and Epidemiological Studies Unit, Nuffield Department of Population Health, University of Oxford, Oxford, UK (*also part of the Medical Research Council Population Health Research Unit at the University of Oxford): W.G.H.*, P.J., K.J.M., S.Y.A.N., E.S., D.Z., N.S.*, D.P.*, W.S., K.W., R.D., C.K., Mi.H.*, J.E.*, M.J.L.*, C.B.* and R.H.*; University Clinic of Würzburg, Würzburg, Germany: C.W., S.B. and V.C.; Duke Clinical Research Institute, Durham, NC, USA: J.B.G.; University of Utah, Salt Lake City, UT, USA: A.K.C.; Boehringer Ingelheim International: S.J.H., J.E., S.H. and Ma.H.; Vth Department of Medicine, University Medical Center Mannheim, University of Heidelberg, Mannheim, Germany: S.J.H.; National Clinical Research Center of Kidney Diseases, Jinling Hospital, Nanjing University School of Medicine, Nanjing, China: Z.H.L. and P.L.C.; Fuwai Hospital, Chinese Academy of Medical Sciences, National Center for Cardiovascular Diseases, Beijing, China: J.L.; Hospital Sultanah Aminah, Johor Bahru, Malaysia: L.S.H. and W.L.; University of Tokyo School of Medicine, Tokyo, Japan: T.K. and M.N.; Tokai University School of Medicine, Isehara, Japan: S.G. and A.T.; University of British Columbia, Vancouver, BC, Canada: A.L.; University of Toronto, Toronto, ON, Canada: D.C.; Università degli Studi and IRCCS Ospedale Policlinico San Martino di Genova, Genova, Italy: R.P.; ANMCO Research Center, Florence, Italy: A.M.; University of Pennsylvania Perelman School of Medicine, Philadelphia, PA, USA: R.D.; Providence Health Care and University of Washington, Washington, WA, USA: K.T.

Members of the EMPA-KIDNEY Collaborative Group

Executive Committee

Colin Baigent (Co-Chair), Martin J. Landray (Co-Chair), Christoph Wanner (Deputy Chair), William G. Herrington (Chief Investigator), Richard Haynes (Co-Principal Investigator), Jennifer B. Green, Sibylle J. Hauske*, Martina Brueckmann*, Mark Hopley* (previous members: Maximillian von-Eynatten* and Jyothis George*).

Steering Committee

Executive Committee members plus national representatives: Susanne Brenner (Germany); Alfred K. Cheung (USA); David Preiss (UK); Zhihong Liu, Jing Li (China); Laiseong Hooi, Wen Liu (Malaysia); Takashi Kadowaki, Masaomi Nangaku (Japan); Adeera Levin, David Cherney (Canada); Roberto Pontremoli, Aldo Pietro Maggioni (Italy); plus statistician members: Natalie Staplin, Jonathan Emberson, Stefan Hantel*; plus other expert members: Shinya Goto, Rajat Deo, Katherine Tuttle. Non-voting members: Parminder Judge, Sarah Y.A. Ng, Francisco Javier Rossello Lozano, Emily Sammons, Doreen Zhu (*denotes a Boehringer Ingelheim employee).

Independent Data Monitoring Committee

Peter Sandercock (Chair), Rudolf Bilous, Charles Herzog, Paul Whelton, Janet Wittes, Derrick Bennett (non-voting statistician).

Central Coordinating Office's Senior Management Team based at the Clinical Trial Service Unit and Epidemiologial Studies Unit, Nuffield Department of Population Health, University of Oxford

Andy Burke, Richard Brown, Rejive Dayanandan, Lucy Fletcher, Hannah Gosling, Emily Harding, Richard Haynes, William G. Herrington, Parminder Judge, Carol Knott, Ryonfa Lee, Kevin Murphy, Yanru Qiao, Rachel Raff, Hui Yu.

Regional Coordinating Centre Administrative Leadership and Clinical Support

YanRu Qiao (UK); Vladimir Cejka, Marcela Fajardo-Moser (Germany); Andrea Lorimer, Donata Lucci (Italy); Anita Hepditch (US); Amanda Axler (Canada); Peiling Chen, Dai Hao (China), Cheng Beng Goh, Sarojini Sivanandam (Malaysia); Akiko Hashimoto, Wakako Negoro, Aiko Tomita, Morisaki Tomoko (Japan).

EMPA-KIDNEY Collaborators

See Supplementary material for a full listing of collaborators.

Downloaded from https://academic.oup.com/ndt/advance-article/doi/10.1093/ndt/gfac040/6541871 by The Librarian. user on 18 March 2022

49. Perkovic V, Koitka-Weber A, Cooper ME *et al.* Choice of endpoint in kidney outcome trials: considerations from the EMPA-REG OUTCOME® trial. *Nephrol Dial Transplant* 2020; 35: 2103–2111
50. Oshima M, Neal B, Toyama T *et al.* Different eGFR decline thresholds and renal effects of canagliflozin: data from the CANVAS program. *J Am Soc Nephrol* 2020; 31: 2446–2456
51. Neuen B, Tighiouart H, Heerspink H *et al.* Acute treatment effects on GFR in randomized clinical trials of kidney disease progression. *J Am Soc Nephrol* 2022; 33: 291–303

Received: 14.12.2021; Editorial decision: 4.2.2022

Downloaded from https://academic.oup.com/ndt/advance-article/doi/10.1093/ndt/gfac040/6541871 by The Librarian. user on 18 March 2022
